# Infection cushions of *Fusarium graminearum* are fungal arsenals for wheat infection

**DOI:** 10.1111/mpp.12960

**Published:** 2020-06-23

**Authors:** Michael Mentges, Anika Glasenapp, Marike Boenisch, Sascha Malz, Bernard Henrissat, Rasmus J.N. Frandsen, Ulrich Güldener, Martin Münsterkötter, Jörg Bormann, Marc‐Henri Lebrun, Wilhelm Schäfer, Ana Lilia Martinez‐Rocha

**Affiliations:** ^1^ Molekulare Phytopathologie Institut für Pflanzenwissenschaften und Mikrobiologie Universität Hamburg Hamburg Germany; ^2^ UMR CNRS and Aix‐Marseille Université Marseille France; ^3^ Department of Biotechnology and Biomedicine Technical University of Denmark Kgs. Lyngby Denmark; ^4^ Department of Bioinformatics Technical University of Munich TUM School of Life Sciences Weihenstephan Freising Germany; ^5^ Institute of Bioinformatics and Systems Biology München Germany; ^6^ UMR BIOGER INRA AgroParisTech Thiverval‐Grignon France; ^7^ Present address: Functional Genomics and Bioinformatics Sopron University Sopron Hungary

**Keywords:** effector proteins, *Fusarium graminearum*, infection cushion, runner hyphae, secondary metabolites, transcriptome, wheat infection

## Abstract

*Fusarium graminearum* is one of the most destructive plant pathogens worldwide, causing fusarium head blight (FHB) on cereals. *F*.* graminearum* colonizes wheat plant surfaces with specialized unbranched hyphae called runner hyphae (RH), which develop multicelled complex appressoria called infection cushions (IC). IC generate multiple penetration sites, allowing the fungus to enter the plant cuticle. Complex infection structures are typical for several economically important plant pathogens, yet with unknown molecular basis. In this study, RH and IC formed on the surface of wheat paleae were isolated by laser capture microdissection. RNA‐Seq‐based transcriptomic analyses were performed on RH and IC and compared to mycelium grown in complete medium (MY). Both RH and IC displayed a high number of infection up‐regulated genes (982), encoding, among others, carbohydrate‐active enzymes (CAZymes: 140), putative effectors (PE: 88), or secondary metabolism gene clusters (SMC: 12 of 67 clusters). RH specifically up‐regulated one SMC corresponding to aurofusarin biosynthesis, a broad activity antibiotic. IC specifically up‐regulated 248 genes encoding mostly putative virulence factors such as 7 SMC, including the mycotoxin deoxynivalenol and the newly identified fusaoctaxin A, 33 PE, and 42 CAZymes. Furthermore, we studied selected candidate virulence factors using cellular biology and reverse genetics. Hence, our results demonstrate that IC accumulate an arsenal of proven and putative virulence factors to facilitate the invasion of epidermal cells.

## Graphical abstract

Transcriptome analyses on epiphytic runner hyphae (RH) and penetrating infection cushions (IC) of *Fusarium graminearum* grown on wheat palea compared to in culture‐grown mycelium (MY) reveal that IC are fungal arsenals for infection.

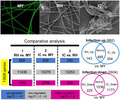

## 1 INTRODUCTION

Fusarium head blight (FHB), caused by *Fusarium graminearum*, is a devastating disease of cereals including wheat, barley, oats, and rye with large economic impacts (Savary *et al*., 2012). After infection and colonization of wheat heads, *F*.* graminearum* reduces wheat yield by interfering with kernel development and by poisoning the remaining kernels with a cocktail of mycotoxins, such as deoxynivalenol (DON) and zearalenone, rendering them unsuitable for food and feed usage (Takemura *et al*., 2007). To date, there are no wheat cultivars available that are fully resistant to *F*.* graminearum* infection (Mesterhazy, 1995). Recently, comprehensive transcriptomic analyses of partially resistant and susceptible wheat cultivars inoculated with *F*.* graminearum* were performed to better understand the host molecular response to FHB (Biselli *et al*., 2018; Pan *et al*., 2018; Wang *et al*., 2018). Additionally, a transcriptional profiling approach separated symptomless and symptomatic aspects of the FHB infection and defined subsets of *F*.* graminearum* genes expressed in a single cereal host species or across two or more cereal hosts (Brown *et al*., 2017). Additional transcriptomics‐based studies have been conducted, focusing on later stages of infection, using wheat coleoptiles, wheat spikes, and maize stalks (Lysøe *et al*., 2011; Zhang *et al*., 2012; Zhang *et al*., 2016; Kazan and Gardiner, 2018). To date, we are lacking information on the initial stages of fungal infection, from conidial germination to fungal growth on the plant surface and penetration into the plant epidermal cells. FHB starts when conidia of *F*.* graminearum* adhere to the surface of wheat spikelets with the help of hydrophobin proteins (Quarantin *et al*., 2019). After germination on wheat floral tissues, *F*.* graminearum* grows epiphytically on the plant surface, using specialized unbranched hyphae, called runner hyphae (RH). These RH differentiate multicellular appressoria, called infection cushions (IC), able to produce several penetration events (Boenisch and Schäfer, 2011; Bormann *et al*., 2014). We previously showed that the deletion of *F*.* graminearum* adenylyl cyclase necessary for cAMP production, as well as overexpression of deoxyhypusine hydroxylase, the second activating enzyme of the eukaryotic translation initiation factor 5A, abolish the formation of IC and, therefore, the ability of the fungus to infect wheat (Bormann *et al*., 2014; Martinez‐Rocha *et al*., 2016).

Appressoria of *Magnaporthe oryzae* and *Colletotrichum* species are melanized single cells emerging directly from conidial germ tubes (Perfect *et al*., 1999; Wilson and Talbot, 2009). They are produced due to the perception of a hydrophobic surface and are morphologically very different from *F*.* graminearum* complex IC that differentiate from specialized RH (Boenisch and Schäfer, 2011). Unicellular appressoria have been widely studied at both the histological and the molecular level, making it most probably the best examined fungal structure (O’Connell *et al*., 2012; Soanes *et al*., 2012). Several plant pathogens such as *Botrytis cinerea*, infecting approximately 200 plant species, *Sclerotinia sclerotiorum,* causing white mould mostly on vegetables, and *Rhizoctonia solani*, a wide host range pathogen, penetrate their host plants using complex appressoria similar to *F*.* graminearum* IC (Armentrout *et al*., 1987; Backhouse and Willetts, 1987; Garg *et al*., 2010). Although IC have been histologically described several times in recent decades (Dodman *et al*., 1968; Nikraftar *et al*., 2013), a molecular description of their development is still pending.

In this study we removed the infecting fungal mycelium from the underlying wheat floral tissue and separated RH from IC using laser capture microdissection. We compared transcriptional changes occurring in these specialized fungal cells (RH and IC) to mycelium (MY) grown in complete medium (CM). This transcriptomic analysis allowed the identification of fungal transcripts specifically detected in the specialized fungal structures for epiphytic growth (RH) and IC. In more detail, we analysed genes encoding carbohydrate‐active enzymes, putative secreted effector proteins, and secondary metabolite biosynthetic enzymes. In particular, transcripts detected in IC encode putative virulence factors prior to the invasion of epidermal cells.

## 2 RESULTS

### 2.1 Identification of *F*.* graminearum* epiphytic growth on wheat palea tissue

**Figure 1:**
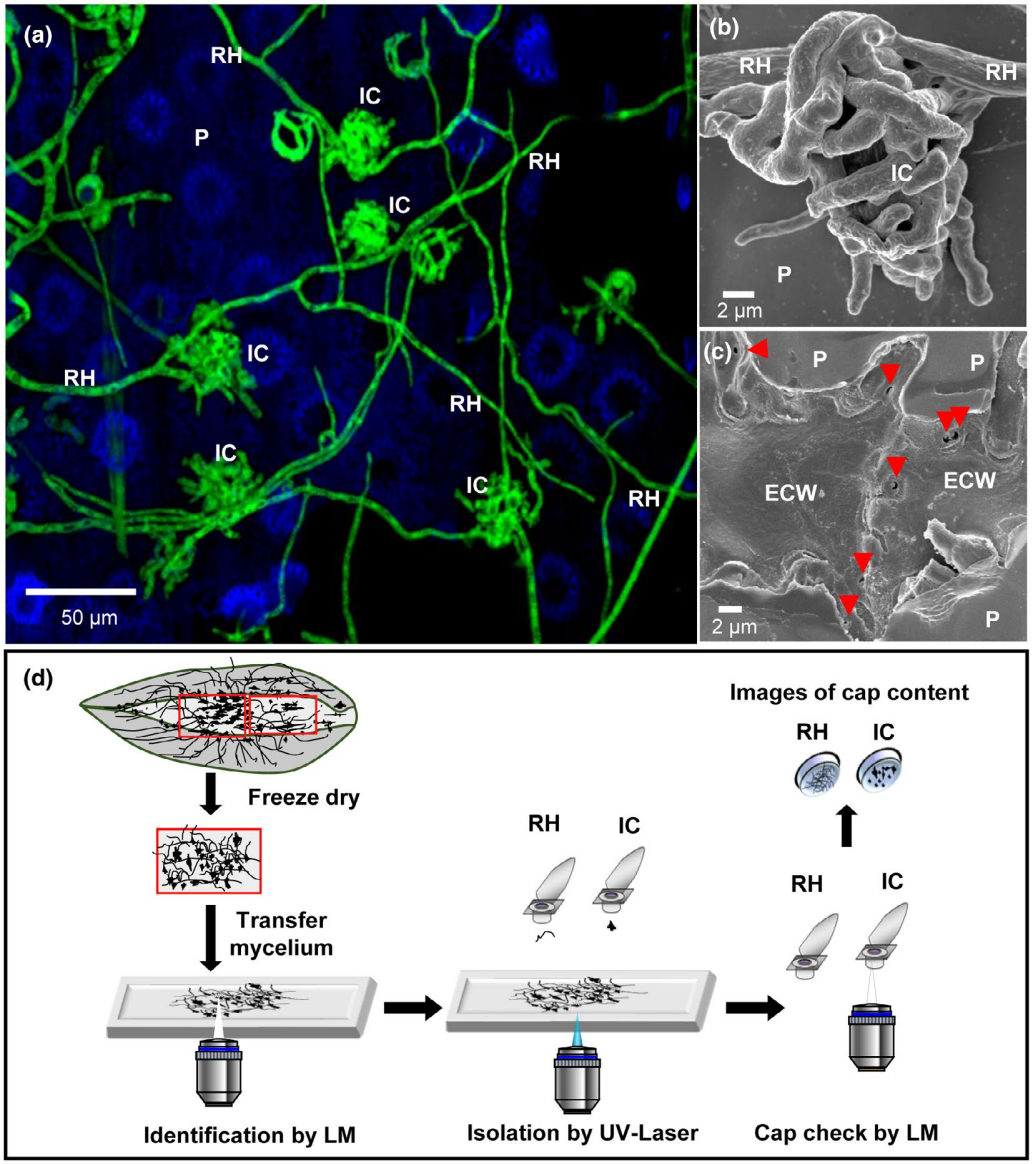
Identification and isolation of infection structures of *F* *usarium* *graminearum* on wheat palea for transcriptome analysis. (a) Confocal image showing *F*.* graminearum* colonizing the plant surface (P) with runner hyphae (RH) and infection cushions (IC). Image is a maximum intensity projection of 30 pictures. (b) Scanning electron microscopy image of an IC developed from RH on the wheat plant surface. (c) Directly underneath a removed IC multiple penetration pores (red arrowheads) of about 1 μm in diameter are visible in the plant epidermal cell wall (ECW). (d) Scheme of isolation of RH and IC by laser capture microdissection. Small samples of paleae containing RH and IC were transferred on an adhesive microscopy slide. RH and ICs were identified and isolated in individual adhesive caps by UV‐laser impulses. The content of the cap was controlled by light microscopy (LM).

Under favourable conditions, conidia germinate and differentiate into specialized RH that epiphytically colonize the surface of wheat (Figure 1a). RH differentiate into IC that are complex appressoria made of agglomerated hyphae (Figure 1a,b). IC facilitate numerous penetration events and colonization of wheat epidermal cells (Figure 1c). Because the formation of IC is necessary for a successful infection, we performed transcriptomic analysis to identify changes in gene expression at the initial stage of wheat paleae infection. RH and IC were detached from the palea surface, separated by laser‐assisted microdissection, and used for RNA extraction (Figure 1d). To identify genes differentially expressed during infection, we also extracted RNA from *F*.* graminearum* hyphae (MY) growing in CM. cDNA libraries of MY, RH, and IC were constructed, Illumina‐sequenced, and mapped against the genome of *F*.* graminearum*. Three replicates of the different libraries, which are highly coherent in a Pearson correlation test (Table S1), were used. The expression patterns of a set of five genes differentially expressed during infection according to RNA‐Seq data were confirmed using quantitative reverse transcription PCR (RT‐qPCR), proving the reliability of the experimental setup (Figure S1). The validated genes are relevant to our study (trichodiene synthase FgTRI5, polyketide synthase FgPKS12, and the putative effector 1 FgPE1) or are required for wheat virulence (GABA transaminases FgGTA1 and FgGTA2; Bönnighausen *et al*., 2015).

### 2.2 Global gene expression profile of *F*.* graminearum* during initial infection of wheat floral tissue

**Figure 2:**
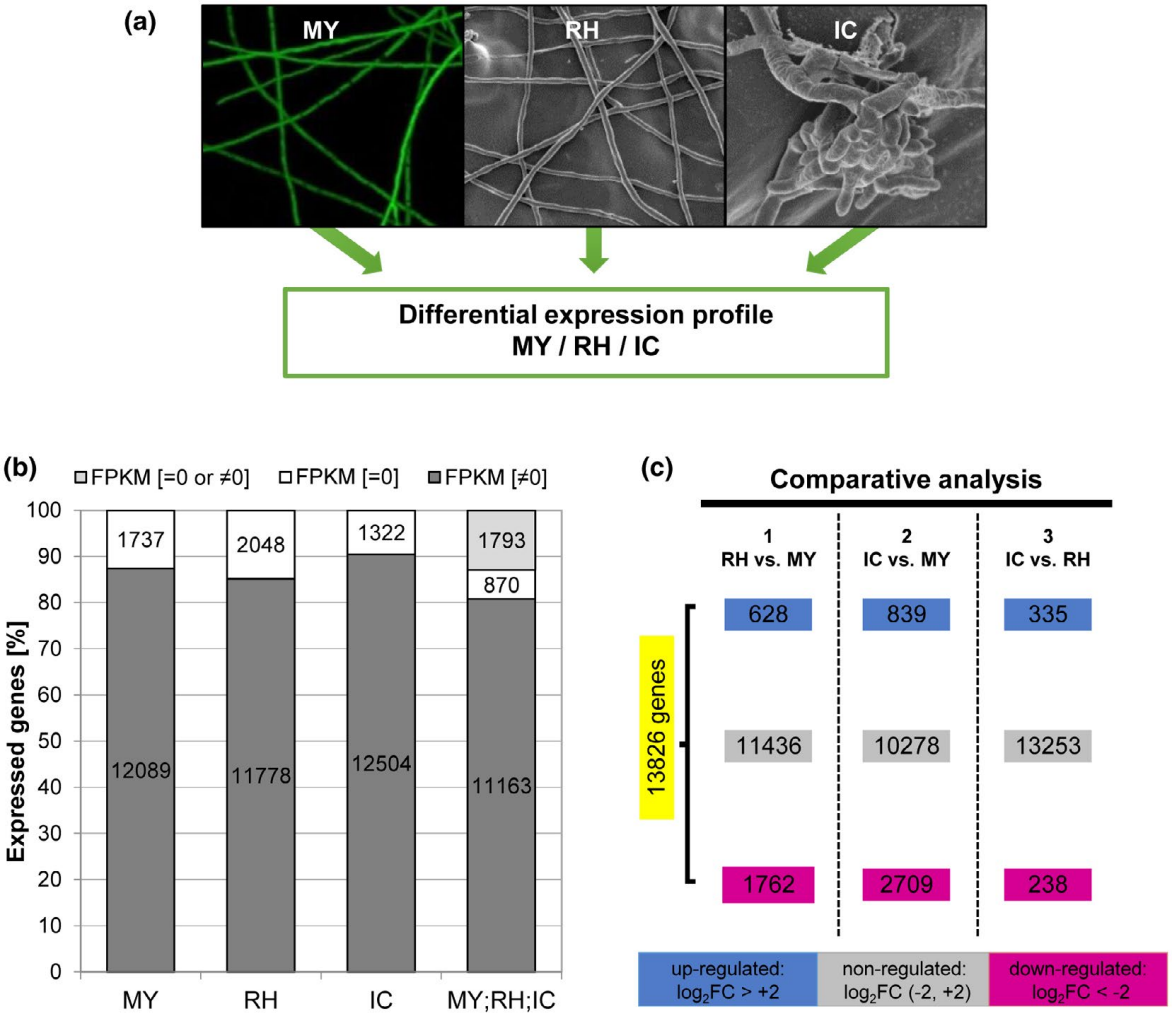
General gene transcription analysis of three *F* *usarium* *graminearum* cell types. (a) Cell types used for transcriptome production: mycelia grown in complete medium (MY), epiphytically growing runner hyphae (RH), and plant‐penetrating infection cushions (IC) on plant surface. (b) Transcribed genes in the different hyphal types or their combination are indicated in dark grey with a nonzero FPKM (FPKM [≠ 0]). Nontranscribed genes are shown in white (FPKM [= 0]). Genes expressed in at least one cell type (FPKM [= 0 or ≠ 0] are represented in light grey. FPKM: fragments per kilobase of exon per million fragments mapped. (c) Comparison of the differentially expressed genes on the different hyphal cell types (total of 13,826 genes). Grey box, nondifferentially regulated genes; magenta box, down‐regulated genes; blue box, up‐regulated genes. The regulation always refers to the first‐mentioned data set in the comparison

Expression patterns of 13,826 predicted genes of *F*.* graminearum* were compared between MY, RH, and IC (Data S1 and Figure 2a). A total of 12,089 (87%), 11,778 (85%), and 12,504 (90%) transcripts were detected in MY, RH, and IC, respectively (Figure 2b); 870 (6%) transcripts were not detected in any cell type (Figure 2b). A comparison of the differentially expressed genes between the three cell types was performed (Figure 2c). Genes with a log_2_ fold change (log_2_ FC) above the threshold of +2 were classified as “up‐regulated”, while genes with a log_2_ FC below −2 were “down‐regulated” and genes with a log_2_ FC between −2 and +2 were “nonregulated”. The major differences were found between IC and MY presenting 839 up‐regulated genes and 2,709 down‐regulated genes (Figure 2c).

**Figure 3:**
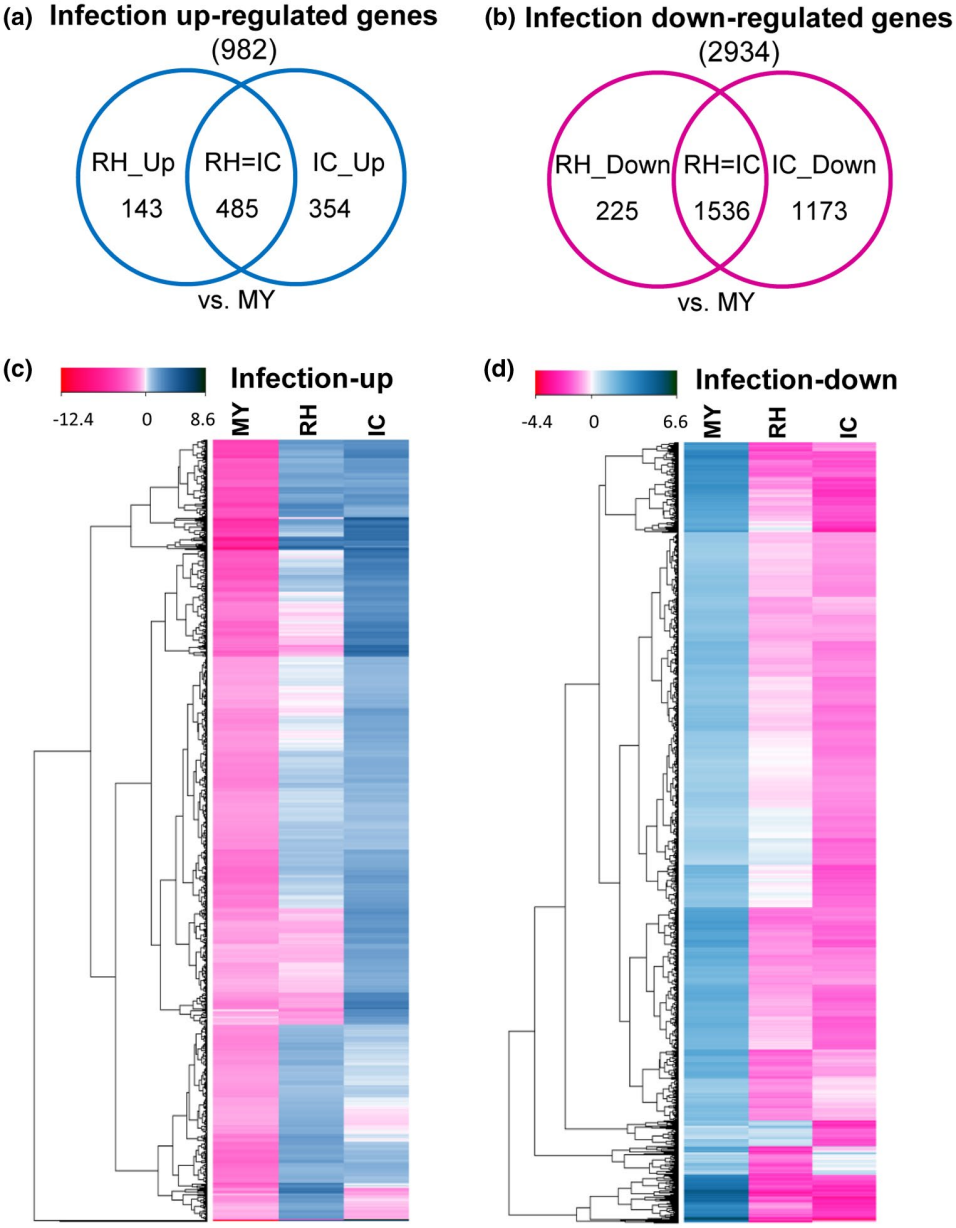
Transcriptional comparison of infection regulated genes in *F* *usarium* *graminearum*. Venn diagrams of (a) infection up‐regulated genes or (b) infection down‐regulated genes, up/down‐regulated in runner hyphae (RH) or infection cushions (IC) compared to mycelium grown in complete medium (MY). (c) Gene expression heat map of infection up‐regulated genes (982) or (d) infection down‐regulated genes (2,934). Transcripts are shown as log_2_ fold change compared to the general mean expression level in all three cell types. Down‐regulated genes are in light magenta to dark magenta and up‐regulated genes are in light blue to dark blue. Equally regulated genes are shown in white

Infection regulated genes were identified by comparing RH and IC expression of genes to MY (Table 1). In total, we identified 3,916 infection regulated genes, further dissected into infection up‐regulated (982) and infection down‐regulated genes (2,934). Most of the infection up‐regulated genes were up‐regulated in both RH and IC (485) or in IC (354, Figure 3a and Table 1). Fewer genes were specifically up‐regulated in RH (143). Similarly, most infection down‐regulated genes were down‐regulated in both RH and IC (1,536) or in IC (1,173) and fewer genes were specifically down‐regulated in RH (225, Figure 3b and Table 1). Heat maps of infection up‐regulated or infection down‐regulated gene expression show not only large differences in expression pattern between RH/IC and MY, but also between RH and IC (Figure 3c,d). To gain an unbiased view of transcriptional changes we identified the top 50 up‐regulated genes in RH compared to MY (Table S2) and the top 50 up‐regulated genes in IC compared to MY (Table S3).

**Table 1:** General gene regulation during palea colonization compared to growth on complete medium

	Regulation	Total	Total (%)	Regulation	Total	Total (%)
(RH or IC) vs. MY (13,826 genes)	Not regulated during infection	9,910	71.68	RH and IC equal	9,804	98.93
				RH up	64	0.65
				IC up	42	0.42
	Infection down‐regulated	2,934	21.22	RH and IC equal	2,759	94.04
				RH up	130	4.43
				IC up	45	1.53
	Infection up‐regulated	982	7.10	RH and IC equal	690	70.26
				RH up	44	4.48
				IC up	248	25.25

Genes were categorized as infection down‐regulated, infection up‐regulated or not regulated by comparing runner hyphae (RH) and infection cushions (IC) to mycelium grown in complete medium (MY). The genes were also separated into differentially expressed genes in RH and IC.

Furthermore, genes were classified as encoding secreted or nonsecreted proteins, and sorted into the following gene families: secondary metabolite biosynthesis gene clusters (SMC; Sieber *et al*., 2014), transcription factors (TF; Son *et al*., 2011), transmembrane receptors (TMR), histone‐modifying proteins (HM), protein kinases/phosphatases (PK), dehydrogenases (DH), carbohydrate‐active enzymes (CAZymes; Lombard *et al*., 2014), genes involved in reactive oxygen species metabolism (ROS), and putative effector (PE) proteins (Data S1 and S2). Heat maps of clustered expression values in MY, RH and IC were generated according to these gene families. While heat maps for SMC, DH, TMR, and PE showed a diverse regulation pattern, genes grouped in nonsecreted proteins, TF, HM, and PK were under‐represented in IC. Heat maps for secreted proteins, CAZymes, and ROS showed more over‐represented genes in IC compared to MY or RH (Figure S2).

Among the 870 transcripts not detected (FPKM = 0), 604 encode proteins with unknown function, 74 are PE, 48 are genes involved in SMC, 36 in ROS, 21 in TP, and 19 in TF, among others (Data S1, sheet TND). Within the infection regulated genes, 634 encode secreted proteins, of which 306 were infection down‐regulated, while 328 were infection up‐regulated genes (Table S4). The highest transcriptional changes on infection regulated secreted proteins were on CAZymes, ROS, and PE (Table S4). Within the 3,378 infection‐regulated nonsecreted proteins, 694 were infection up‐regulated, while 2,684 were infection down‐regulated. Functional categories with the highest transcriptional changes in infection‐regulated nonsecreted proteins were the TF, TP, PK, and ROS (Table S4). In addition, 32 of the 67 known SMC were differentially expressed on infection, with 19 SMC being down‐regulated and 13 SMC being up‐regulated (Table 2). In conclusion, plant colonization triggers a wide range of changes in the *F*.* graminearum* transcriptome.

**Table 2:** Comparative expression analysis of 67 secondary metabolite gene clusters (SMC)

		No. of clusters	Cluster IDs^b^
Infection up‐regulated^a^	RH and IC up‐regulated	6	C03, C21 (triacetyl fusarine), C25, C36, C57, C63 (malonichrome)
	Specifically RH up‐regulated	1	C13 (aurofusarin)
	Specifically IC up‐regulated	5	C16, C22, C23 (trichothecene), C64 (fusaoctaxin A), C66
Similar expression^a^		18	C08, C12, C17, C18 (orcinol), C19, C26, C30, C32, C34, C35, C43, C44, C45, C50, C56, C59 (culmorin), C65, C67
Infection down‐regulated^a^	RH and IC down‐regulated	20	C04, C05, C06, C10, C11, C20, C27, C28 (carotenoid), C29, C31, C33 (ferricrocin), C38, C39, C41, C47, C54, C55, C58, C61, C62
	Specifically RH down‐regulated	2	C37, C40
	Specifically IC down‐regulated	1	C09
Not expressed^a^		14	C01, C02, C07, C14, C15 (zearalenone), C24, C42 (fusarin C), C46 C48, C49 (butenolide), C51, C52, C53 (precursor of insoluble perithecial pigment), C60 (fusarielin)

^a^ Gene clusters expression analysis was determined as mentioned in the methods section, estimation of secondary metabolite gene cluster (SMC) regulation.

^b^ Name of known secondary metabolites is given in parentheses. IC, infection cushions; RH, runner hyphae; MY, mycelium grown in complete medium. Secondary metabolite cluster nomenclature according to Sieber *et al*., 2014.

### 2.3 Transcriptional changes specific for RH and IC

A total of 573 genes were differentially expressed in IC compared to RH, of which 238 were down‐ and 335 up‐regulated in IC (Figure 2c). Some 248 genes were infection and IC up‐regulated compared to RH, while only 44 were infection and RH up‐regulated genes (Table S5). The main gene families corresponding to infection up‐regulated genes in IC were SMC (38 genes, corresponding to 16 clusters), CAZymes (42), ROS (34), and PE (33) (Table 3). This comparison shows that IC expressed a specific set of putative virulence factors (CAZymes, PE, and SMC), which we further investigated.

**Table 3:** Infection up‐regulated genes differentially expressed in runner hyphae (RH) and infection cushions (IC) in gene families

Regulation	Non‐SP										SP										Non‐SP	SP	Total
	TF	TP	HM	PK	DH	CAZyme	PE	ROS	TMR	No	TF	TP	HM	PK	DH	CAZyme	PE	ROS	TMR	No	Total	Total	
RH up	1	2	0	0	0	2	0	6	0	23	0	0	0	0	0	1	3	4	0	5	34	13	47
IC up	4	14	3	3	10	3	0	23	4	88	0	0	0	0	1	39	33	11	0	21	152	105	257
Total	5	16	3	3	10	5	0	29	4	111	0	0	0	0	1	40	36	15	0	26	186	118	304

The colour‐coded heat map considers the total of infection up‐regulated genes regarding their regulation in infection cushions (IC) and runner hyphae (RH) from blue (highest) to red (lowest). Non‐SP, nonsecreted proteins; SP, putative secreted proteins; TF, transcription factors; TP, transporter proteins; HM, histone‐modifying proteins; PK, protein kinases/phosphatases; DH, dehydrogenases; CAZyme, carbohydrate‐active enzymes; PE, putative effector proteins; ROS, proteins related to reactive oxygen species; TMR, transmembrane receptors; no, genes without any annotation. Due to several genes belonging to more than one gene family, there is a higher total number of genes in this table than in the annotated genome (Data S1 and Figure 2b,c).

### 2.4 IC are enriched in plant cell wall‐degrading enzymes

**Figure 4:**
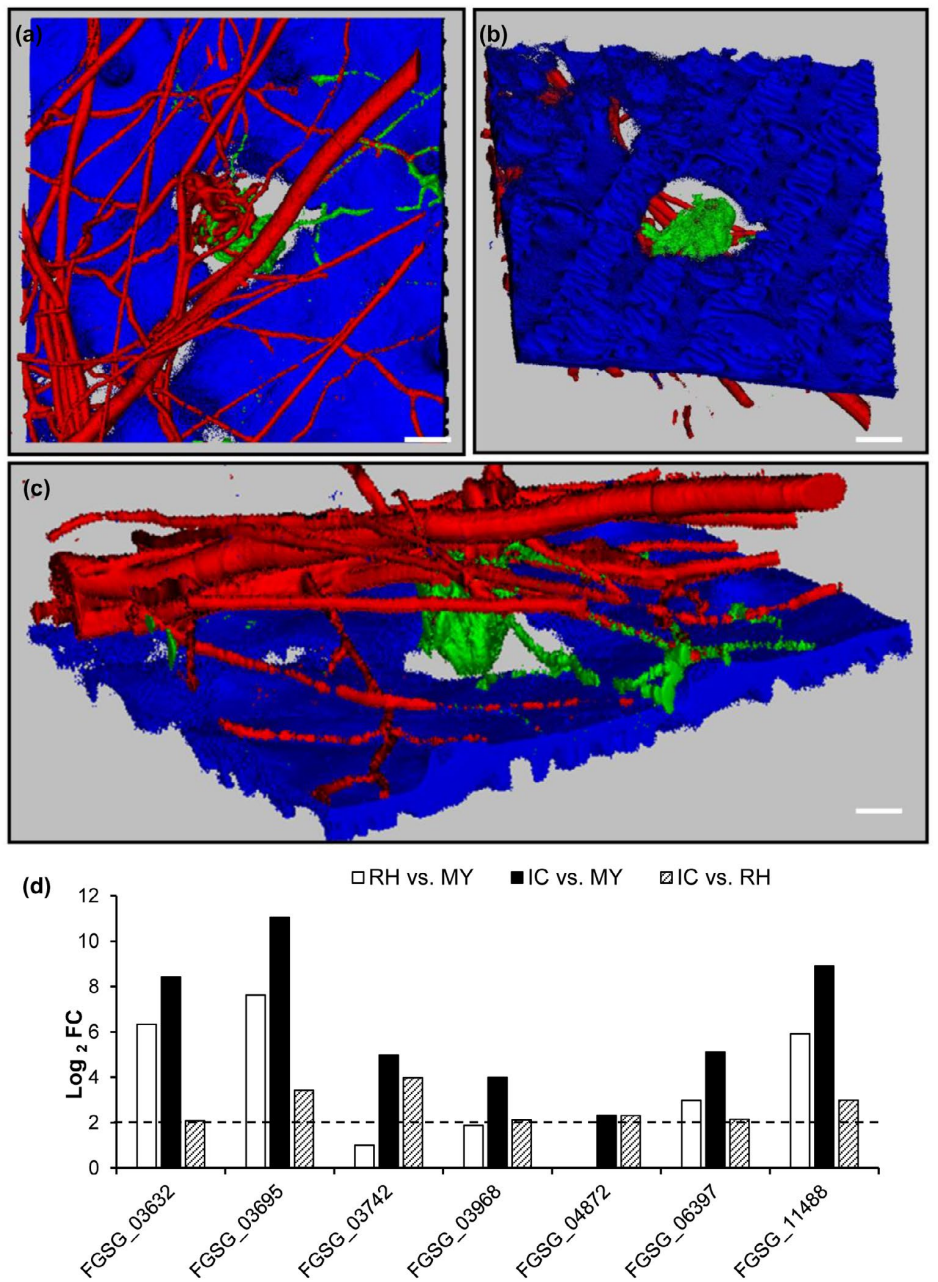
Plant cell wall alterations underneath infection cushions (IC) of *F* *usarium* *graminearum*. (a)–(c) Three‐dimensional reconstruction of confocal laser microscopic (LSM) images of a GFP‐fluorescent IC (green) and DsRed fluorescent runner hyphae (RH) (red) formed by the *TRI5_Prom_:GFP* with constitutive DsRed reporter strain on an inoculated palea (blue) at 6 days post‐inoculation recorded by an LSM *Z*‐ render series. (a) Top view of the sample shows a lack of blue plant autofluorescence underneath the IC. (b) The view from the bottom of the sample demonstrates more clearly that the fluorescence of the plant cell wall is reduced, especially underneath the IC. (c) Cut view of the sample. Scale bars: (a) and (b) = 10 µm, (c) = 5 μm. (d) Expression (log_2_ fold change [FC]) of the seven infection and IC up‐regulated CAZyme oxidoreductases. MY, mycelium grown in complete medium

CAZymes are proteins involved in cleavage, modification, or synthesis of glycosidic bonds (Lombard *et al*., 2014). The *F*.* graminearum* genome harbours 518 CAZyme encoding genes, of which 168 are involved in plant cell wall degradation (PCWDC), 95 in fungal cell wall modification (FCM), and 18 in starch biosynthesis/degradation (SDC). Genes involved in FCM corresponding essentially to chitin‐binding proteins, α‐mannan and β‐glucans biosynthesis or modification, and enzymes for starch/glycogen processing are enriched in the set of infection down‐regulated CAZymes (Table S6). Among the infection down‐regulated CAZyme genes, only six supposedly target the plant cell wall. On the contrary, 102 PCWDC are predominantly infection up‐regulated (Table S7). Among the 140 infection up‐regulated degradative CAZymes, 35 PCWDC and four FCM were specifically up‐regulated in IC, while only three genes (with yet unknown pathway annotation) were specifically up‐regulated in RH (Table S8). To test whether the production of PCWDC by IC has an impact on plant tissue, we assessed the status of the plant cell walls underneath IC by confocal laser microscopy. The intact plant epidermal cells showed a strong autofluorescence. Plant cells under and in the vicinity of IC completely lacked this autofluorescence (Figure 4a–c and Movie S1). Scanning electron microscopy showed that, in a later stage of infection in which fungal hyphae have already penetrated the plant underneath the IC, the cuticle is ruptured; however, an opening in the cell wall larger than the cushion itself was not observed (Figure 1c), as previously described (Bormann *et al*., 2014). Therefore, IC produce enzymes that mask or digest wheat cell wall compounds naturally emitting fluorescence after UV excitation like phenolic substances. Interestingly, 22 of the 61 redox CAZymes with proposed functions in degradation of phenolic compounds are infection up‐regulated (Data S3). In particular, seven of them are IC up‐regulated (Figure 4d).

### 2.5 IC are enriched in infection up‐regulated putative effectors

**Figure 5:**
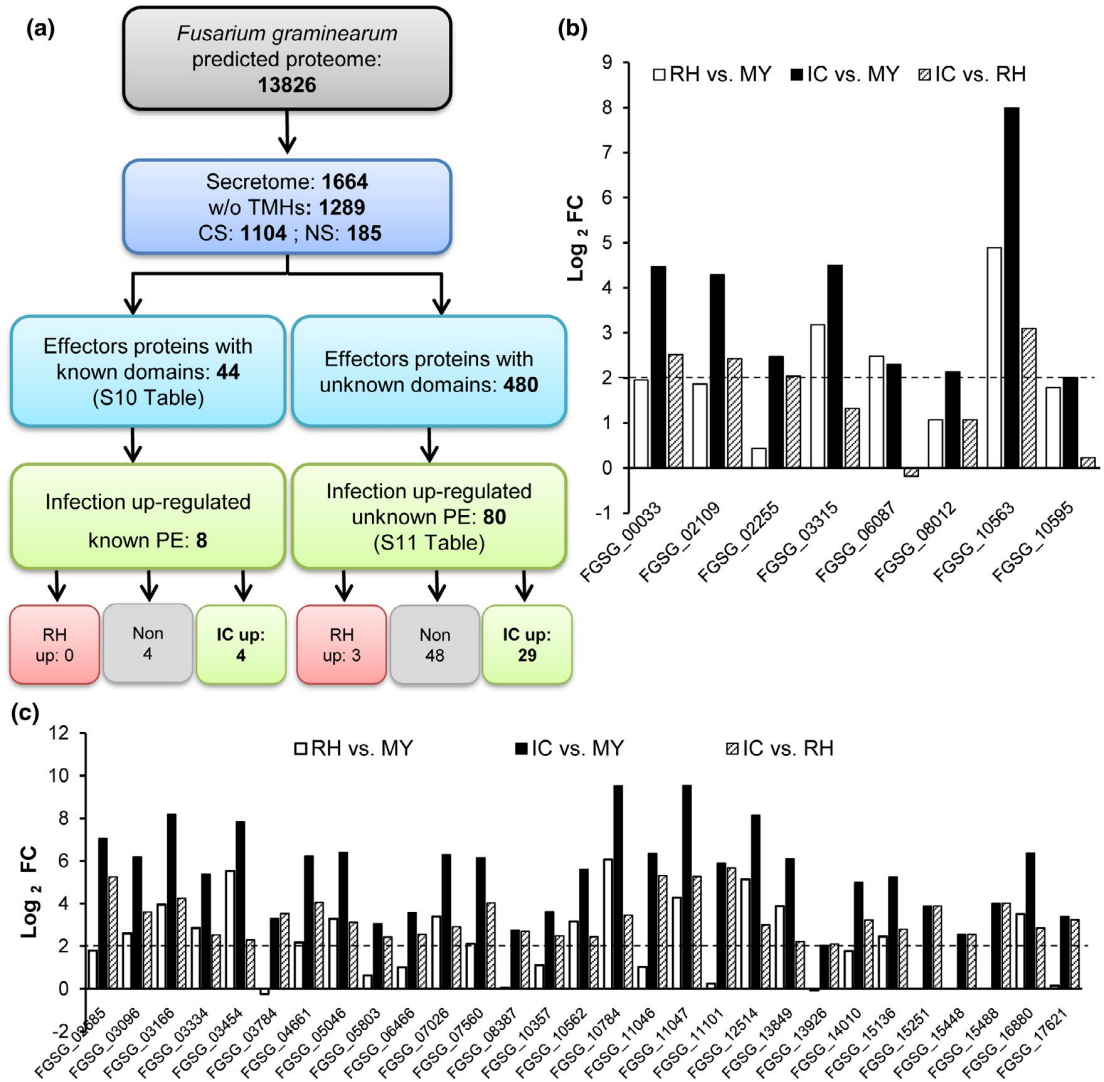
*F* *usarium* *graminearum* secretome prediction and effectors selection. (a) The secreted proteins were predicted using TargetP and SignalP for classic secretion (CS), or secretomeP and wolfPSORT for non‐classic secretion (NS). Proteins without transmembrane domains (w/o TMHs) were predicted using TMHMM. We identify putative effector proteins (PE) similar to effectors with known domains using IPRO or PFAM prediction domains (Table S10). Proteins with unknown domains, not belonging to a secondary metabolism gene cluster, with fewer than 1,000 amino acids, were defined as putative unknown effectors (Table S11). (b) Expression (log_2_ fold change [FC]) of the eight infection up‐regulated known PE. Four genes encode LysM proteins (FGSG_00033, FGSG_02255, FGSG_06087, and FGSG_10563), three for peptidase/proteinase inhibitor (FGSG_03315, FGSG_08012, and FGSG_10595), and one for CFEM protein (FGSG_02109). (c) Log_2_ FC of the 29 infection and IC up‐regulated unknown PE. Dashed lines show the log_2_‐threshold of +2 for up‐regulated genes. IC, infection cushions, RH, runner hyphae; MY, mycelium grown in complete medium

Secreted fungal effector proteins modulate host immune response to facilitate infection (Petre and Kamoun, 2014). Here, PE proteins were defined as secreted proteins, without transmembrane domains and a maximum size of 1,000 amino acids. Using this definition, 524 PE were identified (Data S4 and Figure 5a). Furthermore, 199 PE smaller than 200 amino acids and with a cysteine content higher than 2% were identified (Table S9). PE were classified as known effectors (PE: 44) containing previously identified domains and/or were defined as effectors in fungi or bacteria (Table S10), and as unknown effectors (PE: 480) with no predicted domains (Data S4 and Figure 5a). 77 PE were infection down‐regulated (10 known and 67 unknown), while 88 were infection up‐regulated genes (eight known and 80 unknown). Of the 88 infection up‐regulated PE, four knowns and 29 unknowns were specifically up‐regulated in IC compared to RH (Figure 5b,c). The 80 unknown infection up‐regulated PE were further classified according to their taxonomic specificity into 20 PE conserved across kingdoms and 60 fungal‐specific PE, including 14 *Fusarium*‐specific and 10 *F*.* graminearum*‐specific (Table S11).

### 2.6 Putative effector FgPE1 is up‐regulated in IC, localized at the interface between fungal and plant cell walls, and dispensable for pathogenicity

FgPE1 (FGSG_04213), a putative effector up‐regulated in IC compared to RH, was selected for further functional studies. FgPE1 gene expression was monitored by visualization of mCherry fluorescence driven by the FgPE1 promoter (FgPE1_Prom_::mCherry, Figure S3a). FgPE1 was expressed in both RH and IC on wheat palea, with the highest expression in IC at 4 days post‐inoculation (dpi) (Figure S3b,c). A cross‐section of an IC demonstrates high level of expression of FgPE1 (Figure S3d,e) during tissue invasion. Interestingly, also mycelia grown in wheat medium displayed strong FgPE1 expression at 1 and 3 dpi (Figure S3f,g). Mycelia grown in CM, in contrast, exhibited only faint mCherry signals at 1 dpi, slowly ramping up until 3 dpi (Figure S3h,i), indicating time‐ and nutrient‐dependent regulation.

To determine the subcellular localization, FgPE1 was translationally fused to mCherry (FgPE1_Prom_::FgPE1::mCherry, Figure S4a) and transformed into a wild‐type‐ (WT) strain expressing cytoplasmic green fluorescent protein (GFP) constitutively. A high level of FgPE1‐mCherry was found in conidia produced on wheat medium (Figure S4b,c). In addition, FgPE1‐mCherry localized around old hyphae but not in young growing hyphae, both on palea (6 dpi; Figure S4f–h) and in wheat medium (1 and 3 dpi; Figure S4i–l). On CM, however, no FgPE1 signal was observed at 1 and 3 days of growth (Figure S4m–p), suggesting that FgPE1 expression is induced in the presence of plant‐derived compounds. To determine whether FgPE1 was localized at the fungal cell wall or plasma membrane, protoplasts were generated and incubated on wheat medium for 20 hr. Confocal laser microscopy of undigested mycelia and partially digested hyphae showed that the mCherry signal was located around hyphae (Figure S5a–f) while protoplasts lacked a mCherry signal, demonstrating that FgPE1 localizes to the fungal cell wall and not to the plasma membrane (Figure S5).

**Figure 6:**
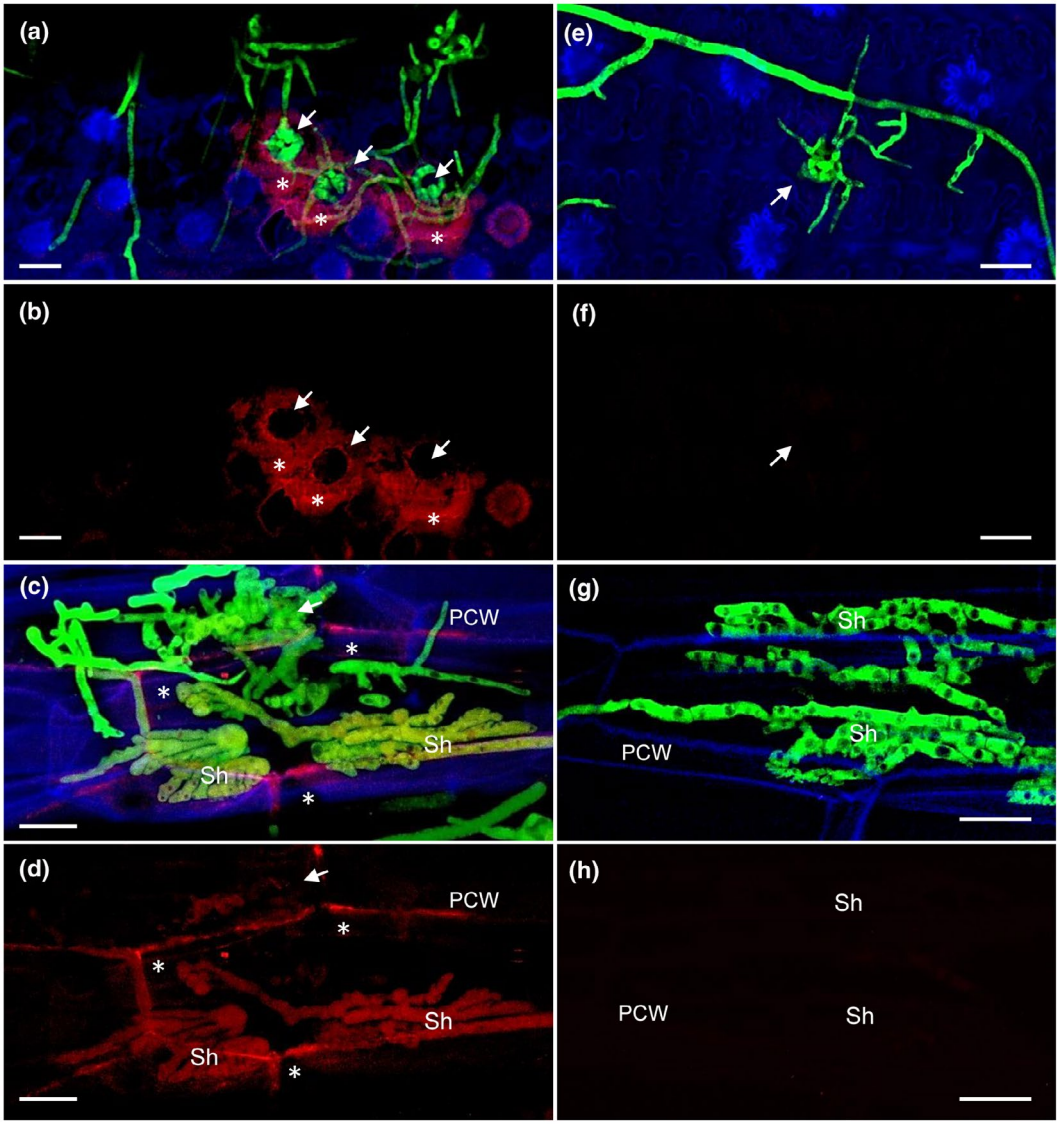
FgPE1 is secreted and mainly localized at the fungal–plant interface. Wheat paleae were inoculated with the mutant carrying the construct FgPE1_Prom_::FgPE1::mCherry and constitutive green fluorescent protein (GFP) (a)–(d) or the wild‐type (WT)‐GFP strain (e)–(h). The FgPE1 protein is secreted (4 days post‐inoculation, dpi) and accumulates around infection structures (a) and (b). At 6 dpi a distinctive vacuolated subcuticular hyphae (Sh) beneath infection structures produces the putative effector FgPE1, accumulating it at the fungal–plant interface. PCW, plant cell wall (c) and (d). The WT‐GFP strain does not show an mCherry background signal under similar conditions (e)–(h). Pictures were taken with a confocal microscope (CLSM‐Zeiss) with maximum intensity projections of 25–35 pictures. Overlay image of photographs taken with DAPI, mCherry, or GFP filters individually and combined were prepared using Zeiss AxioVision software. White arrows indicate the localization of infection cushions (IC). FgPE1 protein is indicated with white stars. Blue, plant autofluorescence; green, GFP‐constitutive; red, FgPE1::mCherry. Scale bar = 20 µm

When inoculated on paleae, mCherry signals were first observed around IC (Figure 6a,b). Six days after inoculation, vacuolated subcuticular hyphae were produced beneath IC that displayed a strong mCherry signal (Figure 6c,d and Movie S2). In addition, a mCherry signal was present in the fungal–plant cell wall interface in the vicinity of fungal infection hyphae (Figure 6c,d and Movie S2). The WT strain expressing cytosolic GFP was used as control and lacked a fluorescence signal in the detection range of mCherry, proving that the FgPE‐mCherry signal is specific and not due to plant‐derived autofluorescence (Figure 6e–h).

FgPE1 was deleted by gene replacement (Figure S6a,b). In virulence assays on wheat the deletion mutants as well as an ectopic mutant and WT exhibited full infection, indicating that FgPE1 is dispensable for virulence (Figure S6c,d).

### 2.7 Wheat infection triggers secondary metabolite production

**Figure 7:**
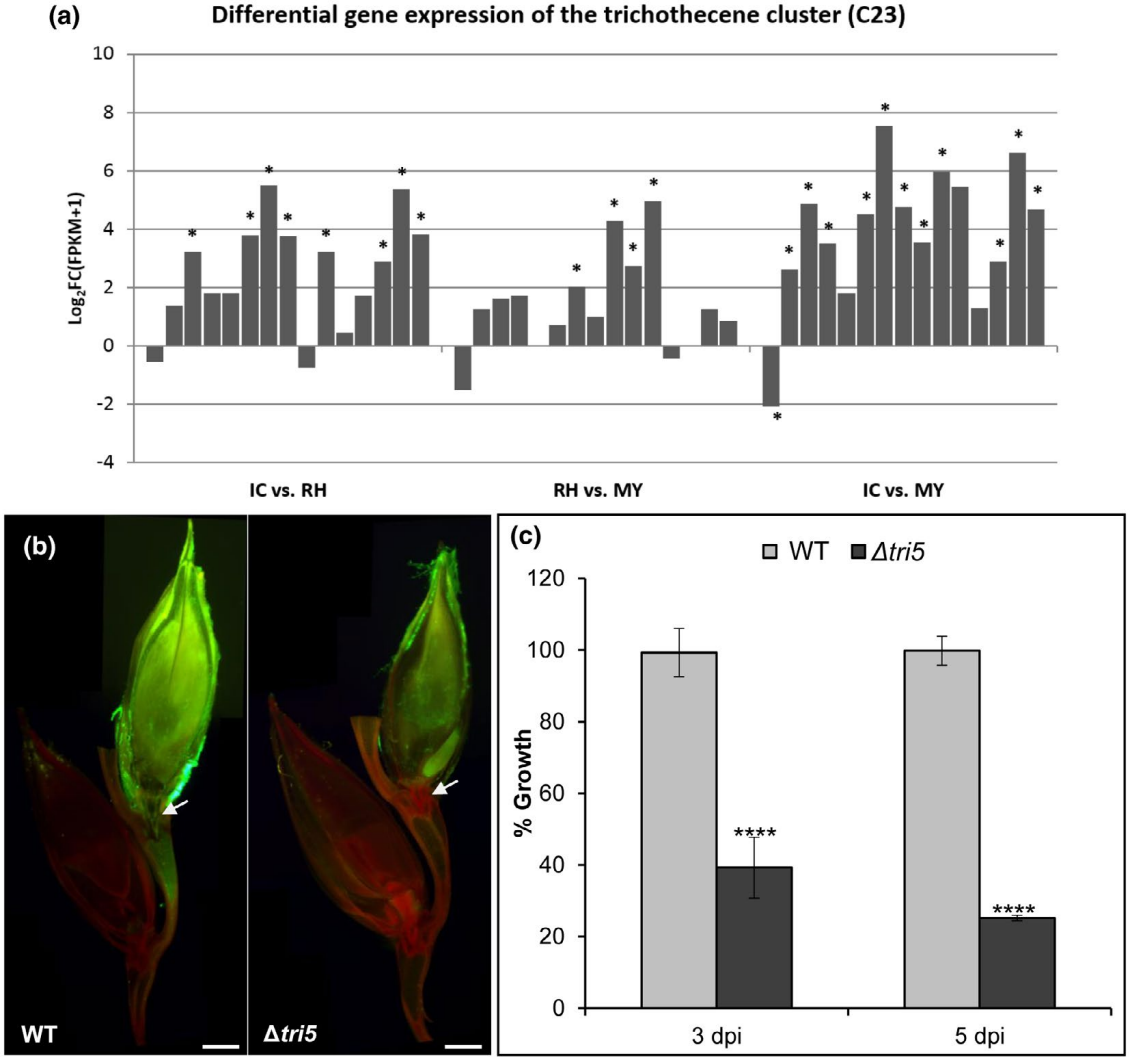
Deoxynivalenol (DON) production by the trichothecene gene cluster C23 is necessary for fungal growth at early time points of wheat infection. (a) The expression of the trichothecene gene cluster C23 (from left to right: FGSG_03529 – FGSG_03535, FGSG_03537 – FGSG_03543, and FGSG_16251) is strongly up‐regulated in infection cushions (IC) compared to runner hyphae (RH). C23 cluster also shows a significant up‐regulation in IC and RH compared to mycelium grown in complete medium (MY). Asterisks indicate significant up‐regulation for log_2_ FC(FPKM + 1) ≥ 2 or down‐regulation log_2_ FC(FPKM + 1) ≤ −2. (b) Cross‐section images show wild‐type (WT)‐green flourescent protein (GFP) mycelia copiously grown in inoculated spikelet and across the rachis node, while Δ*tri5*‐GFP mutant grows scarcely and is unable to cross the rachis node. Images are a composition of three pictures taken at 5 days post‐inoculation (dpi). White arrows indicate the rachis node. Scale bar = 2 mm. (c) Δ*tri5*‐GFP fungal growth is 60% and 75% less than the WT‐GFP at 3 and 5 dpi, respectively. Error bars indicate ±*SD* calculated from data of three independent experiments and three experimental replicates (*n* = 9). Significance with respect to WT, ****p < .0001 (calculated with ANOVA‐Bonferroni‐Holm)

In this study, 53 of 67 SMC (Sieber *et al*., 2014) were expressed in any of the tested conditions (Table 2). The remaining 14 SMC, including those involved in biosynthesis of zearalenone (C15), butenolide (C49), fusarin C (C42), and fusarielin (C60), were neither expressed in CM nor during infection (Table 2). Twenty‐three SMC were infection down‐regulated, among them the carotenoid (C28) and siderophore ferricrocin clusters (C33, Table 2). Interestingly, there was one unknown SMC (C09) specifically down‐regulated in IC, while similarly expressed in RH and MY. Two undescribed SMC (C37 and C40) were specifically down‐regulated in RH and similarly expressed in IC and MY (Table 2). Twelve SMC were infection up‐regulated. Among the six SMC up‐regulated in RH and IC were two known siderophore clusters, triacyl fusarin (C21) and malonichrome (C63). Five SMC were specifically up‐regulated in IC, including the known virulence factors trichothecene (TRI, C23, Figure 7a) and fusaoctaxin A (C64, Table 2). To determine the impact of DON on the initial plant colonization, we quantified and compared spikelet infection of the DON‐deficient trichodiene synthase deletion mutant (∆*tri5*) and the WT strain, both expressing GFP constitutively (Jansen *et al*., 2005). Fluorescence microscopy of longitudinal cuts through inoculated wheat spikes revealed less mycelial growth inside spikelets inoculated with the ∆*tri5* mutant compared to the ones inoculated with the WT (Figure 7b). For molecular quantification, fungal DNA from inoculated spikelets was extracted and the relative amount measured by quantitative PCR (qPCR) as previously described (Voigt *et al*., 2007). Results revealed that ∆*tri5* grew 60% and 75% less than the WT at 3 and 5 dpi, respectively (Figure 7c). Hence, DON facilitates rapid colonization of plant tissues at the initial stage of infection.

### 2.8 Aurofusarin is a RH‐specific antibiotic active against a wide range of microorganisms

**Figure 8:**
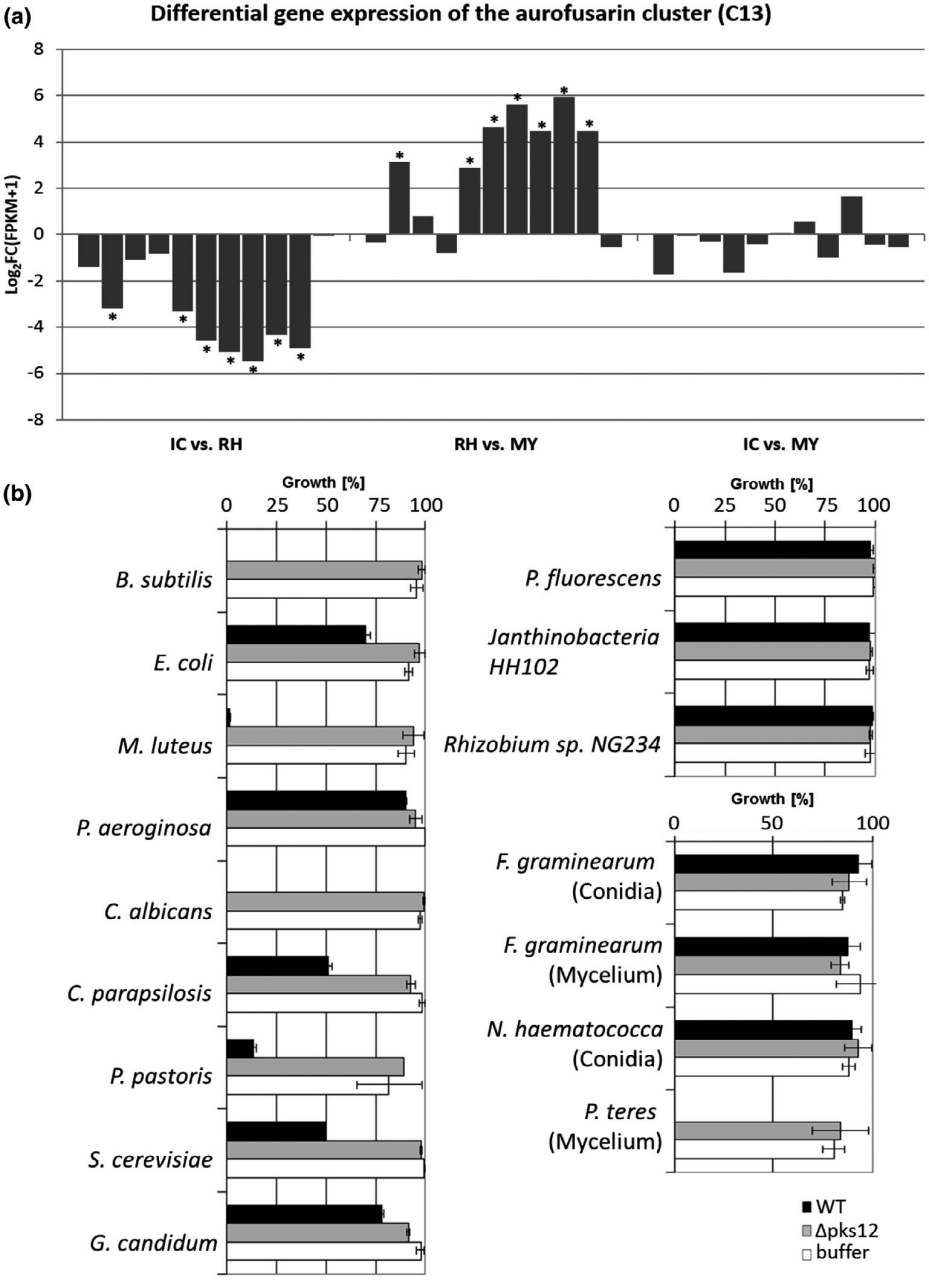
The aurofusarin cluster C13 is up‐regulated in runner hyphae (RH) and responsible for toxicity to bacteria and fungi. (a) The aurofusarin gene cluster C13 (from left to right: FGSG_02320 – FGSG_02330) is specifically up‐regulated in RH compared to infection cushions (IC) or mycelium grown in complete medium (MY). No significant difference in gene expression of C13 cluster was found between IC and MY. Differential expression of genes is given by log_2_ FC(FPKM + 1) values. Asterisks indicate significant up‐regulation for log_2_ FC(FPKM + 1) ≥ 2 or down‐regulation log_2_ FC(FPKM + 1) ≤ −2. (b) Bioactivity assay using extracts of wild‐type (WT) and aurofusarin‐deficient mutant ∆*pks12*. For bacteria and yeasts, OD_595_ was measured after 16 hr of growth in medium supplemented with either WT extracts, extracts of the aurofusarin‐deficient ∆*pks12* mutant, or phosphate buffer as a control. For filamentous fungi, dry weight was calculated. The highest value of buffer controls was set to 100% of growth. For every condition and organisms tested, three biological replicates were performed

The single SMC specifically up‐regulated in RH is involved in aurofusarin biosynthesis (C13) and is strongly down‐regulated in IC (Table 2 and Figure 8a). To test whether aurofusarin could act as an antibiotic against microbial competitors growing on the wheat floral tissue's surface, the toxicity of aurofusarin on bacteria and fungi was assessed using mycelium extracts from either WT or the aurofusarin‐deficient Δ*pks12* mutant (Figure 8b and Table S12). The presence or absence of aurofusarin in extracts was determined by LC‐MS (Figure S7). WT extract was highly toxic to the gram‐positive bacteria *Bacillus subtilis* and *Micrococcus luteus* (98%–100% growth inhibition [GI]), while other bacteria such as *Escherichia coli*, *Pseudomonas aeruginosa*, *Pseudomonas fluorescens*, *Janthinobacteria* HH102, and *Rhizobium* sp. NG234 were insensitive (Figure 8b). WT extract was also highly toxic to *Pyrenophora teres* (100% GI), *Candida albicans* (100% GI), and *Pichia pastoris* (85% GI), while moderately toxic to *Candida parapsilosis* (50% GI) and *Saccharomyces cerevisiae* (50% GI). *F*.* graminearum* and, closely related, *Nectria haematococca* were totally insensitive to WT extracts (Figure 8b). Extracts from Δ*pks12* were completely nontoxic to all tested organisms, demonstrating that aurofusarin is indeed responsible for the observed toxicity of WT extract to bacteria and fungi.

## 3 DISCUSSION

Although FHB is a devastating cereals disease that occurs worldwide, the molecular basis of the initial steps of infection are basically unknown. On the contrary, the large‐scale reprogramming of appressorial gene expression of the plant pathogens *M*.* oryzae* and *Colletotrichum* species is well known (O’Connell *et al*., 2012; Soanes *et al*., 2012). Here, the transcriptomes of hyphae grown in culture (MY) and epiphytically grown RH revealed fundamental transcriptional differences. Interestingly, the majority of these genes (73% out of 2,390 genes) are down‐regulated in RH compared to MY. These transcriptional changes highlight the huge differences between fungal hyphae growing in a nutritious broth and under very restrictive conditions on palea. Similar results were reported from the plant pathogen *Colletotrichum higginsianum* where transcriptomes of appressoria built in culture and on leaves were significantly different, though morphologically indistinguishable (O’Connell *et al*., 2012), stressing the necessity to elucidate the different aspects of fungal development in its natural environment.

The majority of up‐regulated genes in RH or IC compared to MY encode hypothetical proteins with unknown function, indicating a requirement for characterization of such proteins (Tables S2 and S3). The next more up‐regulated genes encode for CAZyme degradative enzymes, suggesting preparation to break the plant cell wall (Tables S2 and S3). Thirty‐five plant cell wall‐degrading enzymes (PCWDC) are specifically induced in IC encoding enzymes with predicted cellulolytic, xylanolytic, pectinolytic, and oxidoreductase activity. The activity of these PCWDC may cause the observed loss of blue fluorescence around the developed IC, preceding penetration. This fluorescence of the plant epidermal cell wall is attributed especially to phenolic substances such as chlorogenic acid, caffeic acid, coumarins, stilbenes, and ferulic acid of the stress‐induced phenylpropanoid pathway, which are also known to be involved in plant defence (Dixon and Paiva, 1995; Lang *et al*., 1991). Cell wall‐bound ferulic acid is the major substance causing blue light emission in grasses like wheat (Lichtenthaler and Schweiger, 1998). Interestingly, ferulic acid inhibits in‐culture DON biosynthesis at the transcriptional level (Boutigny *et al*., 2009). DON, in turn, does not contribute to the loss of fluorescence, as a DON‐deficient mutant causes the same phenotypes as the wild type (Boenisch and Schäfer, 2011).

Among the five secondary metabolite clusters specifically induced in IC are deoxynivalenol (C23, DON) and fusaoctaxin A (C64), both having important roles during infection (Table 2). Fusaoctaxin A facilitates the cell‐to‐cell movement of the fungus and is important for virulence during coleoptile as well as wheat spike infection (Jia *et al*., 2019; C64 is FG3_54 in Zhang *et al*., 2012). DON inhibits the eukaryotic translational machinery and is essential for colonization of the spike, with massive induction during colonization of the developing caryopses and the rachis node (Ilgen *et al*., 2009). Importantly, both SMC are up‐regulated in IC clearly in preparation for the following colonization steps. DON‐deficient mutants fail to cross the rachis node (Proctor *et al*., 1995; Maier *et al*., 2006; Ilgen *et al*., 2009), which is accompanied by plant cell wall thickening and jasmonate‐related defence reactions preventing further fungal colonization (Jansen *et al*., 2005; Bönnighausen *et al*., 2019). Our results verify previous fluorescence‐microscopy assisted analyses of a specific IC induction of *tri5* (FGSG_03537). Yet, this specific induction and subsequent DON biosynthesis are neither necessary for IC development nor plant cell wall penetration (Boenisch and Schäfer, 2011). Quantification of fungal DNA in infected spikelets now revealed a substantially slower spikelet infection of the DON deficient Δ*tri5* mutant compared to WT‐GFP strain. DON, therefore, acts as a virulence factor immediately after penetration. DON was shown to induce programmed cell death (PCD) after infiltration into plant tissues (Desmond *et al*., 2008; Diamond *et al*., 2013; Blümke *et al*., 2015). Therefore, DON‐induced PCD may facilitate the release of nutrients during initial infection. Loss of DON, in turn, may result in less nutrients available, causing the observed growth reduction at 3 and 5 dpi. DON deficiency may, therefore, enable successful plant defence reactions that are, in the case of the WT, not initiated or suppressed.

The cluster for aurofusarin biosynthesis is the only one specifically induced in RH. Aurofusarin is a red pigment produced by different *Fusarium* species, belonging to polyphenol, more accurately *bis*‐naphthopyrone pigments (Frandsen *et al*., 2006; Xu *et al*., 2019). In a previous study, deletion of the *F*.* graminearum pks12* gene (FGSG_02324) led to a loss of red pigment, a higher growth rate, and 10‐fold more conidia production than WT but had no impact on pathogenicity on wheat and barley (Malz *et al*., 2005). Recently, aurofusarin has been described to inhibit *Lactobacillus* and *Bifidobacterium*, but not *E*.* coli* (Sondergaard *et al*., 2016). Excitingly, it has been described as an antifeedant that accumulates in high amounts to protect *Fusarium* fungi from a wide range of insects (Xu *et al*., 2019). The microbiology of the phyllosphere is, in general, not very well understood, but it seems safe to assume that RH of *F*.* graminearum* ward off other microbes during colonization of the palea's surface. Among the bacteria found in the microbiome of wheat spikes are *Pseudomonas*, *Bacillus*, *Janthinobacterium*, and *Actinomycetes* (Chen *et al*., 2018). In this study, we showed that aurofusarin is an inhibitor of different bacterial and fungal species, among them yeast, including most notably the widespread human pathogen *C*.* albicans*. Another polyphenol pigment found in *F*.* graminearum* is bostrycoidin purpurfusarin, which is also known to have antibiotic properties against *C*.* albicans* (Frandsen *et al*., 2016). Further research will show if these secondary metabolites could improve the fight against this widespread human pathogen.

Up‐regulated in RH and IC are two infection up‐regulated iron‐chelating siderophores, triacetyl fusarin and malonichrome, which are necessary for virulence (Oide *et al*., 2015). A third infection up‐regulated iron‐chelating siderophore, ferricrocin, is important for sexual development but not for virulence (Oide *et al*., 2015), explaining why we found this metabolite down‐regulated during infection. Fourteen SMCs were not at all expressed, including zearalenone (C15), fusarin C (C42), butenolide (C49), and fusarielin (C60) clusters, all dispensable for wheat infection (Gaffoor *et al*., 2005; Harris *et al*., 2007; Sørensen *et al*., 2012). *F*.* graminearum* is a pathogen with a variety of hosts such as wheat, barley, oats, rye, maize, and soybean (Savary *et al*., 2012; Sella *et al*., 2014), and many transcripts not detected might be necessary for specific colonization of such hosts (Harris *et al*., 2016). For instance, the SMC DON necessary for wheat infection, and highly transcribed under our study conditions, does not seem to act as a virulence factor on barley (Maier *et al*., 2006). Therefore, the lack of transcript detection could be due to specificity or redundancy on their protein function.

Besides CAZymes and SMC, we identified a large number of putative effector proteins (PE) up‐regulated in IC. Previously, Lu and Edwards (2016) identified 190 small (≤200 amino acids) and cysteine‐rich (≥2%) secreted proteins as candidate effectors in *F*.* graminearum*. Here, we identified 199 proteins with such characteristics (Table S9). However, several studies reported effectors as secreted proteins with very diverse sizes or cysteine content (Kulkarni *et al*., 2003; Rooney *et al*., 2005; Djamei *et al*., 2011; Frías *et al*., 2011; Sperschneider *et al*., 2013; Blümke *et al*., 2014; Tanaka *et al*., 2014; Jashni *et al*., 2015; Quarantin *et al*., 2016). From the 524 identified PEs, 88 were infection up‐regulated and of these, 33 were specifically up‐regulated in IC. Four PEs specifically up‐regulated in IC contained LysM or CFEM domains, being indicative of key proteins necessary during penetration of the host cell by suppressing fungal recognition and manipulating host functions (Mentlak *et al*., 2012; Zhang *et al*., 2012; Takahara *et al*., 2016). The hemibiotroph *C*.* higginsianum* transcribes effectors in consecutive waves associated with the transitions in the pathogen's lifestyle (Kleemann *et al*., 2012). Hence, it is likely that *F*.* graminearum* expresses a different set of effectors during later stages of plant colonization. The study of the 80 unknown plant‐induced fungal effector‐like proteins (Table S11) could lead to the discovery of new targets for fungal control and even specifically *F*.* graminearum* control. In a different approach using a comparative genome analysis, a set of 2,830 *F*.* graminearum* genes, presumably associated with pathogenicity, were identified (Sperschneider *et al*., 2013). We found that roughly 3.9% of these genes (111) are transcriptionally up‐regulated during palea colonization, with 35 of them specifically up‐regulated in IC and 30 encoding PEs. This set of PEs with high specificity for cereal infection could be of outstanding importance for the initial host–pathogen interaction.

The highly expressed in IC fungal PE, called FgPE1, was characterized. *FgPE1* encodes a secreted 151 amino acid protein with an Alt‐A1 allergen analog domain present in the Alt‐A1 effector from *Alternaria alternata* (Chruszcz *et al*., 2012). Recently, an Aa1‐like protein, PevD1 from *Verticillium dahliae*, has been found to interact and inhibit the antifungal activity of GhPR5 cotton plant protein as a strategy to fight the plant defence and promote fungal infection (Zhang *et al*., 2018). Here, expression results indicated a transcriptional regulation of *FgPE*1 depending on plant factors. A previous report showed that *FgPE1* is highly expressed at different time points (4, 12, 24, 48, and 72 hpi, and 8 and 14 dpi) during infection of wheat spikes and at 8 days in old minimal medium culture (Lu and Edwards, 2016), supporting the hypothesis that *FgPE1* expression is regulated by plant factors and nutrient availability and that it is present not only during early infection but all along the infection process. Replacement of *FgPE1* in *F*.* graminearum* did not affect virulence according to our infection assays (Figure S6). We assume a high degree of functional redundancy within the PE gene family. This is in accordance with studies, for example in *U*.* maydis*, that, for the most part, failed to identify novel, virulence‐specific genes, for example within a pool of potential effector genes (Kämper *et al*., 2006). FgPE1 is localized at the fungal cell wall. During IC formation, FgPE1 is secreted and at first localized at plant cell walls in the close vicinity around the IC. Once the fungus grows beneath the plant surface, FgPE1 is localized at the fungal–host interface. Effectors with such localization such as ChEC34 and ChEC89 of *C*.* higginsianum* (Kleemann *et al*., 2012) or the CFEM1 protein (FGSG_02077) from *F*.* graminearum* (Zhang *et al*., 2012) usually are necessary for eliciting or suppressing the plant recognition depending on their lifestyle.

Taken together, we found major transcriptional changes between epiphytical and in‐culture hyphae. A complex set of virulence‐associated factors, comprising plant cell wall‐degrading enzymes, secondary metabolites, and effector proteins among others, are synthesized in IC, preparing the fungal hyphae for successful penetration and subsequent colonization of plant tissue. Therefore, IC are arsenals of fungal combat and the genes expressed in them could provide potentially novel targets for *Fusarium* control.

## 4 EXPERIMENTAL PROCEDURES

Detailed experimental procedures are described in Methods S1.

### 4.1 Fungal growth and conidia production

*F*.* graminearum* wild type (WT; Fg‐8/1; Miedaner *et al*., 2000) and mutants used and produced in this study were grown, cultured, and transformed as described before (Jansen *et al*., 2005).

### 4.2 Preparation of wheat‐infected tissue for laser capture microdissection

Detached wheat palea infection assay was prepared according to Boenisch and Schäfer (2011). Mycelium samples were prepared by inoculating 750 conidia of the WT‐GFP strain in 50 ml CM and incubated for 3 days. A mycelium piece of about 1 mm in diameter was used for RNA extraction, amplification, and construction of cDNA libraries.

### 4.3 Laser capture microdissection

Paleae containing RH and IC were prepared by cutting off their upper and lower ends and immediately transferred to absolute ethanol on ice according to previous studies (Goldsworthy *et al*., 1999; Clément‐Ziza *et al*., 2008). RH and IC were prepared and dissected as mentioned in Methods S1.

### 4.4 RNA extraction, amplification, and cDNA library construction

RNA extraction, amplification, and cDNA library construction of IC, RH, or MY were performed according to Lê *et al*. (2005). See Methods S1.

### 4.5 Purification of cDNA libraries

For the removal of primers, enzymes, and other substances of the process from the cDNA libraries the NucleoSpin Gel and PCR Clean‐up Kit (Macherey‐Nagel) was used according to the manufacturer's instructions.

### 4.6 Finalization of cDNA libraries (end‐it reaction)

To provide 5′‐phosphorylated, blunt‐ended cDNAs, the End‐It DNA End‐Repair Kit (Biozym Biotech Trading GmbH) was used according to a modified protocol. One microgram of the final cDNA libraries of three independent replicates of mycelia, RH, and compound appressoria, respectively, were sent for RNA‐Seq analysis. See Methods S1.

### 4.7 RNA‐Seq mapping and quantification

RNA‐Seq reads were mapped on the reference genome using tophat2 v. 2.0.8. The interval for allowed intron lengths was set to minimum 20 nt and maximum 1 kb (Trapnell *et al*., 2009). Three highly correlating replicates were used according to the Pearson correlation test (Table S1). We used cufflinks to determine the abundance of transcripts in FPKM (fragments per kilobase of exon per million fragments mapped) and calculated differentially expressed genes using cuffdiff (Trapnell *et al*., 2009; Trapnell *et al*., 2012). The gene models were included as raw junctions. The uncorrected *p* value and the FDR‐adjusted *p* value of the test statistic (*q* value) were calculated, *p* and *q* values per each gene are given in the general part of Data S1. Any given gene of interest can be evaluated by its fold change of transcription and by the resulting *p* and *q* values. Genes with a minimum of four‐fold increase or decrease in expression (|log_2_ of the FPKM values + 1| ≥ 2) between two experimental conditions were considered as regulated.

### 4.8 Annotations and databases used

The transcriptome data discussed in this publication have been deposited in NCBI’s Sequence Read Archive (SRA; https://www.ncbi.nlm.nih.gov/sra/, SUB3191581; Edgar *et al*., 2002). The reference genome of *F*.* graminearum* PH1 database FGDB (ftp://ftpmips.gsf.de/fungi/Fusarium/F_graminearum_PH1_v32/) was used to map the cDNA libraries constructed (Wong *et al*., 2010). Genes were manually grouped in gene families using the tool at https://ghr.nlm.nih.gov/primer/genefamily/genefamilies. See Methods S1.

### 4.9 Validation of RNA‐Seq data by qPCR

Validated genes were FgTRI5 (FGSG_03537), FgPKS12 (polyketide synthase 12; FGSG_02324), FgPE1 (FGSG_04213; putative effector1), and two GABA‐aminotransferases (FgGTA1, FGSG_05554; FgGTA2, FGSG_06751). For relative expression analysis the tool REST (Relative Expression Software Tool) was used (Pfaffl *et al*., 2002). For evaluation of the housekeeping genes cofilin (FGSG_06245) and ubiquitin (FGSG_10805) we used the comprehensive tool “Ref Finder” (Tables S13 and S14; Vandesompele *et al*., 2002; Andersen *et al*., 2004; Pfaffl *et al*., 2004; Silver *et al*., 2006). For details see Methods S1.

### 4.10 Generation of knock‐out, expression, and localization constructs for FgPE1 mutants

All plasmids were constructed using the yeast recombination method (Colot *et al*., 2006) and the pRS426 background plasmid (Christianson *et al*., 1992). Amplification of the ORF, and 5′ and 3′ flanks of the genes of interest was performed using primers shown in Table S15 and genomic DNA extracted from the WT strain. The final constructs were excised with the respective restriction enzymes (Table S16) and used to transform *F*.* graminearum* WT or WT‐GFP strains. At least two independent mutants were generated and examined. For details see Methods S1.

### 4.11 Virulence assay: wheat spikes point inoculation and wheat palea infection

Virulence assays where prepared according to Boenisch and Schäfer (2011) and Frandsen *et al*. (2006). For details see Methods S1.

### 4.12 Quantification of fungal material within inoculated wheat spikes using qPCR

Genomic DNA of inoculated wheat spikes was isolated using the CTAB method and according to Voigt *et al*. (2007). For details see Methods S1.

### 4.13 Fluorescence microscopy

Histological studies of WT‐GFP, ∆*tri5‐*GFP, and mutants generated in this study were performed as previously described in Boenisch and Schäfer (2011). For details see Methods S1.

### 4.14 Scanning electron microscopy

Scanning electron microscopy (SEM) was done with SEM LEO 1525 at 6 kV using detached palea of wheat cultivar Nandu inoculated with 5 µl of 2 × 10^4^ conidial suspension of WT‐GFP strain and prepared as described (Boenisch and Schäfer, 2011). To identify penetration pores, infection structures were removed from the plant surface of critical point dried paleae using adhesive tape and processed for SEM as previously described (Bormann *et al*., 2014).

### 4.15 Extraction of aurofusarin from *F*.* graminearum* WT and aurofusarin‐deficient mutant Δ*pks12*

Fungal material of the WT strain and the aurofusarin‐deficient mutant was harvested after 4 days from 50 ml CM liquid cultures. The respective mycelium was harvested using Miracloth, washed with 100 ml double‐distilled water (ddH_2_O) and semi‐dried using a filter paper. Around 1 g of mycelium was transferred into a 2 ml tube and supplemented with 1 ml potassium phosphate‐buffer (50 mM, pH 7). After addition of two metal pearls (3 mm diameter), the solution was ground for 15 min using a Retsch mill. After centrifugation at 13,000 rpm for 15 min, the extracted supernatant was filter sterilized using a 0.22 µm Millex GP filter.

### 4.16 Analysis of fungal extracts via LC‐MS

The extracts of WT strain and Δ*pks12* mutant were analysed as described in Methods S1.

### 4.17 Bioactivity assay

Liquid cultures of the organism listed in Table S12 were used for bioactivity assays. Assays were performed as described in Methods S1.

## Data Availability

The data produced in this publication have been deposited in NCBI’s Sequence Read Archive (SRA), https://www.ncbi.nlm.nih.gov/sra/; accession SUB3191581.
