# Supplementary material for: Infection cushions of Fusarium graminearum are fungal arsenals for wheat infection
Source: Mol Plant Pathol. 2020 Jun 23;21(8):1070–87. doi: 10.1111/mpp.12960 (PMC7368127; doi:10.1111/mpp.12960)
Supplement: Supplementary file 1 [file MPP-21-1070-s001.docx]

**
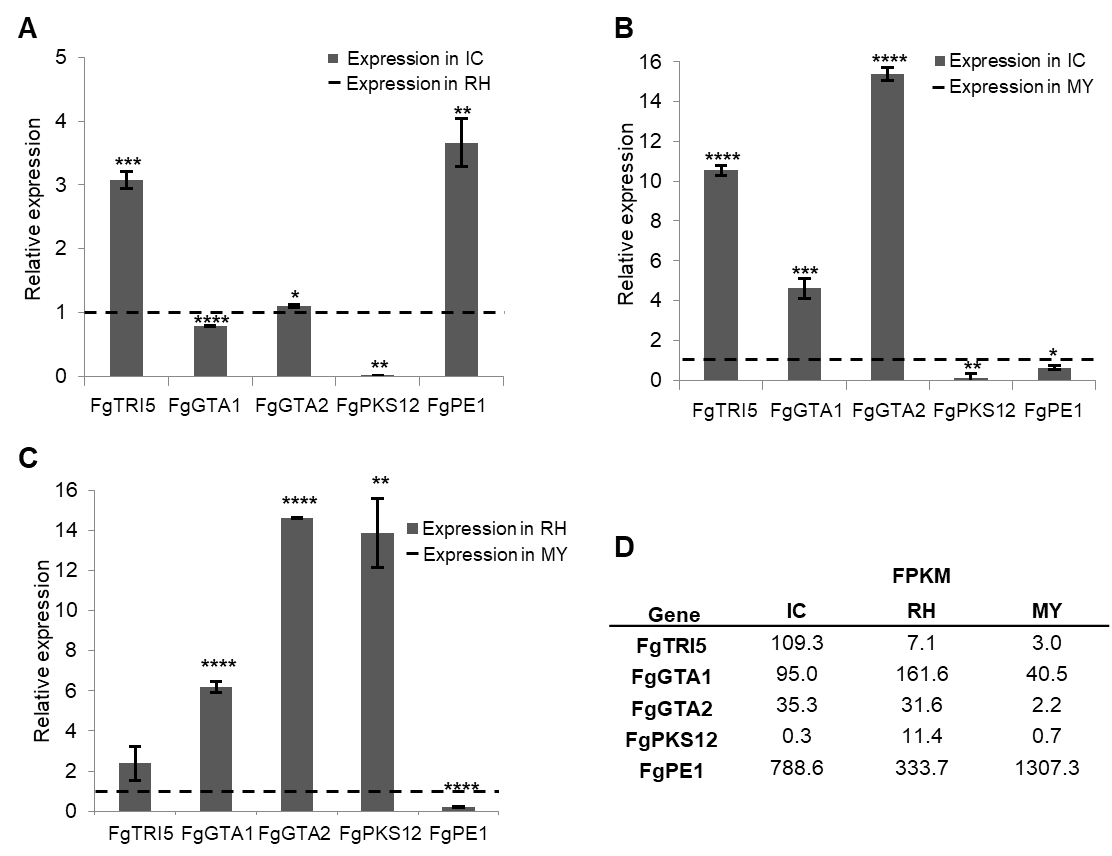
**

**Fig S1. Validation analysis of differentially expressed genes using RT-qPCR.** To validate the transcriptome data, the relative expression of FgTRI5 (trichodiene synthase, FGSG_03537), FgGTA1 (GABA-transaminase 1; FGSG_05554), FgGTA2 (FGSG_06751), FgPKS12 (polyketide synthase 12, FGSG_02324) and FgPE1 (putative effector1, FGSG_04213) in IC and RH was measured and compared to their expression in either RH or MY. (**A-C**) Relative expression of candidate genes in IC compared to RH (**A**) or MY (**B**) and in RH compared to MY (**C**). Error bars indicate standard deviations calculated from at least duplicates and are representative of three biological replicates. Significance with respect to either RH or mycelia: *p < 0.05, **p < 0.01, ***p < 0.001, ****p < 0.0001 (calculated with ANOVA-Bonferroni-Holm). (**D**) FPKM values of validated genes within IC, RH and MY. FPKM- Fragments per kilobase of exon per million fragments mapped; TRI5 had the highest FPKM value within IC (109.3), while expression in RH (7.1) and MY (3.0) was low. RT-qPCR showed a significant 3-fold up-regulation in IC compared to RH, and a 10-fold up-regulation in IC compared to MY, and no significant differences in expression between RH and MY. The RT-qPCR results for FgPKS12 and FgPE1 were also in corroboration with the transcriptome data. FgPKS12 had the highest FPKM value in RH (11.4) compared to MY (FPKM = 0.68) and IC (FPKM = 0.25). FgPE1 was differently regulated, with the highest expression in MY (FPKM = 1307.3) and a down-regulation during palea colonization in RH (FPKM = 333.7) and also in IC (FPKM = 788.6). IC: infection cushions; RH: runner hyphae and MY: mycelium.
