# Supplementary material for: Infection cushions of Fusarium graminearum are fungal arsenals for wheat infection
Source: Mol Plant Pathol. 2020 Jun 23;21(8):1070–87. doi: 10.1111/mpp.12960 (PMC7368127; doi:10.1111/mpp.12960)
Supplement: Supplementary file 2 [file MPP-21-1070-s002.docx]

**
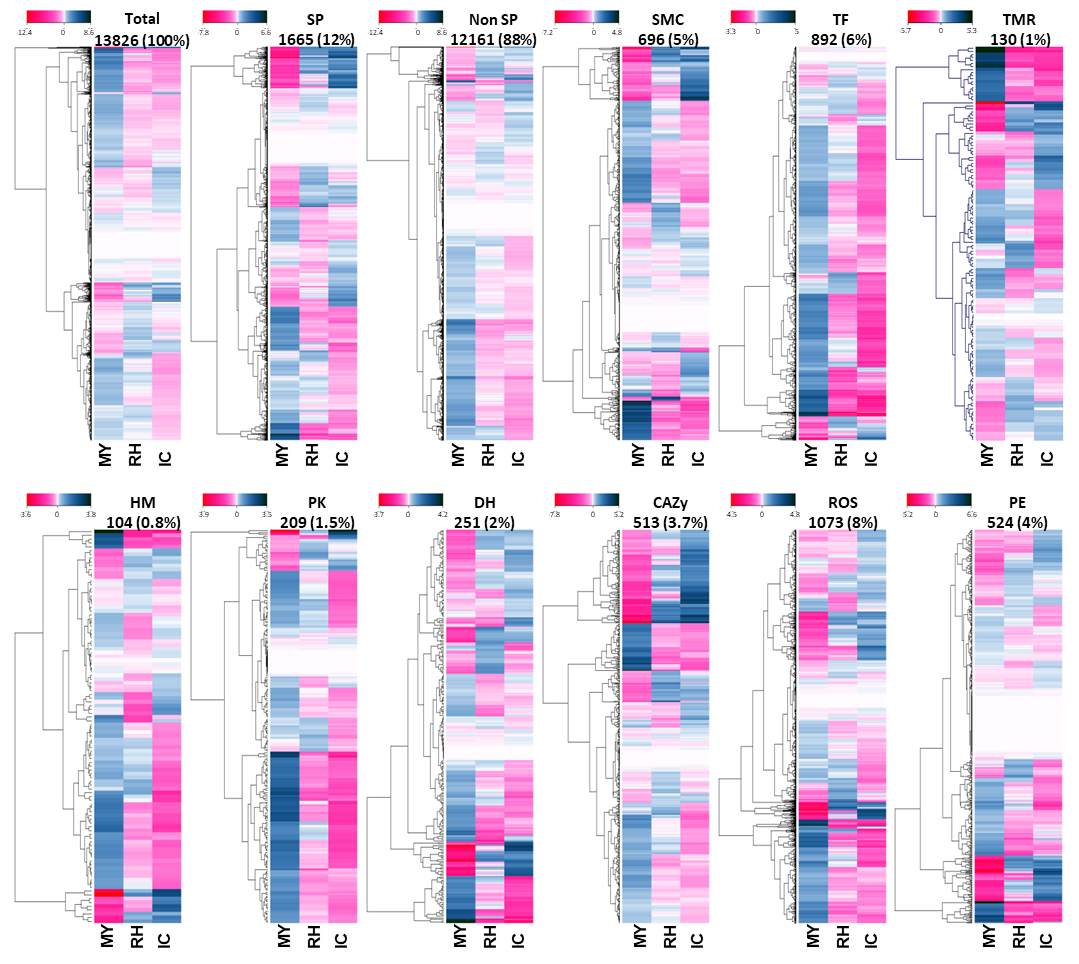
**

**Fig S2. Gene expression profiling during initial wheat palea infection.** Gene expression heat maps feature the regulation of genes in twelve categories. Under-represented (light magenta to dark magenta) and over-represented (light blue to dark blue) transcripts are shown as Log_2_ FC compared to the general mean expression level in all three cell types. Equally regulated genes are shown in white. Total: profiling of all 13826 genes; secreted proteins (SP; 1665 genes, 12% of all 13826 predicted genes), non-secreted proteins (non SP; 12161; 88%), secondary metabolite cluster genes (SMC; 696 genes, 5%), transcription factors (TF; 892 genes, 6%), transmembrane receptors (TMR; 130 genes, 1%), histone modifying proteins (HM; 104 genes, 1%), protein kinases/phosphatases (PK; 209 genes, 1.5%), dehydrogenases (DH; 251 genes, 2%), carbohydrate-active enzymes (CAZymes; 513 genes, 3.7%), genes for reactive oxygen species metabolism (ROS; 1073 genes, 8%), and putative effector proteins (PE, 524 genes, 4%) MY: mycelium grown in CM, RH: runner hyphae, IC: infection cushions. Non-SP, TF, HM and PK were under-represented in genes differentially expressed in RH and/or IC compared to MY, while, SP, CAZymes and ROS were over-represented. SMC, TMR, DH and PE show a diverse regulation pattern.
