# Supplementary material for: Infection cushions of Fusarium graminearum are fungal arsenals for wheat infection
Source: Mol Plant Pathol. 2020 Jun 23;21(8):1070–87. doi: 10.1111/mpp.12960 (PMC7368127; doi:10.1111/mpp.12960)
Supplement: Supplementary file 3 [file MPP-21-1070-s003.docx]

**FgPE1_Prom_**

**Hygromycin**

**mCherry**

**A**


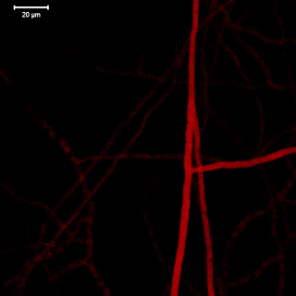

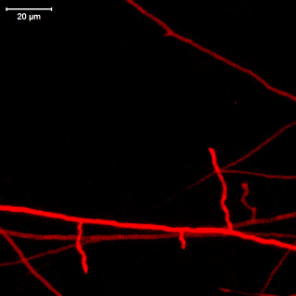

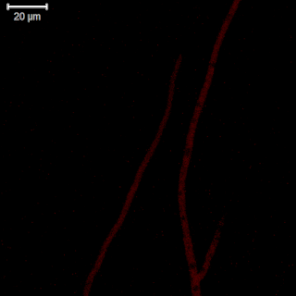

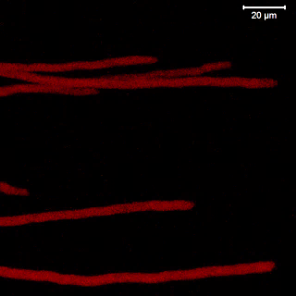

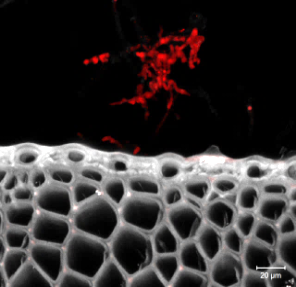


**IC**


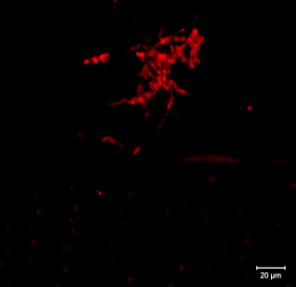


**IC**


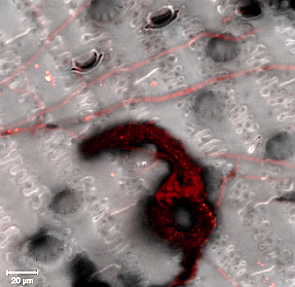

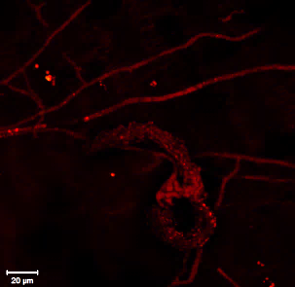


**RH**

**RH**

**IC**

**IC**

**PCW**

**PCW**

**B**

**C**

**D**

**E**

**F**

**G**

**H**

**I**

**Fig S3.** **Transcription of the FgPE1 gene is regulated by plant inducing factors.** Expression of the FgPE1 gene was determined by visualization of the reporter gene mCherry driven by the FgPE1 gene native promoter (**A**). Transformants containing the FgPE1_Prom_::mCherry construct were grown on wheat paleae (**B**-**E**), wheat media (**F**-**G**) or complete media (**H**-**I**). At 6 dpi of wheat palea infection, FgPE1 promoter is expressed in runner hyphae (RH) and infection cushions (IC) (**B** and **C**). Cross-section of an IC presenting high expression of FgPE1 promoter (**D** and **E**). While hyphae grown in wheat media show a strong mCherry expression at day 1 (**F**), hyphae grown in CM for 1 day show an almost undetectable mCherry signal (**H**). However, a strong mCherry signal is present at 3 days of growth independently of the media (**G** and **I**). Pictures are maximum intensity projections of 15 to 30 pictures. RH: runner hyphae, IC: infection cushion, PCW: plant cell wall, Scale bar = 20 µm.
