# Supplementary material for: Infection cushions of Fusarium graminearum are fungal arsenals for wheat infection
Source: Mol Plant Pathol. 2020 Jun 23;21(8):1070–87. doi: 10.1111/mpp.12960 (PMC7368127; doi:10.1111/mpp.12960)
Supplement: Supplementary file 4 [file MPP-21-1070-s004.docx]

**
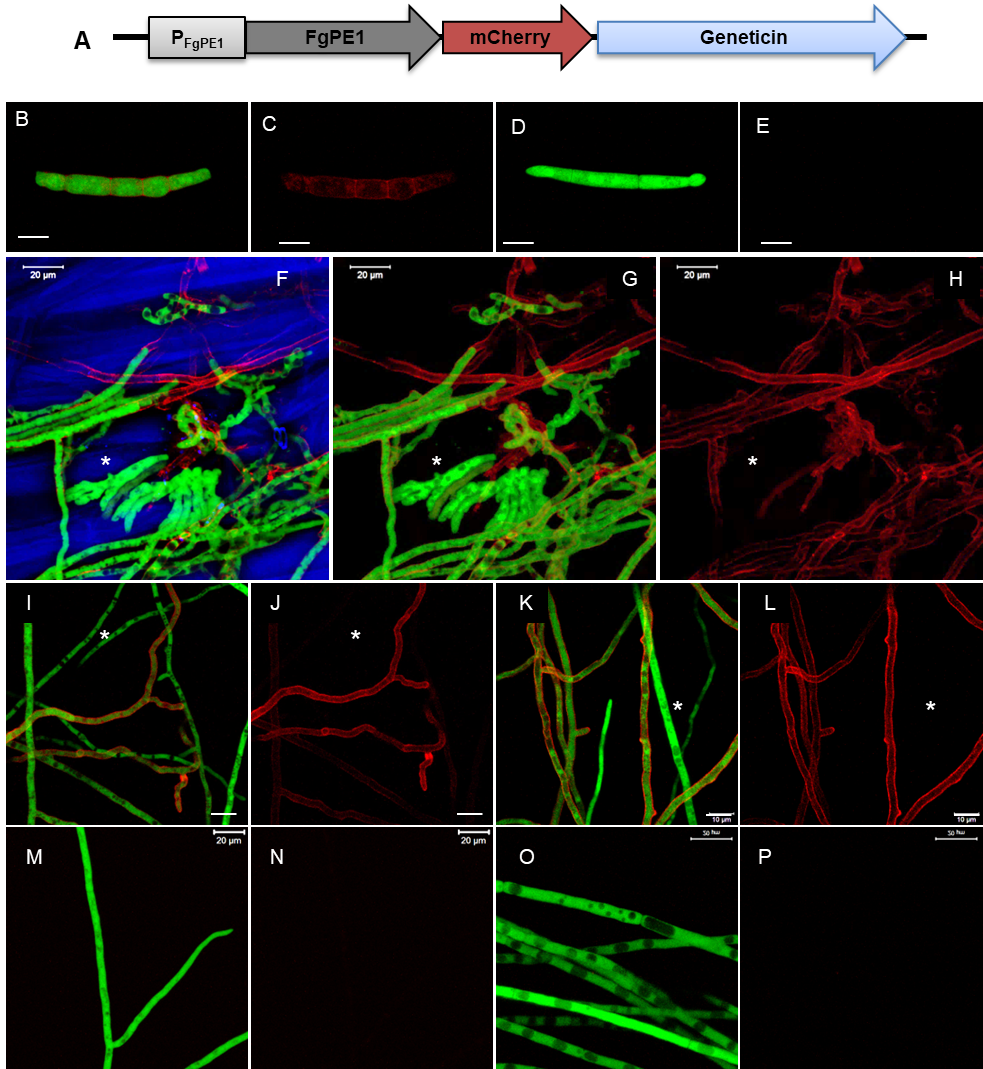
**

**Fig S4. The FgPE1 putative effector protein production is plant dependent.** (**A**) Schematic representation of the localization construct containing the FgPE1 native promoter and ORF without stop codon fused to mCherry and containing the geneticin selectable marker. Conidia of the transformants carrying the localization construct FgPE1_Prom_::FgPE1::mCherry and constitutive GFP presented abundant mCherry (**B, C**) in comparison to WT-GFP (**D**,**E**) used as negative control of autofluorescence. The FgPE1::mCherry transformants were grown on palea for 10 days (**F-H**), wheat media or complete media for 1 (**I**, **J** and **M**, **N**) or 3 days (**K**, **L** and **O**, **P**). When grown on wheat palea (**F**-**H**) or wheat media (**I**-**L**), the FgPE1 protein localizes around old hyphae, but not young strongly growing hyphae. However, no mCherry signal is observed when grown in CM (**M**-**P**). Pictures were taken with a confocal microscope (CLSM Zeiss) and they are maximum intensity projections of 15 to 30 pictures. Overlay image of photos taken with DAPI, mCherry or GFP filters individually and combined were prepared using the Zeiss AxioVision software. Stars indicate young hyphae without FgPE1 protein (**F**-**L**). Scale bar = 20µm (**F**-**H**, **M**-**P**) and 10µm (**B**-**E**, **I**-**L**).
