# Supplementary material for: Infection cushions of Fusarium graminearum are fungal arsenals for wheat infection
Source: Mol Plant Pathol. 2020 Jun 23;21(8):1070–87. doi: 10.1111/mpp.12960 (PMC7368127; doi:10.1111/mpp.12960)
Supplement: Supplementary file 5 [file MPP-21-1070-s005.docx]

**
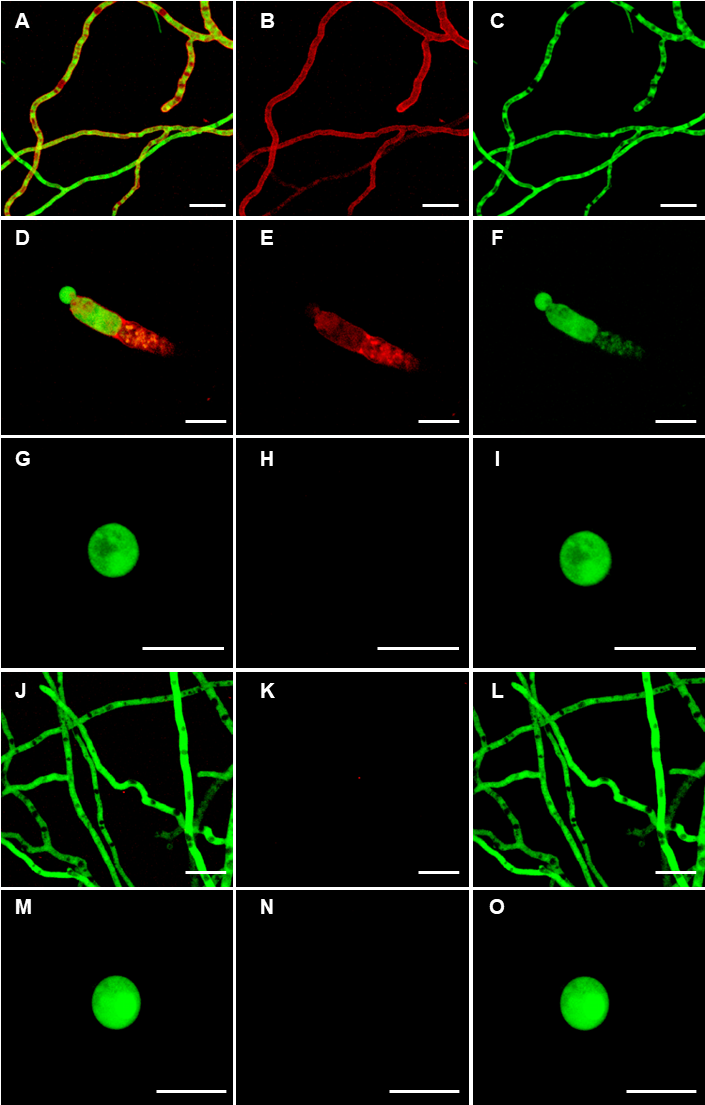
Fig S5. FgPE1 is localized at the fungal cell wall**. Production of FgPE1 protein was induced by growing the FgPE1::mCherry mutant carrying the localization construct FgPE1_Prom_::FgPE1::mCherry and constitutive GFP (**A**-**I**) and the WT-GFP like strain (**J**-**O**) in wheat liquid media for 20 h. Mycelia of both strains were digested with an enzymatic cell wall degrading cocktail for 1 to 3 hours. Micrographs of undigested mycelia (**A**-**C** and **J**-**L**), hyphae with partially digested cell walls (**D**-**F**) and released protoplasts (**G**-**I** and **M**-**O**) were taken with a confocal microscope (CLSM Zeiss). The released FgPE1::mCherry and WT-GFP protoplast lack a mCherry signal (**H**, **N**). In contrast, the untreated and partially digested hyphae of FgPE-mCherry (**B**), but not of WT-GFP (**N**) presented a mCherry signal. Micrographs are maximum intensity projections of 15 pictures. Overlay image of photos taken with mCherry or GFP filters individually and combined were prepared using the Zeiss AxioVision software. Scale bar = 20 µm (**A**-**C** and **J**-**L**) or 10 µm (**D**-**I** and **M**-**O**).
