# Supplementary material for: Infection cushions of Fusarium graminearum are fungal arsenals for wheat infection
Source: Mol Plant Pathol. 2020 Jun 23;21(8):1070–87. doi: 10.1111/mpp.12960 (PMC7368127; doi:10.1111/mpp.12960)
Supplement: Supplementary file 6 [file MPP-21-1070-s006.docx]

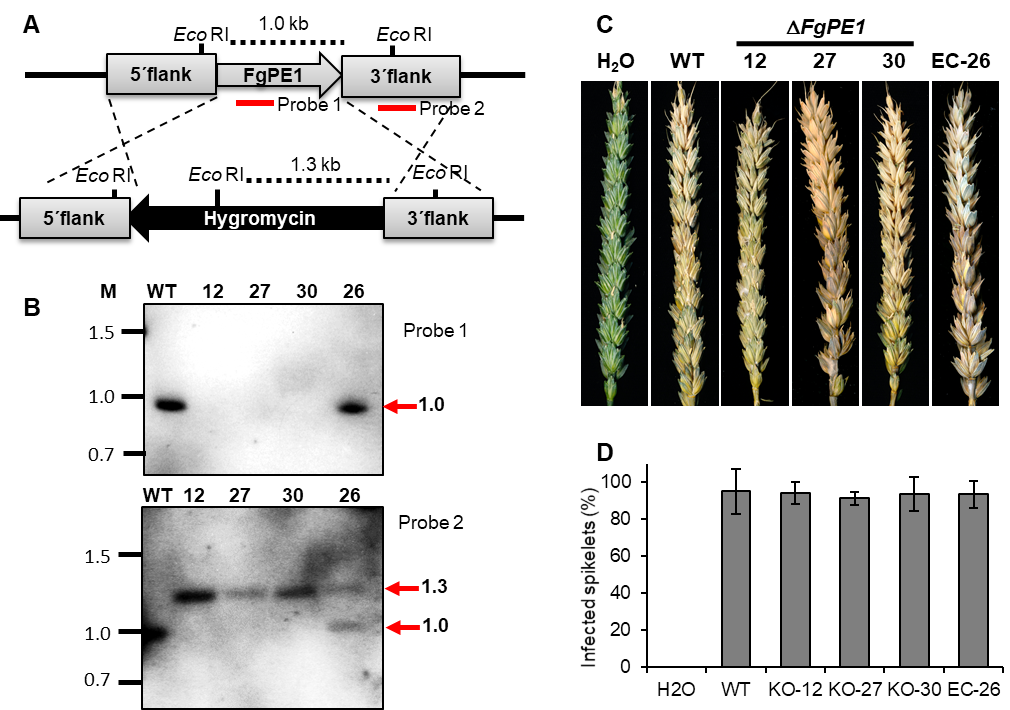


**Fig S6. FgPE1 gene is dispensable for virulence.** The FGSG_04213 gene named FgPE1 encoding for a small protein (166 aa) with a signal peptide (between 15-16 aa) and containing an Alt-A1 domain was selected for characterization. (**A**) The ORF of FgPE1 gene was replaced by hygromycin cassette. (**B**) Southern confirmed the integration of the deletion construct containing the hygromycin cassette by homologous recombination in the FgPE1 gene locus using probe 1 or probe 2. M: DNA ladder; WT: wild type; 12, 27, 30: deletion mutants; 26: ectopic. (**C**) Spikes of the wheat cultivar Nandu were inoculated with the deletion mutants Δ*FgPE1*- 12, 27 and 30 as well as the ectopic mutant 26 and the wild type strain. The deletion mutants showed similar infection levels to that of the wild type. Water was used as a negative control. Pictures are representative of 10 spikes per each treatment. Symptoms were assessed at 21 dpi. (**D**) Percentage of infected spikelets was determined by counting the total number of spikelets and the amount of infected spikelets. Fully infected spike was 100%. Error bars indicate standard deviations calculated from 5 spikes for each treatment and 2 independent experiments (n = 10).
