# Supplementary material for: Infection cushions of Fusarium graminearum are fungal arsenals for wheat infection
Source: Mol Plant Pathol. 2020 Jun 23;21(8):1070–87. doi: 10.1111/mpp.12960 (PMC7368127; doi:10.1111/mpp.12960)
Supplement: Supplementary file 7 [file MPP-21-1070-s007.docx]

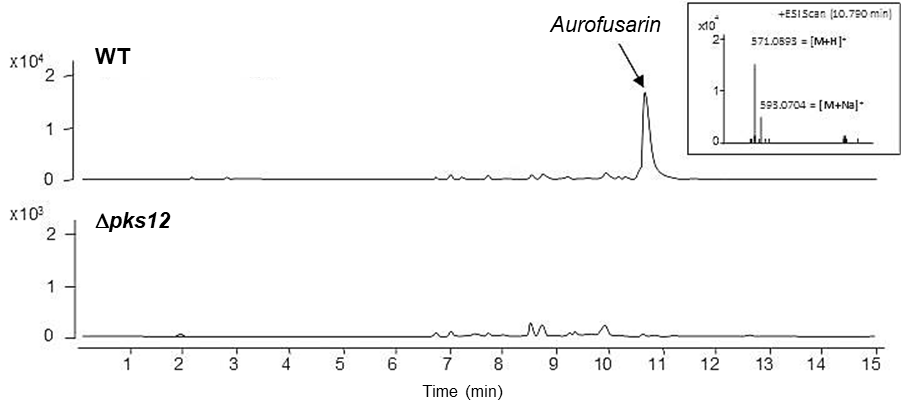


**Fig S7. *Δpks12* mutant does not produce aurofusarin.** LC-MS chromatogram of a phosphate buffer extract of *F. graminearum* wild type (top panel) and Δ*pks12* mutant (bottom panel). The aurofusarin peak (10.79 min) was identified by comparing with an authentic standard, not shown, and verified by UV/VIS-spectrum and adduct pattern (shown in the inserted box; intensity as a function of m/z).
