# Supplementary material for: Infection cushions of Fusarium graminearum are fungal arsenals for wheat infection
Source: Mol Plant Pathol. 2020 Jun 23;21(8):1070–87. doi: 10.1111/mpp.12960 (PMC7368127; doi:10.1111/mpp.12960)
Supplement: Supplementary file 8 [file MPP-21-1070-s008.docx]

**Table S1. Pearson correlation of the analyzed RNAseq data.** The correlation is indicated by a color gradient from green to red. Replicates per cell type used for transcriptional analyses are highlighted in orange, blue and yellow for IC, RH and MY, respectively.

| **Samples/**  **replicates** | | **IC** | | | **RH** | | | **MY** | | | |
| --- | --- | --- | --- | --- | --- | --- | --- | --- | --- | --- | --- |
|  |  | 1 | 2 | 3 | 1 | 2 | 3 | 1 | 2 | 3 |  |
| **IC** | 1 | 1 | 0.951 | 0.883 | 0.917 | 0.947 | 0.926 | 0.748 | 0.79 | 0.823 |  |
|  | 2 | 0.951 | 1 | 0.874 | 0.879 | 0.918 | 0.916 | 0.704 | 0.75 | 0.777 |  |
|  | 3 | 0.883 | 0.874 | 1 | 0.869 | 0.888 | 0.881 | 0.749 | 0.767 | 0.791 |  |
| **RH** | 1 | 0.917 | 0.879 | 0.869 | 1 | 0.938 | 0.896 | 0.789 | 0.836 | 0.864 |  |
|  | 2 | 0.947 | 0.918 | 0.888 | 0.938 | 1 | 0.928 | 0.772 | 0.815 | 0.844 |  |
|  | 3 | 0.926 | 0.916 | 0.881 | 0.896 | 0.928 | 1 | 0.762 | 0.794 | 0.82 |  |
| **MY** | 1 | 0.748 | 0.704 | 0.749 | 0.789 | 0.772 | 0.762 | 1 | 0.855 | 0.887 |  |
|  | 2 | 0.79 | 0.75 | 0.767 | 0.836 | 0.815 | 0.794 | 0.855 | 1 | 0.938 |  |
|  | 3 | 0.823 | 0.777 | 0.791 | 0.864 | 0.844 | 0.82 | 0.887 | 0.938 | 1 |  |
