# Supplementary material for: Infection cushions of Fusarium graminearum are fungal arsenals for wheat infection
Source: Mol Plant Pathol. 2020 Jun 23;21(8):1070–87. doi: 10.1111/mpp.12960 (PMC7368127; doi:10.1111/mpp.12960)
Supplement: Supplementary file 9 [file MPP-21-1070-s009.docx]

**Table S2. 50 more up-regulated genes in RH compared to MY.**

|  | | **Description** | | **Log2**  **Fold change**  **RH vs MY** | **P value** | **q value** |
| --- | --- | --- | --- | --- | --- | --- |
| **Nr** | **Locus** | **MIPS** | **IPRO** |  |  |  |
| 1 | FGSG_17054 | hypothetical protein | n.d. | 18.219 | 5.00E-05 | 0.00042973 |
| 2 | FGSG_08737 | probable woronin body major protein precursor | IPR001884 Translation elongation factor IF5A; IPR008991 Translation protein SH3-like; IPR012340 Nucleic acid-binding, OB-fold; IPR014722 Translation protein SH3-like, subgroup; IPR016027 Nucleic acid-binding, OB-fold-like | 12.452 | 1 | 1 |
| 3 | FGSG_13057 | hypothetical protein | n.d. | 12.189 | 5.00E-05 | 0.00042973 |
| 4 | FGSG_10999 | endo-1,4-beta-xylanase | IPR001137 Glycoside hydrolase, family 11; IPR008985 Concanavalin A-like lectin/glucanase; IPR013319 Glycoside hydrolase, family 11/12, catalytic domain; IPR018208 Glycoside hydrolase, family 11, active site | 11.088 | 0.0011 | 0.00660999 |
| 5 | FGSG_07558 | conserved hypothetical protein | IPR009104 Sea anemone cytolysin; IPR009960 Fungal fruit body lectin; IPR015926 Cytolysin/lectin | 10.906 | 1 | 1 |
| 6 | FGSG_06692 | probable DDR48 - heat shock protein | n.d. | 10.462 | 1 | 1 |
| 7 | FGSG_06445 | probable endo-1,4-beta-xylanase | IPR001000 Glycoside hydrolase, family 10; IPR013781 Glycoside hydrolase, subgroup, catalytic domain; IPR017853 Glycoside hydrolase, superfamily | 10.381 | 0.25655 | 0.420041 |
| 8 | FGSG_16834 | hypothetical protein | n.d. | 10.341 | 0.00625 | 0.027529 |
| 9 | FGSG_12456 | hypothetical protein | n.d. | 10.314 | 5.00E-05 | 0.00042973 |
| 10 | FGSG_04745 | related to antifungal protein | IPR022706 Antifungal protein; IPR023112 Antifungal protein domain | 10.179 | 0.2317 | 0.39427 |
| 11 | FGSG_00793 | conserved hypothetical protein | n.d. | 9.387 | 0.00275 | 0.0142272 |
| 12 | FGSG_03624 | probable endo-1,4-beta-xylanase A precursor | IPR001137 Glycoside hydrolase, family 11; IPR008985 Concanavalin A-like lectin/glucanase; IPR013319 Glycoside hydrolase, family 11/12, catalytic domain; IPR018208 Glycoside hydrolase, family 11, active site | 8.948 | 0.0014 | 0.00810642 |
| 13 | FGSG_03588 | related to integral membrane protein PTH11 | n.d. | 8.894 | 0.21605 | 0.376863 |
| 14 | FGSG_00237 | related to trichothecene 3-O-acetyltransferase | IPR003480 Transferase; IPR023213 Chloramphenicol acetyltransferase-like domain | 8.573 | 0.25735 | 0.420853 |
| 15 | FGSG_15273 | hypothetical protein | n.d. | 8.447 | 0.01345 | 0.049819 |
| 16 | FGSG_11304 | related to endo-1,4-beta-xylanase | IPR000254 Cellulose-binding domain, fungal; IPR001000 Glycoside hydrolase, family 10; IPR013781 Glycoside hydrolase, subgroup, catalytic domain; IPR017853 Glycoside hydrolase, superfamily | 8.059 | 5.00E-05 | 0.00042973 |
| 17 | FGSG_03628 | probable cellulose 1,4-beta-cellobiosidase II precursor | IPR000254 Cellulose-binding domain, fungal; IPR001524 Glycoside hydrolase, family 6, conserved site; IPR016288 1, 4-beta cellobiohydrolase | 8.004 | 5.00E-05 | 0.00042973 |
| 18 | FGSG_07988 | conserved hypothetical protein | n.d. | 7.863 | 0.00185 | 0.010243 |
| 19 | FGSG_03695 | related to endoglucanase IV precursor | IPR005103 Glycoside hydrolase, family 61 | 7.637 | 0.2429 | 0.406304 |
| 20 | FGSG_11487 | related to endo-1,4-beta-xylanase | IPR001000 Glycoside hydrolase, family 10; IPR013781 Glycoside hydrolase, subgroup, catalytic domain; IPR017853 Glycoside hydrolase, superfamily | 7.437 | 0.0275 | 0.0858202 |
| 21 | FGSG_11303 | related to isotrichodermin C-15 hydroxylase (cytochrome P-450 monooxygenase CYP65A1) | IPR001128 Cytochrome P450; IPR002401 Cytochrome P450, E-class, group I | 7.213 | 5.00E-05 | 0.00042973 |
| 22 | FGSG_11495 | related to hexose transporter protein | IPR005828 General substrate transporter; IPR016196 Major facilitator superfamily domain, general substrate transporter; IPR020846 Major facilitator superfamily domain | 7.105 | 5.00E-05 | 0.00042973 |
| 23 | FGSG_11036 | related to esterase D | IPR001375 Peptidase S9, prolyl oligopeptidase, catalytic domain | 7.033 | 0.2566 | 0.420085 |
| 24 | FGSG_03591 | probable endochitinase | IPR001223 Glycoside hydrolase, family 18, catalytic domain; IPR001579 Glycoside hydrolase, chitinase active site; IPR011583 Chitinase II; IPR013781 Glycoside hydrolase, subgroup, catalytic domain; IPR017853 Glycoside hydrolase, superfamily | 6.947 | 0.2264 | 0.388425 |
| 25 | FGSG_04848 | probable rhamnogalacturonan acetylesterase precursor | IPR001087 Lipase, GDSL; IPR013830 Esterase, SGNH hydrolase-type; IPR013831 Esterase, SGNH hydrolase-type, subgroup | 6.934 | 5.00E-05 | 0.00042973 |
| 26 | FGSG_04741 | conserved hypothetical protein | n.d. | 6.815 | 5.00E-05 | 0.00042973 |
| 27 | FGSG_11302 | conserved hypothetical protein | n.d. | 6.795 | 5.00E-05 | 0.00042973 |
| 28 | FGSG_06452 | related to deacetylase | IPR002509 Polysaccharide deacetylase; IPR011330 Glycoside hydrolase/deacetylase, beta/alpha-barrel | 6.670 | 5.00E-05 | 0.00042973 |
| 29 | FGSG_08697 | conserved hypothetical protein | IPR007274 Ctr copper transporter | 6.599 | 0.0013 | 0.00761312 |
| 30 | FGSG_08011 | related to cellulose binding protein CEL1 | IPR000254 Cellulose-binding domain, fungal; IPR005103 Glycoside hydrolase, family 61 | 6.596 | 5.00E-05 | 0.00042973 |
| 31 | FGSG_03585 | conserved hypothetical protein | n.d. | 6.385 | 5.00E-05 | 0.00042973 |
| 32 | FGSG_03190 | conserved hypothetical protein | IPR008579 Domain of unknown function DUF861, cupin-3; IPR011051 Cupin, RmlC-type; IPR014710 RmlC-like jelly roll fold | 6.350 | 0.1314 | 0.26839 |
| 33 | FGSG_06463 | related to alpha-L-arabinofuranosidase A precursor | IPR010720 Alpha-L-arabinofuranosidase, C-terminal; IPR017853 Glycoside hydrolase, superfamily | 6.346 | 0.00085 | 0.00530621 |
| 34 | FGSG_03632 | related to cellulose binding protein CEL1 | IPR005103 Glycoside hydrolase, family 61 | 6.342 | 0.00235 | 0.0124964 |
| 35 | FGSG_10655 | related to ferric reductase FRE2 precursor | IPR013112 FAD-binding 8; IPR013121 Ferric reductase, NAD binding; IPR013130 Flavoprotein transmembrane component | 6.277 | 5.00E-05 | 0.00042973 |
| 36 | FGSG_04518 | conserved hypothetical protein | IPR009053 Prefoldin | 6.270 | 5.00E-05 | 0.00042973 |
| 37 | FGSG_06062 | conserved hypothetical protein | IPR001660 Sterile alpha motif domain; IPR013761 Sterile alpha motif/pointed domain; IPR021129 Sterile alpha motif, type 1 | 6.195 | 5.00E-05 | 0.00042973 |
| 38 | FGSG_01770 | conserved hypothetical protein | n.d. | 6.193 | 5.00E-05 | 0.00042973 |
| 39 | FGSG_13515 | hypothetical protein | n.d. | 6.163 | 0.15755 | 0.304579 |
| 40 | FGSG_12519 | probable aspartate aminotransferase, cytoplasmic | IPR002872 Proline dehydrogenase; IPR015659 Proline oxidase | 6.148 | 5.00E-05 | 0.00042973 |
| 41 | FGSG_03898 | related to isoamyl alcohol oxidase | IPR006093 Oxygen oxidoreductase covalent FAD-binding site; IPR006094 FAD linked oxidase, N-terminal; IPR012951 Berberine/berberine-like; IPR016166 FAD-binding, type 2; IPR016167 FAD-binding, type 2, subdomain 1; IPR016168 FAD-linked oxidase, FAD-binding, subdomain 2 | 6.133 | 0.0087 | 0.035646 |
| 42 | FGSG_07639 | related to xylosidase/arabinosidase | IPR006710 Glycoside hydrolase, family 43; IPR008985 Concanavalin A-like lectin/glucanase; IPR013320 Concanavalin A-like lectin/glucanase, subgroup; IPR023296 Glycosyl hydrolase family 43, five-bladed beta-propellor domain | 6.120 | 0.1807 | 0.334508 |
| 43 | FGSG_03457 | probable cutinase 1 precursor | IPR000675 Cutinase; IPR011150 Cutinase, monofunctional | 6.104 | 0.2429 | 0.406304 |
| 44 | FGSG_04746 | conserved hypothetical protein | IPR022085 Protein of unknown function DUF3632 | 6.078 | 0.2429 | 0.406304 |
| 45 | FGSG_10784 | conserved hypothetical protein | n.d. | 6.067 | 0.00385 | 0.0187311 |
| 46 | FGSG_01771 | conserved hypothetical protein | n.d. | 6.018 | 5.00E-05 | 0.00042973 |
| 47 | FGSG_09066 | conserved hypothetical protein | n.d. | 5.998 | 5.00E-05 | 0.000429738 |
| 48 | FGSG_01829 | related to aldose 1-epimerase | IPR008183 Aldose 1-epimerase; IPR011013 Glycoside hydrolase-type carbohydrate-binding; IPR014718 Glycoside hydrolase-type carbohydrate-binding, subgroup; IPR015443 Aldose 1-epimerase, subgroup | 5.993 | 0.00025 | 0.00183247 |
| 49 | FGSG_02672 | probable cytochrome P450 monooxygenase (lovA) | IPR001128 Cytochrome P450; IPR002403 Cytochrome P450, E-class, group IV; IPR017972 Cytochrome P450, conserved site | 5.980 | 0.2566 | 0.420085 |
| 50 | FGSG_11428 | probable feruloyl esterase B precursor (subclass of the carboxylic acid esterases) | IPR010126 Esterase, PHB depolymerase | 5.963 | 0.02705 | 0.0847727 |
