# Supplementary material for: Infection cushions of Fusarium graminearum are fungal arsenals for wheat infection
Source: Mol Plant Pathol. 2020 Jun 23;21(8):1070–87. doi: 10.1111/mpp.12960 (PMC7368127; doi:10.1111/mpp.12960)
Supplement: Supplementary file 10 [file MPP-21-1070-s010.docx]

**Table S3. 50 more up-regulated genes in IC compared to MY.**

|  | | **Description** | | **Log2**  **Fold change**  **IC vs MY** | **P value** | **q value** |
| --- | --- | --- | --- | --- | --- | --- |
| **Nr** | **Locus** | **MIPS** | **IPRO** |  |  |  |
| 1 | FGSG_17054 | hypothetical protein | n.d. | 19.116 | 5.00E-05 | 0.00042973 |
| 2 | FGSG_07822 | conserved hypothetical protein | n.d. | 12.962 | 1 | 1 |
| 3 | FGSG_10999 | endo-1,4-beta-xylanase | IPR001137 Glycoside hydrolase, family 11; IPR008985 Concanavalin A-like lectin/glucanase; IPR013319 Glycoside hydrolase, family 11/12, catalytic domain; IPR018208 Glycoside hydrolase, family 11, active site | 12.345 | 0.0011 | 0.00660999 |
| 4 | FGSG_13057 | hypothetical protein | n.d. | 12.305 | 5.00E-05 | 0.00042973 |
| 5 | FGSG_12456 | hypothetical protein | n.d. | 11.804 | 5.00E-05 | 0.00042973 |
| 6 | FGSG_08737 | probable woronin body major protein precursor | IPR001884 Translation elongation factor IF5A; IPR008991 Translation protein SH3-like; IPR012340 Nucleic acid-binding, OB-fold; IPR014722 Translation protein SH3-like, subgroup; IPR016027 Nucleic acid-binding, OB-fold-like | 11.414 | 1 | 1 |
| 7 | FGSG_06445 | probable endo-1,4-beta-xylanase | IPR001000 Glycoside hydrolase, family 10; IPR013781 Glycoside hydrolase, subgroup, catalytic domain; IPR017853 Glycoside hydrolase, superfamily | 11.291 | 0.25655 | 0.420041 |
| 8 | FGSG_03695 | related to endoglucanase IV precursor | IPR005103 Glycoside hydrolase, family 61 | 11.066 | 0.2429 | 0.406304 |
| 9 | FGSG_04745 | related to antifungal protein | IPR022706 Antifungal protein; IPR023112 Antifungal protein domain | 10.729 | 0.2317 | 0.39427 |
| 10 | FGSG_03624 | probable endo-1,4-beta-xylanase A precursor | IPR001137 Glycoside hydrolase, family 11; IPR008985 Concanavalin A-like lectin/glucanase; IPR013319 Glycoside hydrolase, family 11/12, catalytic domain; IPR018208 Glycoside hydrolase, family 11, active site | 10.089 | 0.0014 | 0.00810642 |
| 11 | FGSG_06692 | probable DDR48 - heat shock protein | n.d. | 10.065 | 1 | 1 |
| 12 | FGSG_11487 | related to endo-1,4-beta-xylanase | IPR001000 Glycoside hydrolase, family 10; IPR013781 Glycoside hydrolase, subgroup, catalytic domain; IPR017853 Glycoside hydrolase, superfamily | 9.741 | 0.0275 | 0.0858202 |
| 13 | FGSG_00793 | conserved hypothetical protein | n.d. | 9.694 | 0.00275 | 0.0142272 |
| 14 | FGSG_16834 | hypothetical protein | n.d. | 9.596 | 0.0248 | 0.0793425 |
| 15 | FGSG_11047 | conserved hypothetical protein | n.d. | 9.542 | 0.21605 | 0.376863 |
| 16 | FGSG_10784 | conserved hypothetical protein | n.d. | 9.526 | 0.00385 | 0.0187311 |
| 17 | FGSG_00237 | related to trichothecene 3-O-acetyltransferase | IPR003480 Transferase; IPR023213 Chloramphenicol acetyltransferase-like domain | 9.039 | 0.25735 | 0.420853 |
| 18 | FGSG_11488 | related to cellulose binding protein CEL1 | IPR005103 Glycoside hydrolase, family 61 | 8.906 | 5.00E-05 | 0.00042973 |
| 19 | FGSG_15917 | probable endo-1,4-beta-xylanase A precursor | IPR001137 Glycoside hydrolase, family 11; IPR008985 Concanavalin A-like lectin/glucanase; IPR013319 Glycoside hydrolase, family 11/12, catalytic domain | 8.886 | 0.2486 | 0.412203 |
| 20 | FGSG_03190 | conserved hypothetical protein | IPR008579 Domain of unknown function DUF861, cupin-3; IPR011051 Cupin, RmlC-type; IPR014710 RmlC-like jelly roll fold | 8.836 | 0.1314 | 0.26839 |
| 21 | FGSG_11036 | related to esterase D | IPR001375 Peptidase S9, prolyl oligopeptidase, catalytic domain | 8.657 | 0.2566 | 0.420085 |
| 22 | FGSG_03457 | probable cutinase 1 precursor | IPR000675 Cutinase; IPR011150 Cutinase, monofunctional | 8.545 | 0.2429 | 0.406304 |
| 23 | FGSG_15273 | hypothetical protein | n.d. | 8.419 | 0.01345 | 0.049819 |
| 24 | FGSG_03632 | related to cellulose binding protein CEL1 | IPR005103 Glycoside hydrolase, family 61 | 8.417 | 0.00235 | 0.0124964 |
| 25 | FGSG_03628 | probable cellulose 1,4-beta-cellobiosidase II precursor | IPR000254 Cellulose-binding domain, fungal; IPR001524 Glycoside hydrolase, family 6, conserved site; IPR016288 1, 4-beta cellobiohydrolase | 8.273 | 5.00E-05 | 0.00042973 |
| 26 | FGSG_11304 | related to endo-1,4-beta-xylanase | IPR000254 Cellulose-binding domain, fungal; IPR001000 Glycoside hydrolase, family 10; IPR013781 Glycoside hydrolase, subgroup, catalytic domain; IPR017853 Glycoside hydrolase, superfamily | 8.271 | 5.00E-05 | 0.00042973 |
| 27 | FGSG_04848 | probable rhamnogalacturonan acetylesterase precursor | IPR001087 Lipase, GDSL; IPR013830 Esterase, SGNH hydrolase-type; IPR013831 Esterase, SGNH hydrolase-type, subgroup | 8.243 | 5.00E-05 | 0.00042973 |
| 28 | FGSG_03166 | conserved hypothetical protein | n.d. | 8.194 | 0.2429 | 0.406304 |
| 29 | FGSG_07558 | conserved hypothetical protein | IPR009104 Sea anemone cytolysin; IPR009960 Fungal fruit body lectin; IPR015926 Cytolysin/lectin | 8.159 | 1 | 1 |
| 30 | FGSG_12514 | hypothetical protein | n.d. | 8.132 | 5.00E-05 | 0.00042973 |
| 31 | FGSG_02613 | conserved hypothetical protein | n.d. | 8.095 | 0.18375 | 0.338266 |
| 32 | FGSG_08021 | conserved hypothetical protein | IPR021476 Protein of unknown function DUF3129 | 8.070 | 0.00835 | 0.034528 |
| 33 | FGSG_03588 | related to integral membrane protein PTH11 | n.d. | 8.057 | 0.21605 | 0.376863 |
| 34 | FGSG_07988 | conserved hypothetical protein | n.d. | 8.038 | 0.00185 | 0.010243 |
| 35 | FGSG_10563 | conserved hypothetical protein | IPR002482 Peptidoglycan-binding Lysin subgroup; IPR018392 Peptidoglycan-binding lysin domain | 7.991 | 0.21605 | 0.376863 |
| 36 | FGSG_06452 | related to deacetylase | IPR002509 Polysaccharide deacetylase; IPR011330 Glycoside hydrolase/deacetylase, beta/alpha-barrel | 7.948 | 5.00E-05 | 0.00042973 |
| 37 | FGSG_08011 | related to cellulose binding protein CEL1 | IPR000254 Cellulose-binding domain, fungal; IPR005103 Glycoside hydrolase, family 61 | 7.935 | 5.00E-05 | 0.00042973 |
| 38 | FGSG_03454 | conserved hypothetical protein | n.d. | 7.826 | 5.00E-05 | 0.00042973 |
| 39 | FGSG_04647 | probable gEgh 16 protein | IPR021476 Protein of unknown function DUF3129 | 7.789 | 0.2499 | 0.413493 |
| 40 | FGSG_07625 | probable alpha-L-arabinofuranosidase precursor | IPR005193 Glycoside hydrolase, family 62, arabinosidase; IPR023296 Glycosyl hydrolase family 43, five-bladed beta-propellor domain | 7.562 | 0.00345 | 0.0171403 |
| 41 | FGSG_11315 | conserved hypothetical protein | n.d. | 7.541 | 5.00E-05 | 0.00042973 |
| 42 | FGSG_03535 | trichodiene oxygenase [cytochrome P450] | IPR001128 Cytochrome P450; IPR002401 Cytochrome P450, E-class, group I; IPR017972 Cytochrome P450, conserved site | 7.540 | 5.00E-05 | 0.00042973 |
| 43 | FGSG_02672 | probable cytochrome P450 monooxygenase (lovA) | IPR001128 Cytochrome P450; IPR002403 Cytochrome P450, E-class, group IV; IPR017972 Cytochrome P450, conserved site | 7.525 | 0.2566 | 0.420085 |
| 44 | FGSG_03144 | related to hexose transporter protein | IPR005828 General substrate transporter; IPR016196 Major facilitator superfamily domain, general substrate transporter; IPR020846 Major facilitator superfamily domain | 7.486 | 0.2264 | 0.388425 |
| 45 | FGSG_03150 | conserved hypothetical protein | IPR002575 Aminoglycoside phosphotransferase; IPR011009 Protein kinase-like domain; IPR015897 CHK kinase-like | 7.427 | 0.2264 | 0.388425 |
| 46 | FGSG_13515 | hypothetical protein | n.d. | 7.403 | 0.15755 | 0.304579 |
| 47 | FGSG_13761 | conserved hypothetical protein | n.d. | 7.399 | 0.1807 | 0.334508 |
| 48 | FGSG_11219 | related to putative tartrate transporter | IPR011701 Major facilitator superfamily; IPR016196 Major facilitator superfamily domain, general substrate transporter; IPR020846 Major facilitator superfamily domain | 7.366 | 0.0004 | 0.00276282 |
| 49 | FGSG_03494 | conserved hypothetical protein | IPR008949 Terpenoid synthase | 7.314 | 0.25415 | 0.417704 |
| 50 | FGSG_02386 | probable pectate lyase | IPR004898 Pectate lyase, catalytic; IPR011050 Pectin lyase fold/virulence factor; IPR012334 Pectin lyase fold | 7.161 | 5.00E-05 | 0.00042973 |
