# Supplementary material for: Infection cushions of Fusarium graminearum are fungal arsenals for wheat infection
Source: Mol Plant Pathol. 2020 Jun 23;21(8):1070–87. doi: 10.1111/mpp.12960 (PMC7368127; doi:10.1111/mpp.12960)
Supplement: Supplementary file 11 [file MPP-21-1070-s011.docx]

**Table S4.** **Infection regulated genes in gene families.** The color-coded heat-map considers the total number of infection up-regulated genes regarding their regulation in RH and IC compared to MY, from green (highest), to red (lowest). Non SP: non-secreted proteins, SP: putative secreted proteins, TF: transcription factors, TP: transporter proteins, HM: histone modifying proteins, PK: protein kinases/phosphatases, DH: dehydrogenases, CAZyme: carbohydrate-active enzymes, PE: putative effector proteins, ROS: proteins related to reactive oxygen species, TMR: transmembrane receptors, and no: genes without any annotation. Due to several genes belonging to more than one gene family, there are higher total number of genes in this table than in the annotated genome (see also Data set S1; Fig. 3).

| Regulation | non SP | | | | | | | | | | SP | | | | | | | | | | non SP | SP | total | |
| --- | --- | --- | --- | --- | --- | --- | --- | --- | --- | --- | --- | --- | --- | --- | --- | --- | --- | --- | --- | --- | --- | --- | --- | --- |
|  | TF | TP | HM | PK | DH | CAZyme | PE | ROS | TMR | no | TF | TP | HM | PK | DH | CAZyme | PE | ROS | TMR | no | total | total |  |  |
| plant-repressed | 260 | 159 | 19 | 68 | 37 | 63 | 0 | 151 | 28 | 1899 | 1 | 0 | 0 | 2 | 1 | 37 | 77 | 22 | 0 | 166 | 2684 | 306 | 2990 |  |
| plant-induced | 15 | 76 | 8 | 5 | 29 | 26 | 0 | 108 | 19 | 408 | 0 | 0 | 0 | 0 | 2 | 114 | 88 | 41 | 0 | 83 | 694 | 328 | 1022 |  |
| total | 275 | 235 | 27 | 73 | 66 | 89 | 0 | 259 | 47 | 2307 | 1 | 0 | 0 | 2 | 3 | 151 | 165 | 63 | 0 | 249 | 3378 | 634 | 4012 |  |
