# Supplementary material for: Infection cushions of Fusarium graminearum are fungal arsenals for wheat infection
Source: Mol Plant Pathol. 2020 Jun 23;21(8):1070–87. doi: 10.1111/mpp.12960 (PMC7368127; doi:10.1111/mpp.12960)
Supplement: Supplementary file 12 [file MPP-21-1070-s012.docx]

**Table S5.** **Infection up-regulated genes equally and differentially expressed in RH or IC in the gene families.** The color-coded heat-map considers the total of infection up-regulated genes regarding their regulation in infection cushions (IC) and runner hyphae (RH) from green (highest) and red (lowest). Non SP: non-secreted proteins, SP: putative secreted proteins, TF: transcription factors, TP: transporter proteins, HM: histone modifying proteins, PK: protein kinases/phosphatases, DH: dehydrogenases, CAZyme: carbohydrate-active enzymes, PE: putative effector proteins, ROS: proteins related to reactive oxygen species and TMR: transmembrane receptors. Due to several genes belonging to more than one gene family, there are higher total number of genes in this table than in the annotated genome (Data set S1).

| Regulation | non SP | | | | | | | | | SP | | | | | | | | | non SP | SP | total |
| --- | --- | --- | --- | --- | --- | --- | --- | --- | --- | --- | --- | --- | --- | --- | --- | --- | --- | --- | --- | --- | --- |
|  | TF | TP | HM | PK | DH | CAZyme | PE | ROS | TMR | TF | TP | HM | PK | DH | CAZyme | PE | ROS | TMR | total | total |  |
| non-regulated | 10 | 60 | 5 | 2 | 19 | 21 | 0 | 79 | 15 | 0 | 0 | 0 | 0 | 1 | 74 | 52 | 26 | 0 | 508 | 210 | 718 |
| RH up | 1 | 2 | 0 | 0 | 0 | 2 | 0 | 6 | 0 | 0 | 0 | 0 | 0 | 0 | 1 | 3 | 4 | 0 | 34 | 13 | 47 |
| IC up | 4 | 14 | 3 | 3 | 10 | 3 | 0 | 23 | 4 | 0 | 0 | 0 | 0 | 1 | 39 | 33 | 11 | 0 | 152 | 105 | 257 |
