# Supplementary material for: Infection cushions of Fusarium graminearum are fungal arsenals for wheat infection
Source: Mol Plant Pathol. 2020 Jun 23;21(8):1070–87. doi: 10.1111/mpp.12960 (PMC7368127; doi:10.1111/mpp.12960)
Supplement: Supplementary file 13 [file MPP-21-1070-s013.docx]

**Table S6. Infection down-regulated degradative CAZymes**

| **ID Gene** | **Reg ^a^** | **Family** | **Group ^b^** | **CAZy predicted function** |
| --- | --- | --- | --- | --- |
| FGSG_00721 | non | GH47 | FCM | a-1,2-mannosidase |
| FGSG_06305 | non | GH47 | FCM | a-1,2-mannosidase |
| FGSG_09931 | non | GH47 | FCM | a-1,2-mannosidase |
| FGSG_04584 | non | GH76 | FCM | a-1,6-mannotransglycosylase / a-1,6-mannanase |
| FGSG_16723 | non | GH38 | FCM | a-mannosidase |
| FGSG_04014 | non | GH16 | FCM | b-1,3(4)-endoglucanase |
| FGSG_01351 | non | GH132 | FCM | b-1,3-glucanase |
| FGSG_02807 | non | GH17 | FCM | b-1,3-glucanase |
| FGSG_03529 | non | GH17 | FCM | b-1,3-glucanase |
| FGSG_08534 | non | GH16 | FCM | b-1,3-glucanase |
| FGSG_08757 | non | GH81 | FCM | b-1,3-glucanase |
| FGSG_17666 | non | GH64 | FCM | b-1,3-glucanase |
| FGSG_09980 | non | GH72 | FCM | b-1,3-glucanosyltransferase |
| FGSG_01283 | non | GH20 | FCM | b-N-acetylglucosaminidase |
| FGSG_08395 | non | GH3 | FCM | b-N-acetylglucosaminidase |
| FGSG_07016 | non | GH76 | FCM | cell wall a-1,6-mannotransglycosylase / a-1,6-mannanase |
| FGSG_10387 | non | GH76 | FCM | cell wall a-1,6-mannotransglycosylase / a-1,6-mannanase |
| FGSG_13345 | non | GH76 | FCM | cell wall a-1,6-mannotransglycosylase / a-1,6-mannanase |
| FGSG_02720 | non | CBM18-GH16 | FCM | chitin b-1,3/1,6-glucanosyltransferase |
| FGSG_03017 | non | GH16 | FCM | chitin b-1,3/1,6-glucanosyltransferase |
| FGSG_05847 | non | CBM18-CE4-CBM18-CBM18 | FCM | chitin deacetylase |
| FGSG_10939 | non | GH18 | FCM | chitinase |
| FGSG_15828 | non | GH18 | FCM | chitinase |
| FGSG_03554 | non | CBM50 | FCM | chitin-binding protein |
| FGSG_06119 | non | GH16 | FCM | endo-1,3(4)-b-glucanase |
| FGSG_06110 | up | AA9 | PCWDC | lytic polysaccharide monooxygenase active on cellulose |
| FGSG_13245 | non | AA9-CBM1 | PCWDC | lytic polysaccharide monooxygenase active on cellulose |
| FGSG_02022 | non | GH72-CBM43 | PCWDC | lytic polysaccharide mono-oxygenase active on cellulose |
| FGSG_09272 | non | AA12 | PCWDC | PQQ-dependent sugar dehydrogenase |
| FGSG_04743 | non | GH78 | PCWDC | related to a-L-rhamnosidases |
| FGSG_07892 | non | GH12 | PCWDC | xyloglucanase |
| FGSG_03842 | non | GH13_1 | SDC | a-amylase |
| FGSG_17289 | non | CBM48 | SDC | a-glucan-binding protein |
| FGSG_03462 | non | GH31 | SDC | a-glucosidase |
| FGSG_09226 | non | GH31 | SDC | a-glycosidase |
| FGSG_06278 | non | GH15-CBM20 | SDC | glucoamylase |
| FGSG_11326 | non | GH15-CBM20 | SDC | glucoamylase |
| FGSG_06493 | up | CBM21 | SDC | glycogen-binding protein |
| FGSG_09704 | non | GH13_25-GH133 | SDC | glycogen-debranching enzyme |
| FGSG_04704 | non | AA13 | SDC | lytic polysaccharide monooxygenase active on starch |
| ^a^ Regulation: non= non regulated on IC vs RH, but up-regulated vs MY. ^b^ PCWDC= Plant cell wall degrading CAZyme. | | | | |
