# Supplementary material for: Infection cushions of Fusarium graminearum are fungal arsenals for wheat infection
Source: Mol Plant Pathol. 2020 Jun 23;21(8):1070–87. doi: 10.1111/mpp.12960 (PMC7368127; doi:10.1111/mpp.12960)
Supplement: Supplementary file 14 [file MPP-21-1070-s014.docx]

| **Table S7. Infection up-regulated non RH/IC plant cell wall degrading CAZymes** | | | | |
| --- | --- | --- | --- | --- |
| **Gene ID** | **Reg. ^a^** | **Family** | **Group ^b^** | **CAZy predicted function ^c^** |
| FGSG_03609 | non | GH43 | PCWDC | a-1,3-L-arabinofuranosidase |
| FGSG_07695 | non | GH43 | PCWDC | a-1,3-L-arabinofuranosidase |
| FGSG_11049 | non | CE1 | PCWDC | acetylxylan esterase |
| FGSG_11169 | non | GH27-CBM35 | PCWDC | a-galactosidase / b-L-arabinopyranosidase |
| FGSG_03629 | non | GH67 | PCWDC | a-glucuronidase |
| FGSG_03883 | non | GH115 | PCWDC | a-glucuronidase |
| FGSG_03003 | non | GH43 | PCWDC | a-L-arabinofuranosidase |
| FGSG_03054 | non | GH93 | PCWDC | a-L-arabinofuranosidase |
| FGSG_03598 | non | GH93 | PCWDC | a-L-arabinofuranosidase |
| FGSG_06463 | non | GH51 | PCWDC | a-L-arabinofuranosidase |
| FGSG_16051 | non | GH141 | PCWDC | a-L-fucosidase specific of RG-II chain F |
| FGSG_09073 | non | GH78 | PCWDC | a-L-rhamnosidase |
| FGSG_05824 | non | GH43-CBM35 | PCWDC | b-1,3-galactanase |
| FGSG_11066 | non | GH5_7-CBM1 | PCWDC | b-1,4-mannanase |
| FGSG_02632 | non | GH3 | PCWDC | b-glucosidase |
| FGSG_03387 | non | GH3 | PCWDC | b-glucosidase |
| FGSG_03410 | non | GH3 | PCWDC | b-glucosidase |
| FGSG_09159 | non | GH3 | PCWDC | b-glucosidase |
| FGSG_08609 | non | GH3 | PCWDC | b-glucosidase / b-xylosidase |
| FGSG_02836 | non | GH127 | PCWDC | b-L-arabinofuranosidase |
| FGSG_12551 | non | GH146 | PCWDC | b-L-arabinofuranosidase |
| FGSG_07993 | non | GH3 | PCWDC | b-xylosidase |
| FGSG_08946 | non | GH43 | PCWDC | b-xylosidase |
| FGSG_11468 | non | GH43 | PCWDC | b-xylosidase |
| FGSG_07639 | non | GH43 | PCWDC | b-xylosidase / a-L-arabinofuranosidase |
| FGSG_11494 | non | GH43 | PCWDC | b-xylosidase / a-L-arabinofuranosidase |
| FGSG_13189 | non | GH43-CBM6 | PCWDC | b-xylosidase / a-L-arabinofuranosidase |
| FGSG_13881 | non | GH43 | PCWDC | b-xylosidase / a-L-arabinofuranosidase |
| FGSG_05983 | non | AA3_1 | PCWDC | carbohydrate dehydrogenase |
| FGSG_00571 | non | GH7-CBM1 | PCWDC | cellobiohydrolase |
| FGSG_03628 | non | CBM1-GH6 | PCWDC | cellobiohydrolase |
| FGSG_09085 | non | AA8-AA3_1-CBM1 | PCWDC | cellobiose dehydrogenase |
| FGSG_08003 | non | CBM1 | PCWDC | cellulose-binding secreted enzyme |
| FGSG_02890 | non | CE5 | PCWDC | cutinase |
| FGSG_03304 | non | CE5 | PCWDC | cutinase |
| FGSG_03795 | non | CBM1-GH5_5 | PCWDC | endo-b-1,4-glucanase |
| FGSG_11037 | non | GH12 | PCWDC | endo-b-1,4-glucanase |
| FGSG_08011 | non | AA9-CBM1 | PCWDC | lytic polysaccharide monooxygenase active on cellulose |
| FGSG_16018 | non | AA9 | PCWDC | lytic polysaccharide monooxygenase active on cellulose |
| FGSG_02202 | non | AA9 | PCWDC | lytic polysaccharide mono-oxygenase active on cellulose |
| FGSG_06087 | non | AA11 | PCWDC | lytic polysaccharide mono-oxygenase active on chitin |
| FGSG_03908 | non | PL1_7 | PCWDC | pectate lyase |
| FGSG_03909 | non | PL3_2 | PCWDC | pectate lyase |
| FGSG_04864 | non | PL3_2 | PCWDC | pectate lyase |
| FGSG_07794 | non | PL1_10 | PCWDC | pectate lyase |
| FGSG_11094 | non | PL3_2 | PCWDC | pectate lyase |
| FGSG_11163 | non | PL1_7 | PCWDC | pectate lyase |
| FGSG_01607 | non | PL1_4 | PCWDC | pectin lyase |
| FGSG_03121 | non | PL1_4 | PCWDC | pectin lyase |
| FGSG_13834 | non | PL1_4-CBM1 | PCWDC | pectin lyase |
| FGSG_07533 | non | CE8 | PCWDC | pectin methylesterase |
| FGSG_03865 | non | AA12 | PCWDC | PQQ-dependent sugar dehydrogenase |
| FGSG_03905 | non | GH43 | PCWDC | related to a-L-arabinofuranosidases |
| FGSG_09821 | non | GH43 | PCWDC | related to a-L-arabinofuranosidases |
| FGSG_04848 | non | CE12 | PCWDC | rhamnogalacturonan acetylesterase |
| FGSG_00989 | non | PL4_1 | PCWDC | rhamnogalacturonan lyase |
| FGSG_11143 | non | PL4_3 | PCWDC | rhamnogalacturonan lyase |
| FGSG_01810 | non | GH33 | PCWDC | ulosonide hydrolase |
| FGSG_00165 | non | GH105 | PCWDC | unsaturated uronyl hydrolase |
| FGSG_02866 | non | GH105 | PCWDC | unsaturated uronyl hydrolase |
| FGSG_03143 | non | GH105 | PCWDC | unsaturated uronyl hydrolase |
| FGSG_03624 | non | GH11 | PCWDC | xylanase |
| FGSG_06445 | non | GH10 | PCWDC | xylanase |
| FGSG_10411 | non | GH10 | PCWDC | xylanase |
| FGSG_10999 | non | GH11 | PCWDC | xylanase |
| FGSG_11304 | non | CBM1-GH10 | PCWDC | xylanase |
| FGSG_11208 | non | GH74 | PCWDC | xyloglucanase |
| ^a^ Reg: non= non regulated on IC vs RH, up= up-regulated in RH compared to IC. ^b^ FCM= Fungal component modification, PCWDC= Plant cell wall degrading CAZymes. ^c^ *F. graminearum* PCWDC include cellulases, hemicellulases, xylanases, glucanases, pectinases and cutinases. | | | | |
