# Supplementary material for: Infection cushions of Fusarium graminearum are fungal arsenals for wheat infection
Source: Mol Plant Pathol. 2020 Jun 23;21(8):1070–87. doi: 10.1111/mpp.12960 (PMC7368127; doi:10.1111/mpp.12960)
Supplement: Supplementary file 15 [file MPP-21-1070-s015.docx]

**Table S8. Infection, IC or RH up-regulated CAZymes**

| **Gene ID** | **Reg. ^a^** | **Family** | **Group ^b^** | **CAZy predicted function** |
| --- | --- | --- | --- | --- |
| FGSG_10563 | IC-up | CBM50-CBM50 | FCM | carbohydrate-binding protein |
| FGSG_03212 | IC-up | GH18 | FCM | chitinase |
| FGSG_10561 | IC-up | CBM50-CBM50-CBM18-GH18 | FCM | chitinase |
| FGSG_02255 | IC-up | CBM50 | FCM | chitin-binding protein |
| FGSG_03002 | IC-up | GH43 | PCWDC | a-1,5-L-arabinanase |
| FGSG_07625 | IC-up | GH62 | PCWDC | a-L-arabinofuranosidase |
| FGSG_04768 | IC-up | GH16 | PCWDC | b-1,3(4)-glucanase |
| FGSG_11048 | IC-up | GH53 | PCWDC | b-1,4-galactanase |
| FGSG_04678 | IC-up | GH5_7 | PCWDC | b-1,4-mannanase |
| FGSG_03742 | IC-up | AA8-AA3_1 | PCWDC | cellobiose dehydrogenase |
| FGSG_04872 | IC-up | AA8-AA3_1 | PCWDC | cellobiose dehydrogenase |
| FGSG_01570 | IC-up | CE5 | PCWDC | cutinase |
| FGSG_02342 | IC-up | CE5 | PCWDC | cutinase |
| FGSG_03457 | IC-up | CE5 | PCWDC | cutinase |
| FGSG_10634 | IC-up | CE5 | PCWDC | cutinase |
| FGSG_01621 | IC-up | GH5_5 | PCWDC | endo-b-1,4-glucanase |
| FGSG_03918 | IC-up | GH5_22 | PCWDC | endo-b-1,4-glucanase |
| FGSG_08253 | IC-up | GH7 | PCWDC | endo-b-1,4-glucanase |
| FGSG_04060 | IC-up | EXPN-CBM63 | PCWDC | expansin |
| FGSG_11496 | IC-up | EXPN-CBM63 | PCWDC | expansin |
| FGSG_03632 | IC-up | AA9 | PCWDC | lytic polysaccharide monooxygenase active on cellulose |
| FGSG_03695 | IC-up | AA9 | PCWDC | lytic polysaccharide monooxygenase active on cellulose |
| FGSG_03968 | IC-up | AA9 | PCWDC | lytic polysaccharide monooxygenase active on cellulose |
| FGSG_06397 | IC-up | AA9 | PCWDC | lytic polysaccharide monooxygenase active on cellulose |
| FGSG_11488 | IC-up | AA9 | PCWDC | lytic polysaccharide monooxygenase active on cellulose |
| FGSG_02386 | IC-up | PL3_2 | PCWDC | pectate lyase |
| FGSG_02977 | IC-up | PL3_2 | PCWDC | pectate lyase |
| FGSG_03131 | IC-up | PL9_3 | PCWDC | pectate lyase |
| FGSG_03713 | IC-up | PL3_2 | PCWDC | pectate lyase |
| FGSG_04430 | IC-up | PL1_9 | PCWDC | pectate lyase |
| FGSG_09291 | IC-up | PL1_7 | PCWDC | pectate lyase |
| FGSG_03483 | IC-up | PL1_4 | PCWDC | pectin lyase |
| FGSG_03406 | IC-up | CE8 | PCWDC | pectin methylesterase |
| FGSG_03194 | IC-up | GH28 | PCWDC | polygalacturonase |
| FGSG_07551 | IC-up | GH28 | PCWDC | polygalacturonase |
| FGSG_11487 | IC-up | GH10 | PCWDC | xylanase |
| FGSG_15917 | IC-up | GH11 | PCWDC | xylanase |
| FGSG_05851 | IC-up | GH12 | PCWDC | xyloglucanase |
| FGSG_08907 | IC-up | GH131 | n.d. | b-glycosidase |
| FGSG_03612 | IC-up | CE16 | n.d. | esterase |
| FGSG_03867 | IC-up | CE1 | n.d. | esterase |
| FGSG_16895 | IC-up | CE3 | n.d. | esterase |
| FGSG_03898 | RH-up | AA7 | n.d. | dehydrogenase/oxidase |
| FGSG_02328 | RH-up | AA1 | n.d. | laccase |
| FGSG_15695 | RH-up | AA1_3 | n.d. | laccase |
| ^a^ Reg: IC up= IC up-regulated vs RH, RH-up= RH up-regulated vs IC. ^b^ FCM= Fungal component modification, PCWDC= Plant cell wall degrading CAZymes, n.d.= not defined. | | | | |
