# Supplementary material for: Infection cushions of Fusarium graminearum are fungal arsenals for wheat infection
Source: Mol Plant Pathol. 2020 Jun 23;21(8):1070–87. doi: 10.1111/mpp.12960 (PMC7368127; doi:10.1111/mpp.12960)
Supplement: Supplementary file 16 [file MPP-21-1070-s016.docx]

**Table S9. Effectors smaller than 200 aa and Cys content higher than 2%**

| **Nr** | **Locus** | **Description** | **IPRO** | **TMHMM** | **aa** | **Cys [%]** |
| --- | --- | --- | --- | --- | --- | --- |
| 1 | FGSG_11047 | conserved hypothetical protein | n.d. | 0 | 108 | 7.4 |
| 2 | FGSG_10784 | conserved hypothetical protein | n.d. | 0 | 110 | 9.1 |
| 3 | FGSG_12514 | hypothetical protein | n.d. | 0 | 118 | 4.2 |
| 4 | FGSG_02685 | conserved hypothetical protein | n.d. | 0 | 147 | 5.4 |
| 5 | FGSG_12214 | conserved hypothetical protein | n.d. | 0 | 96 | 10.4 |
| 6 | FGSG_04741 | conserved hypothetical protein | n.d. similar to CHE91 | 0 | 145 | 2.8 |
| 7 | FGSG_05046 | conserved hypothetical protein | n.d. | 0 | 130 | 6.2 |
| 8 | FGSG_16880 | hypothetical protein | n.d. | 0 | 191 | 4.2 |
| 9 | FGSG_04661 | conserved hypothetical protein | n.d. | 0 | 163 | 3.1 |
| 10 | FGSG_15661 | conserved hypothetical protein | n.d. | 0 | 96 | 10.4 |
| 11 | FGSG_13849 | conserved hypothetical protein | n.d. | 0 | 108 | 9.3 |
| 12 | FGSG_04805 | conserved hypothetical protein | n.d. | 0 | 126 | 5.6 |
| 13 | FGSG_15200 | hypothetical protein | n.d. | 0 | 59 | 3.4 |
| 14 | FGSG_03334 | conserved hypothetical protein | n.d. | 0 | 148 | 6.8 |
| 15 | FGSG_15136 | hypothetical protein | n.d. | 0 | 69 | 2.9 |
| 16 | FGSG_14010 | conserved hypothetical protein | n.d. | 0 | 183 | 4.4 |
| 17 | FGSG_03600 | conserved hypothetical protein | n.d. | 0 | 182 | 4.4 |
| 18 | FGSG_04583 | conserved hypothetical protein | n.d. | 0 | 148 | 2.7 |
| 19 | FGSG_15488 | hypothetical protein | n.d. | 0 | 55 | 5.5 |
| 20 | FGSG_15251 | conserved hypothetical protein | n.d. | 0 | 67 | 9.0 |
| 21 | FGSG_07807 | conserved hypothetical protein | n.d. similar to CHEC91 | 0 | 144 | 2.8 |
| 22 | FGSG_08085 | conserved hypothetical protein | n.d. | 0 | 149 | 2.7 |
| 23 | FGSG_08210 | conserved hypothetical protein | n.d. | 0 | 155 | 6.5 |
| 24 | FGSG_03295 | conserved hypothetical protein | n.d. | 0 | 193 | 3.6 |
| 25 | FGSG_13505 | conserved hypothetical protein | n.d. | 0 | 110 | 3.6 |
| 26 | FGSG_15385 | hypothetical protein | n.d. | 0 | 67 | 3.0 |
| 27 | FGSG_03156 | conserved hypothetical protein | n.d. | 0 | 141 | 2.8 |
| 28 | FGSG_06712 | conserved hypothetical protein | n.d. | 0 | 146 | 11.0 |
| 29 | FGSG_15448 | conserved hypothetical protein | n.d. | 0 | 90 | 8.9 |
| 30 | FGSG_11225 | conserved hypothetical protein | n.d. | 0 | 113 | 7.1 |
| 31 | FGSG_15437 | hypothetical protein | n.d. | 0 | 70 | 11.4 |
| 32 | FGSG_15931 | hypothetical protein | n.d. | 0 | 146 | 12.3 |
| 33 | FGSG_13097 | hypothetical protein | n.d. | 0 | 105 | 3.8 |
| 34 | FGSG_04971 | conserved hypothetical protein | n.d. | 0 | 110 | 2.7 |
| 35 | FGSG_03445 | conserved hypothetical protein | n.d. | 0 | 147 | 2.7 |
| 36 | FGSG_10598 | conserved hypothetical protein | n.d. | 0 | 199 | 2.0 |
| 37 | FGSG_13926 | hypothetical protein | n.d. | 0 | 113 | 2.7 |
| 38 | FGSG_15135 | hypothetical protein | n.d. | 0 | 75 | 2.7 |
| 39 | FGSG_13952 | conserved hypothetical protein | n.d. | 0 | 105 | 9.5 |
| 40 | FGSG_01239 | conserved hypothetical protein | n.d. | 0 | 165 | 3.6 |
| 41 | FGSG_04372 | conserved hypothetical protein | n.d. | 0 | 188 | 2.7 |
| 42 | FGSG_16883 | hypothetical protein | n.d. | 0 | 81 | 9.9 |
| 43 | FGSG_10212 | probable SnodProt1 PRECURSOR | IPR009009 Barwin-related endoglucanase;  IPR010829 Cerato-platanin | 0 | 139 | 2.9 |
| 44 | FGSG_02378 | conserved hypothetical protein | n.d. | 0 | 117 | 6.8 |
| 45 | FGSG_13592 | conserved hypothetical protein | n.d. | 0 | 116 | 4.3 |
| 46 | FGSG_15116 | hypothetical protein | n.d. | 0 | 47 | 4.3 |
| 47 | FGSG_15333 | hypothetical protein | n.d. | 0 | 70 | 2.9 |
| 48 | FGSG_15287 | hypothetical protein | n.d. | 0 | 50 | 2.0 |
| 49 | FGSG_13720 | hypothetical protein | n.d. | 0 | 113 | 4.4 |
| 50 | FGSG_03792 | conserved hypothetical protein | n.d. | 0 | 200 | 2.0 |
| 51 | FGSG_05341 | conserved hypothetical protein | n.d. | 0 | 189 | 2.6 |
| 52 | FGSG_02181 | conserved hypothetical protein | IPR008427 Extracellular membrane protein, CFEM domain; IPR014005 Extracellular membrane protein, CFEM domain, fungi | 0 | 161 | 5.0 |
| 53 | FGSG_00114 | conserved hypothetical protein | n.d. | 0 | 99 | 6.1 |
| 54 | FGSG_13001 | hypothetical protein | n.d. | 0 | 189 | 3.7 |
| 55 | FGSG_00260 | conserved hypothetical protein | n.d. | 0 | 77 | 7.8 |
| 56 | FGSG_16366 | hypothetical protein | n.d. | 0 | 68 | 11.8 |
| 57 | FGSG_08238 | conserved hypothetical protein | n.d. | 0 | 146 | 2.7 |
| 58 | FGSG_13917 | hypothetical protein | n.d. | 0 | 100 | 3.0 |
| 59 | FGSG_12104 | hypothetical protein | n.d. | 0 | 91 | 5.5 |
| 60 | FGSG_03586 | conserved hypothetical protein | n.d. | 0 | 154 | 3.9 |
| 61 | FGSG_12393 | hypothetical protein | n.d. | 0 | 119 | 3.4 |
| 62 | FGSG_15231 | conserved hypothetical protein | n.d. | 0 | 98 | 8.2 |
| 63 | FGSG_12644 | conserved hypothetical protein | n.d. | 0 | 159 | 4.4 |
| 64 | FGSG_05056 | hypothetical protein | n.d. | 0 | 166 | 2.4 |
| 65 | FGSG_01565 | conserved hypothetical protein | n.d. | 0 | 148 | 2.0 |
| 66 | FGSG_09955 | conserved hypothetical protein | n.d. | 0 | 166 | 4.8 |
| 67 | FGSG_10554 | conserved hypothetical protein | n.d. | 0 | 199 | 5.0 |
| 68 | FGSG_12300 | hypothetical protein | n.d. | 0 | 110 | 6.4 |
| 69 | FGSG_11135 | conserved hypothetical protein | n.d. | 0 | 99 | 6.1 |
| 70 | FGSG_03130 | conserved hypothetical protein | n.d. | 0 | 137 | 4.4 |
| 71 | FGSG_07221 | hypothetical protein | n.d. | 0 | 126 | 4.0 |
| 72 | FGSG_13635 | hypothetical protein | n.d. | 0 | 110 | 3.6 |
| 73 | FGSG_07403 | hypothetical protein | n.d. | 0 | 179 | 2.2 |
| 74 | FGSG_15142 | hypothetical protein | n.d. | 0 | 91 | 8.8 |
| 75 | FGSG_05609 | hypothetical protein | n.d. | 0 | 121 | 5.0 |
| 76 | FGSG_12103 | hypothetical protein | n.d. | 0 | 178 | 3.9 |
| 77 | FGSG_02021 | hypothetical protein | n.d. | 0 | 188 | 3.2 |
| 78 | FGSG_09133 | hypothetical protein | n.d. | 0 | 143 | 4.2 |
| 79 | FGSG_06898 | hypothetical protein | n.d. | 0 | 128 | 2.3 |
| 80 | FGSG_07699 | conserved hypothetical protein | n.d. | 0 | 174 | 2.3 |
| 81 | FGSG_02840 | conserved hypothetical protein | n.d. | 0 | 189 | 5.3 |
| 82 | FGSG_02962 | conserved hypothetical protein | n.d. | 0 | 144 | 2.1 |
| 83 | FGSG_00029 | conserved hypothetical protein | IPR018392 Peptidoglycan-binding lysin domain | 0 | 178 | 6.2 |
| 84 | FGSG_11205 | probable SnodProt1 precursor | IPR009009 Barwin-related endoglucanase; IPR010829 Cerato-platanin | 0 | 140 | 3.6 |
| 85 | FGSG_08512 | hypothetical protein | n.d. | 0 | 129 | 3.1 |
| 86 | FGSG_00056 | conserved hypothetical protein | n.d. | 0 | 159 | 3.8 |
| 87 | FGSG_00111 | conserved hypothetical protein | n.d. | 0 | 132 | 3.8 |
| 88 | FGSG_00230 | conserved hypothetical protein | n.d. | 0 | 127 | 4.7 |
| 89 | FGSG_01815 | conserved hypothetical protein | n.d. | 0 | 170 | 2.4 |
| 90 | FGSG_03820 | conserved hypothetical protein | n.d. | 0 | 120 | 5.0 |
| 91 | FGSG_04646 | hypothetical protein | n.d. | 0 | 199 | 2.5 |
| 92 | FGSG_05841 | conserved hypothetical protein | n.d. | 0 | 105 | 7.6 |
| 93 | FGSG_07972 | conserved hypothetical protein | n.d. | 0 | 122 | 3.3 |
| 94 | FGSG_08087 | hypothetical protein | n.d. | 0 | 136 | 3.7 |
| 95 | FGSG_08090 | conserved hypothetical protein | n.d. | 0 | 113 | 5.3 |
| 96 | FGSG_08152 | hypothetical protein | n.d. | 0 | 137 | 2.9 |
| 97 | FGSG_09127 | conserved hypothetical protein | n.d. | 0 | 115 | 7.0 |
| 98 | FGSG_10594 | conserved hypothetical protein | n.d. | 0 | 183 | 2.2 |
| 99 | FGSG_11373 | conserved hypothetical protein | n.d. | 0 | 136 | 5.9 |
| 100 | FGSG_11418 | hypothetical protein | n.d. | 0 | 161 | 4.3 |
| 101 | FGSG_11647 | hypothetical protein | n.d. | 0 | 116 | 6.9 |
| 102 | FGSG_11759 | hypothetical protein | n.d. | 0 | 100 | 3.0 |
| 103 | FGSG_12504 | conserved hypothetical protein | n.d. | 0 | 122 | 6.6 |
| 104 | FGSG_12805 | hypothetical protein | n.d. | 0 | 101 | 4.0 |
| 105 | FGSG_13443 | conserved hypothetical protein | n.d. | 0 | 132 | 3.0 |
| 106 | FGSG_13464 | conserved hypothetical protein | n.d. | 0 | 90 | 8.9 |
| 107 | FGSG_13817 | hypothetical protein | n.d. | 0 | 137 | 2.9 |
| 108 | FGSG_13820 | conserved hypothetical protein | n.d. | 0 | 140 | 2.1 |
| 109 | FGSG_13899 | hypothetical protein | n.d. | 0 | 114 | 4.4 |
| 110 | FGSG_14017 | hypothetical protein | n.d. | 0 | 181 | 2.8 |
| 111 | FGSG_15043 | hypothetical protein | n.d. | 0 | 91 | 3.3 |
| 112 | FGSG_15048 | hypothetical protein | n.d. | 0 | 96 | 4.2 |
| 113 | FGSG_15077 | hypothetical protein | n.d. | 0 | 91 | 5.5 |
| 114 | FGSG_15164 | hypothetical protein | n.d. | 0 | 71 | 4.2 |
| 115 | FGSG_15183 | hypothetical protein | n.d. | 0 | 80 | 3.8 |
| 116 | FGSG_15194 | hypothetical protein | n.d. | 0 | 93 | 4.3 |
| 117 | FGSG_15250 | hypothetical protein | n.d. | 0 | 91 | 3.3 |
| 118 | FGSG_15254 | conserved hypothetical protein | n.d. | 0 | 68 | 11.8 |
| 119 | FGSG_15401 | hypothetical protein | n.d. | 0 | 84 | 2.4 |
| 120 | FGSG_15469 | conserved hypothetical protein | n.d. | 0 | 92 | 6.5 |
| 121 | FGSG_15636 | hypothetical protein | n.d. | 0 | 119 | 5.0 |
| 122 | FGSG_16196 | hypothetical protein | n.d. | 0 | 116 | 6.0 |
| 123 | FGSG_17388 | hypothetical protein | n.d. | 0 | 69 | 8.7 |
| 124 | FGSG_00588 | conserved hypothetical protein | IPR008427 Extracellular membrane protein, CFEM domain; IPR014005 Extracellular membrane protein, CFEM domain, fungi | 0 | 160 | 6.3 |
| 125 | FGSG_15415 | hypothetical protein | n.d. | 0 | 71 | 2.8 |
| 126 | FGSG_13882 | hypothetical protein | n.d. | 0 | 141 | 2.8 |
| 127 | FGSG_10206 | conserved hypothetical protein | n.d. | 0 | 162 | 5.6 |
| 128 | FGSG_02674 | conserved hypothetical protein | n.d. | 0 | 115 | 5.2 |
| 129 | FGSG_15111 | hypothetical protein | n.d. | 0 | 81 | 2.5 |
| 130 | FGSG_12249 | hypothetical protein | n.d. | 0 | 100 | 7.0 |
| 131 | FGSG_10585 | conserved hypothetical protein | n.d. | 0 | 167 | 10.8 |
| 132 | FGSG_02935 | conserved hypothetical protein | n.d. | 0 | 103 | 5.8 |
| 133 | FGSG_15145 | hypothetical protein | n.d. | 0 | 59 | 3.4 |
| 134 | FGSG_13679 | hypothetical protein | n.d. | 0 | 118 | 3.4 |
| 135 | FGSG_05808 | hypothetical protein | n.d. | 0 | 188 | 4.8 |
| 136 | FGSG_13669 | hypothetical protein | n.d. | 0 | 131 | 4.6 |
| 137 | FGSG_06650 | hypothetical protein | n.d. | 0 | 141 | 2.8 |
| 138 | FGSG_12405 | conserved hypothetical protein | n.d. | 0 | 182 | 2.7 |
| 139 | FGSG_00112 | conserved hypothetical protein | n.d. | 0 | 136 | 3.7 |
| 140 | FGSG_04735 | conserved hypothetical protein | n.d. | 0 | 152 | 3.9 |
| 141 | FGSG_15466 | hypothetical protein | n.d. | 0 | 95 | 4.2 |
| 142 | FGSG_13323 | hypothetical protein | n.d. | 0 | 102 | 2.9 |
| 143 | FGSG_11910 | hypothetical protein | n.d. | 0 | 148 | 2.7 |
| 144 | FGSG_10806 | hypothetical protein | n.d. | 0 | 121 | 3.3 |
| 145 | FGSG_16439 | hypothetical protein | n.d. | 0 | 170 | 7.6 |
| 146 | FGSG_12912 | hypothetical protein | n.d. | 0 | 132 | 7.6 |
| 147 | FGSG_10058 | hypothetical protein | n.d. | 0 | 100 | 3.0 |
| 148 | FGSG_03058 | conserved hypothetical protein | n.d. | 0 | 179 | 4.5 |
| 149 | FGSG_12835 | conserved hypothetical protein | n.d. | 0 | 162 | 2.5 |
| 150 | FGSG_12673 | hypothetical protein | n.d. | 0 | 116 | 3.4 |
| 151 | FGSG_04213 | conserved hypothetical protein | n.d. AltA-1 allergen analog | 0 | 166 | 3.6 |
| 152 | FGSG_07755 | conserved hypothetical protein | n.d. | 0 | 103 | 5.8 |
| 153 | FGSG_15364 | hypothetical protein | n.d. | 0 | 92 | 3.3 |
| 154 | FGSG_09357 | conserved hypothetical protein | n.d. | 0 | 141 | 4.3 |
| 155 | FGSG_12953 | hypothetical protein | n.d. | 0 | 154 | 3.9 |
| 156 | FGSG_15165 | hypothetical protein | n.d. | 0 | 98 | 2.0 |
| 157 | FGSG_15586 | hypothetical protein | n.d. | 0 | 82 | 2.4 |
| 158 | FGSG_13628 | hypothetical protein | n.d. | 0 | 106 | 5.7 |
| 159 | FGSG_08472 | conserved hypothetical protein | n.d. | 0 | 149 | 4.0 |
| 160 | FGSG_16234 | hypothetical protein | n.d. | 0 | 94 | 6.4 |
| 161 | FGSG_13412 | hypothetical protein | n.d. | 0 | 177 | 4.0 |
| 162 | FGSG_15368 | hypothetical protein | n.d. | 0 | 78 | 2.6 |
| 163 | FGSG_03581 | conserved hypothetical protein | n.d. | 0 | 198 | 2.0 |
| 164 | FGSG_11830 | hypothetical protein | n.d. | 0 | 189 | 3.7 |
| 165 | FGSG_02759 | hypothetical protein | n.d. | 0 | 125 | 2.4 |
| 166 | FGSG_12259 | hypothetical protein | n.d. | 0 | 107 | 4.7 |
| 167 | FGSG_15128 | hypothetical protein | n.d. | 0 | 92 | 2.2 |
| 168 | FGSG_09079 | hypothetical protein | n.d. | 0 | 139 | 2.9 |
| 169 | FGSG_15149 | hypothetical protein | n.d. | 0 | 52 | 5.8 |
| 170 | FGSG_13374 | hypothetical protein | n.d. | 0 | 46 | 2.2 |
| 171 | FGSG_12722 | hypothetical protein | n.d. | 0 | 143 | 5.6 |
| 172 | FGSG_06682 | conserved hypothetical protein | n.d. | 0 | 126 | 6.3 |
| 173 | FGSG_03270 | putative protein [EST hit] | n.d. | 0 | 194 | 4.1 |
| 174 | FGSG_13051 | hypothetical protein | n.d. | 0 | 120 | 3.3 |
| 175 | FGSG_04891 | hypothetical protein | n.d. | 0 | 132 | 6.1 |
| 176 | FGSG_00847 | conserved hypothetical protein | n.d. | 0 | 64 | 6.3 |
| 177 | FGSG_05943 | conserved hypothetical protein | n.d. | 0 | 93 | 2.2 |
| 178 | FGSG_12554 | conserved hypothetical protein | n.d. | 0 | 128 | 5.5 |
| 179 | FGSG_03894 | conserved hypothetical protein | n.d. | 0 | 154 | 3.9 |
| 180 | FGSG_03326 | conserved hypothetical protein | n.d. | 0 | 172 | 4.1 |
| 181 | FGSG_12882 | hypothetical protein | n.d. | 0 | 126 | 2.4 |
| 182 | FGSG_13615 | conserved hypothetical protein | n.d. | 0 | 164 | 3.0 |
| 183 | FGSG_02077 | conserved hypothetical protein | IPR008427 Extracellular membrane protein, CFEM domain; IPR014005 Extracellular membrane protein, CFEM domain, fungi | 0 | 184 | 4.3 |
| 184 | FGSG_15611 | hypothetical protein | n.d. | 0 | 53 | 3.8 |
| 185 | FGSG_10592 | conserved hypothetical protein | n.d. | 0 | 126 | 4.8 |
| 186 | FGSG_11000 | conserved hypothetical protein | n.d. | 0 | 143 | 2.1 |
| 187 | FGSG_10603 | putative protein [EST hit] | n.d. | 0 | 158 | 5.7 |
| 188 | FGSG_03599 | conserved hypothetical protein | IPR008427 Extracellular membrane protein, CFEM domain | 0 | 95 | 10.5 |
| 189 | FGSG_08142 | conserved hypothetical protein | n.d. | 0 | 177 | 11.3 |
| 190 | FGSG_01594 | conserved hypothetical protein | n.d. | 0 | 163 | 4.9 |
| 191 | FGSG_15528 | hypothetical protein | n.d. | 0 | 54 | 7.4 |
| 192 | FGSG_13330 | hypothetical protein | n.d. | 0 | 124 | 4.8 |
| 193 | FGSG_05936 | conserved hypothetical protein | n.d. | 0 | 182 | 3.8 |
| 194 | FGSG_09570 | conserved hypothetical protein | n.d. | 0 | 170 | 4.7 |
| 195 | FGSG_02961 | hypothetical protein | n.d. | 0 | 194 | 3.1 |
| 196 | FGSG_04535 | hypothetical protein | n.d. | 0 | 134 | 7.5 |
| 197 | FGSG_15175 | conserved hypothetical protein | n.d. | 0 | 91 | 4.4 |
| 198 | FGSG_04761 | hypothetical protein | n.d. | 0 | 114 | 5.3 |
| 199 | FGSG_15182 | conserved hypothetical protein | n.d. | 0 | 87 | 9.2 |
