# Supplementary material for: Infection cushions of Fusarium graminearum are fungal arsenals for wheat infection
Source: Mol Plant Pathol. 2020 Jun 23;21(8):1070–87. doi: 10.1111/mpp.12960 (PMC7368127; doi:10.1111/mpp.12960)
Supplement: Supplementary file 17 [file MPP-21-1070-s017.docx]

| **Table S10. Putative effector proteins of *F. graminearum* with known protein domains** | | | | | | | |
| --- | --- | --- | --- | --- | --- | --- | --- |
| **Domain (IPRO)** | **Domain (PFAM)** | **Total** | **Secreted and not in a cluster ^a^** | **PI** | **>Log2**  **IC vs RH** | **Gene ID ^c^** | **Ref.** |
| Extracellular membrane protein, CFEM **(IPR008427)** | CFEM (PF05730) | 20 | 9 | 1 | 1 | FGSG_02109*  FGSG_03897  FGSG_16221 | Kulkarni *et al*., 2003; Zhang *et al*., 2012 |
| Peptidase/  proteinase inhibitor I9 **(IPR009020)** | Propep_M14 (PF02244)  Pro-kuma_activ (PF09286) S8_pro-domain (PF16470) | 19 | 15 | 3 | 0 | FGSG_03315  FGSG_08012  FGSG_10595 | Jashni *et al.*, 2015; Rooney *et al*., 2005 |
| LysM domain **(IPR018392)** | LysM (PF01476) | 10 | 7 (6) **^b^** | 4 | 3 | FGSG_00033*  FGSG_02255*  FGSG_06087  FGSG_10563 | Mentlak *et al*., 2012; Takahara *et al*., 2016 |
| Cerato-platanin **(IPR010829)** | Cerato-platanin (PF07249) | 5 | 5 | 0 | 0 | FGSG_03971  FGSG_04471  FGSG_10212  FGSG_11205  FGSG_17103 | Frías *et al*., 2011; Quarantin *et al*., 2016 |
| Necrosis inducing protein NPP1 **(IPR008701)** | NPP1 (PF05630) | 4 | 4 | 0 | 0 | FGSG_03394  FGSG_06017  FGSG_07787  FGSG_11493 | Kleemann *et al*., 2012 |
| Fungal lipase-like domain **(IPR002921)** | Lipase_3 (PF01764) | 8 | 3 | 0 | 0 | FGSG_01240  FGSG_04818  FGSG_05906 | Blümke *et al*., 2014 |
| Alt-A allergen analog  **(without- IPRO)** | Alt-A1 (PF16541) | 1 | 1 | 0 | 0 | FGSG_04213 | Chruszcz *et al*., 2012; Lu and Edwards, 2016; Zhang *et al*., 2018 |
| Chorismate mutase, type II (**IPR020822)** | CM_2 (PF01817) | 2 | 0 | 0 | 0 | FGSG_11442  FGSG_17718 | Djamei *et al*., 2011 |
| ^a^ Only secreted proteins, smaller than 1000 aa, not belonging to a cluster were considered in the final effector protein list. ^b^ One bigger than 1000 aa. ^c^ Most relevant genes of the family. * Genes identified as pathogen associated proteins in Sperschneider *et al*., 2013. | | | | | | | |
