# Supplementary material for: Infection cushions of Fusarium graminearum are fungal arsenals for wheat infection
Source: Mol Plant Pathol. 2020 Jun 23;21(8):1070–87. doi: 10.1111/mpp.12960 (PMC7368127; doi:10.1111/mpp.12960)
Supplement: Supplementary file 18 [file MPP-21-1070-s018.docx]

**Table S11. Unknown effectors up-regulated during palea infection.**

| **Nr** | **Gene ID ^a^** | **Length ^b^**  **(aa)** | **Cysteins** | **Cys (%)** | **Secretion ^c^** | **Regulation ^d^**  **>Log_2_ (IC vs RH)** | **Specificity ^e^** |
| --- | --- | --- | --- | --- | --- | --- | --- |
| 1 | FGSG_00487 | 267 | 2 | 0.7 | CS | non | fungal |
| 2 | FGSG_00602 | 305 | 18 | 5.9 | CS | down | conserved |
| 3 | FGSG_01771 | 202 | 6 | 3.0 | CS | non | fungal |
| 4 | FGSG_02293* | 486 | 3 | 0.6 | CS | non | Fusarium |
| 5 | FGSG_02685 | 147 | 8 | 5.4 | CS | **up** | fungal |
| 6 | FGSG_02903* | 248 | 18 | 7.3 | CS | non | conserved |
| 7 | FGSG_03046* | 181 | 0 | 0.0 | CS | down | Fusarium |
| 8 | FGSG_03096* | 204 | 1 | 0.5 | CS | **up** | *F. graminearum* |
| 9 | FGSG_03156 | 141 | 4 | 2.8 | CS | non | conserved |
| 10 | FGSG_03166* | 187 | 3 | 1.6 | CS | **up** | fungal |
| 11 | FGSG_03178* | 595 | 1 | 0.2 | CS | non | conserved |
| 12 | FGSG_03295 | 193 | 7 | 3.6 | CS | non | conserved |
| 13 | FGSG_03334 | 148 | 10 | 6.8 | CS | **up** | conserved |
| 14 | FGSG_03445 | 147 | 4 | 2.7 | CS | non | fungal |
| 15 | FGSG_03454 | 163 | 0 | 0.0 | CS | **up** | conserved |
| 16 | FGSG_03500 | 230 | 1 | 0.4 | NS | non | fungal |
| 17 | FGSG_03518 | 714 | 8 | 1.1 | CS | non | conserved |
| 18 | FGSG_03584 | 176 | 3 | 1.7 | CS | non | conserved |
| 19 | FGSG_03585* | 250 | 0 | 0.0 | CS | non | Fusarium |
| 20 | FGSG_03600 | 182 | 8 | 4.4 | CS | non | fungal |
| 21 | FGSG_03784 | 230 | 4 | 1.7 | CS | **up** | fungal |
| 22 | FGSG_04231 | 282 | 8 | 2.8 | CS | non | fungal |
| 23 | FGSG_04372* | 188 | 5 | 2.7 | CS | non | fungal |
| 24 | FGSG_04490 | 163 | 0 | 0.0 | CS | non | fungal |
| 25 | FGSG_04583 | 148 | 4 | 2.7 | CS | non | fungal |
| 26 | FGSG_04661* | 163 | 5 | 3.1 | CS | **up** | Fusarium |
| 27 | FGSG_04741 | 145 | 4 | 2.8 | NS | non | fungal |
| 28 | FGSG_04744 | 553 | 9 | 1.6 | CS | non | fungal |
| 29 | FGSG_04805* | 126 | 7 | 5.6 | CS | non | Fusarium |
| 30 | FGSG_04971 | 110 | 3 | 2.7 | CS | non | Fusarium |
| 31 | FGSG_05046 | 130 | 8 | 6.2 | CS | **up** | fungal |
| 32 | FGSG_05803 | 304 | 10 | 3.3 | CS | **up** | fungal |
| 33 | FGSG_06466 | 239 | 4 | 1.7 | CS | **up** | conserved |
| 34 | FGSG_06497 | 372 | 5 | 1.3 | CS | non | fungal |
| 35 | FGSG_06564 | 228 | 5 | 2.2 | CS | non | fungal |
| 36 | FGSG_06712* | 146 | 16 | 11.0 | CS | non | fungal |
| 37 | FGSG_07026* | 67 | 0 | 0.0 | CS | **up** | fungal |
| 38 | FGSG_07560 | 432 | 2 | 0.5 | CS | **up** | conserved |
| 39 | FGSG_07670* | 259 | 3 | 1.2 | CS | non | fungal |
| 40 | FGSG_07807 | 144 | 4 | 2.8 | CS | non | fungal |
| 41 | FGSG_08048 | 466 | 6 | 1.3 | CS | non | conserved |
| 42 | FGSG_08085 | 149 | 4 | 2.7 | CS | non | Fusarium |
| 43 | FGSG_08146 | 426 | 7 | 1.6 | CS | non | fungal |
| 44 | FGSG_08210* | 155 | 10 | 6.5 | CS | non | fungal |
| 45 | FGSG_08387 | 188 | 2 | 1.1 | CS | **up** | fungal |
| 46 | FGSG_09094* | 172 | 0 | 0.0 | CS | non | conserved |
| 47 | FGSG_09886 | 261 | 2 | 0.8 | CS | non | fungal |
| 48 | FGSG_10357* | 871 | 13 | 1.5 | CS | **up** | conserved |
| 49 | FGSG_10562 | 286 | 9 | 3.1 | CS | **up** | fungal |
| 50 | FGSG_10598* | 199 | 4 | 2.0 | CS | non | fungal |
| 51 | FGSG_10784* | 110 | 10 | 9.1 | CS | **up** | Fusarium |
| 52 | FGSG_11033* | 108 | 2 | 1.9 | CS | non | Fusarium |
| 53 | FGSG_11046 | 231 | 4 | 1.7 | CS | **up** | conserved |
| 54 | FGSG_11047 | 108 | 8 | 7.4 | CS | **up** | fungal |
| 55 | FGSG_11101 | 474 | 15 | 3.2 | CS | **up** | conserved |
| 56 | FGSG_11225* | 113 | 8 | 7.1 | CS | non | fungal |
| 57 | FGSG_11515* | 171 | 1 | 0.6 | CS | non | conserved |
| 58 | FGSG_12081 | 139 | 2 | 1.4 | CS | non | Fusarium |
| 59 | FGSG_12214 | 96 | 10 | 10.4 | CS | non | conserved |
| 60 | FGSG_12514* | 118 | 5 | 4.2 | CS | **up** | Fusarium |
| 61 | FGSG_13097* | 105 | 4 | 3.8 | NS | non | *F. graminearum* |
| 62 | FGSG_13505 | 110 | 4 | 3.6 | CS | non | fungal |
| 63 | FGSG_13515* | 122 | 1 | 0.8 | CS | non | *F. graminearum* |
| 64 | FGSG_13592* | 116 | 5 | 4.3 | CS | non | *F. graminearum* |
| 65 | FGSG_13849* | 108 | 10 | 9.3 | CS | **up** | Fusarium |
| 66 | FGSG_13926* | 113 | 3 | 2.7 | NS | **up** | *F. graminearum* |
| 67 | FGSG_14010 | 183 | 8 | 4.4 | CS | **up** | conserved |
| 68 | FGSG_15136 | 69 | 2 | 2.9 | CS | **up** | *F. graminearum* |
| 69 | FGSG_15198 | 64 | 0 | 0.0 | NS | non | *F. graminearum* |
| 70 | FGSG_15200 | 59 | 2 | 3.4 | NS | non | *F. graminearum* |
| 71 | FGSG_15251 | 67 | 6 | 9.0 | CS | **up** | *F. graminearum* |
| 72 | FGSG_15385 | 67 | 2 | 3.0 | NS | non | *F. graminearum* |
| 73 | FGSG_15437 | 70 | 8 | 11.4 | CS | non | Fusarium |
| 74 | FGSG_15448 | 90 | 8 | 8.9 | CS | **up** | fungal |
| 75 | FGSG_15488 | 55 | 3 | 5.5 | CS | **up** | conserved |
| 76 | FGSG_15661 | 96 | 10 | 10.4 | CS | non | Fusarium |
| 77 | FGSG_15931 | 146 | 18 | 12.3 | CS | non | fungal |
| 78 | FGSG_16880 | 191 | 8 | 4.2 | CS | **up** | fungal |
| 79 | FGSG_17388 | 69 | 6 | 8.7 | CS | down | conserved |
| 80 | FGSG_17621 | 308 | 1 | 0.3 | CS | **up** | fungal |

Secreted proteins without IPRO domain, not belonging to a cluster and less than 1000 aa. ^a^ Gene ID from the FGDB database (<ftp://ftpmips.gsf.de/fungi/Fusarium/F_graminearum_PH1_v32/>). ^b^ Proteins length with signal peptide. ^c^ CS: Classic secretion, NS: Non classic secretion. ^d^ Regulation: non- Log_2_ FC between (-2, +2); down- Log_2_ FC < -2; up- Log_2_ FC > +2. ^e^ Taxonomic distribution: Conserved, conserved outside fungal kingdom; fungal, present only in fungi; Fusarium, conserved among *Fusarium* species; *F. graminearum*= present only in *Fusarium graminearum* strains*.* * Genes identified as pathogen associated proteins in Sperschneider *et al*., 2013.
