# Supplementary material for: Infection cushions of Fusarium graminearum are fungal arsenals for wheat infection
Source: Mol Plant Pathol. 2020 Jun 23;21(8):1070–87. doi: 10.1111/mpp.12960 (PMC7368127; doi:10.1111/mpp.12960)
Supplement: Supplementary file 19 [file MPP-21-1070-s019.docx]

Table S12. Aurofusarin bioactivity assay of *F. graminearum* wild type and *∆pks12* mutant.

|  | OD_595_ after 16h incubation with extracts of: | | |
| --- | --- | --- | --- |
| **Yeast and bacterial strains** | **WT** | ***∆pks12*** | **buffer*** |
| *Bacillus subtilis* | 0.000 | 1.960 | 1.910 |
| *Escherichia coli* | 2.412 | 3.360 | 3.163 |
| *Micrococcus luteus* | 0.037 | 2.301 | 2.213 |
| *Pseudomonas aeruginosa* | 2.009 | 2.127 | 2.230 |
| *Candida albicans* | 0.004 | 5.254 | 5.148 |
| *Candida parapsilosis* | 2.670 | 4.830 | 5.124 |
| *Pichia pastoris* | 0.542 | 3.556 | 3.276 |
| *Saccharomyces cerevisiae* | 1.966 | 3.896 | 3.963 |
| *Geotrichum candidum* | 2.966 | 3.466 | 3.702 |
| *Pseudomonas fluorescens* | 2.404 | 2,284 | 2.296 |
| *Janthinobacteria* HH102 | 1.413 | 1.377 | 1.033 |
| *Rhizobium* sp. NG234 | 0.870 | 0.849 | 0.917 |
| Dry weight [mg] after 40h incubation with extracts of: | | |  |
| **Fungal strains** | **WT** | ***∆pks12*** | **buffer*** |
| *Fusarium graminearum* (conidia) | 17 | 16 | 18 |
| *Fusarium graminearum* (mycelium) | 14 | 15 | 13 |
| *Nectria haematococca* (conidia) | 15 | 16 | 15 |
| *Pyrenophora teres* (mycelium) | 0 | 9.5 | 8 |

*Phosphate buffer was used as a negative control.
