# Supplementary material for: Infection cushions of Fusarium graminearum are fungal arsenals for wheat infection
Source: Mol Plant Pathol. 2020 Jun 23;21(8):1070–87. doi: 10.1111/mpp.12960 (PMC7368127; doi:10.1111/mpp.12960)
Supplement: Supplementary file 20 [file MPP-21-1070-s020.docx]

Table S13. Statistics for β-tubulin, cofilin and ubiquitin genes.

| **CP data of housekeeping Genes by BEST KEEPER** | | | |
| --- | --- | --- | --- |
|  | **β-tubulin** | **cofilin** | **ubiquitin** |
| n | 162 | 162 | 162 |
| geo Mean [CP] | 12.07 | 9.53 | 10 |
| AR Mean [CP] | 12.27 | 9.65 | 10.09 |
| min [CP] | 8 | 7 | 7 |
| max [CP] | 18 | 12 | 12 |
| std dev [+/- CP] | 1.9 | 1.3 | 1.15 |
| CV [% CP] | 15.49 | 13.45 | 11.41 |
| min [x-fold] | -16.84 | -5.79 | -8.01 |
| max [x-fold] | 60.82 | 5.53 | 3.99 |
| std dev [+/- x-fold] | 3.73 | 2.46 | 2.22 |
| Genes were used as internal control based on their values of CP and results provided by software BestKeeper | | | |
