# Supplementary material for: Infection cushions of Fusarium graminearum are fungal arsenals for wheat infection
Source: Mol Plant Pathol. 2020 Jun 23;21(8):1070–87. doi: 10.1111/mpp.12960 (PMC7368127; doi:10.1111/mpp.12960)
Supplement: Supplementary file 21 [file MPP-21-1070-s021.docx]

Table S14. Comparison of gene stability of three housekeeping genes for internal control in RT-qPCR.

| **Method** | **β-tubulin** | **cofilin** | **ubiquitin** | **References** |
| --- | --- | --- | --- | --- |
| BestKeeper | 1.901 | 1.299 | 1.151 | Pfaffl *et al*., 2004 |
| normFinder | 1.109 | 0.275 | 0.686 | Andersen *et al*., 2004 |
| Genorm | 1.062 | 0.739 | 0.739 | Vandesompele *et al*., 2002 |
| Delta CT | 1.223 | 0.941 | 1.021 | Silver *et al*., 2006 |
