# Supplementary material for: Infection cushions of Fusarium graminearum are fungal arsenals for wheat infection
Source: Mol Plant Pathol. 2020 Jun 23;21(8):1070–87. doi: 10.1111/mpp.12960 (PMC7368127; doi:10.1111/mpp.12960)
Supplement: Supplementary file 22 [file MPP-21-1070-s022.docx]

**Table S15. Primers used in this study.**

| **Primers used for *FgPE1* constructs** | | | |
| --- | --- | --- | --- |
| **Application** | **Primer** | **Sequence (5´-> 3´)** | **Product length (bp)** |
| (FGSG_04213) FgPE1 deletion construct | 1F-FgPE1-Vect | tgtaaaacgacggccagtgagcgcgcgtTCGTATGTCTCCTTGTTGGTG | 949 |
|  | 2R- FgPE1-Hyg | caaaggaatagagtagatgccgaccgaacTGGATAGATGAAGGAGTCGAG |  |
|  | 3F- FgPE1-Hyg | agtcaatgctacatcacccacctcgctcAGTCTTGTAAGAGGCGGAACC | 803 |
|  | 4R- FgPE1-vect | ctctagaactagtggatcccccgggctgAGGCGTGGAATGAGACAGAC |  |
|  | 12F- FgPE1 | GTACAGACTATAACCAATGGCG (outside construct to check deletion) | 2541 |
|  | 13R- FgPE1 | CCACTAAAACTAAGACTGAGGC (outside construct to check deletion) |  |
| P_FgPE1_:FgPE1:mCherry Localization construct | 1F-FgPE1-Vect | tgtaaaacgacggccagtgagcgcgcgtTCGTATGTCTCCTTGTTGGTG | 1469 |
|  | 5R- FgPE1-GA | ggcaccggctccagcgcctgcaccagctccCAAGACTGCGCGCATCTCGA |  |
| P_FgPE1_:mCherry expression construct | 1F-FgPE1-Vect | tgtaaaacgacggccagtgagcgcgcgtTCGTATGTCTCCTTGTTGGTG | 971 |
|  | 14R- FgPE1-mCh | gttatcctcctcgcccttgctcaccatATTGATGGTTGATGTAGAAGGAT |  |
| FgPE1 Southern probe 2 | 8F- FgPE1 | CTTTTGCGGCAGCCTTTTCC | 413 |
|  | 9R- FgPE1 | TCCCATAAAGATCACACCGCT |  |
| FgPE1 Southern probe 1 | 10F- FgPE1 | CTACAACGTCCAGAAGAGCAC | 358 |
|  | 11R- FgPE1 | CTGCGCGCATCTCGAGAAC |  |
| Hygromicin resistance cassette | 1F-Hyg | GAGCGAGGTGGGTGATGTAG | 1740 |
|  | 2R-Hyg | CGGTCGGCATCTACTCTATTC |  |
|  | 3F-Hyg | CTGGCAAACTGTGATGGACGA (for sequencing) |  |
|  | 4R-Hyg-vect | cgctctagaactagtggatcccccgggctgCTCCCATATGGTCGACCTGC (for expression construct) |  |
| Geneticin resistance cassette | 1F-NptII | GCCAGTTGTTCCCAGTGATCT | 2323 |
|  | 2R-NptII-vect | gctctagaactagtggatcccccgggctgGCGAGGTCCAATGCATTAATG |  |
| mCherry ORF | 1F- mCh | ATGGTGAGCAAGGGCGAGGAG | 708 |
|  | 14F-mCh-GA | ggaggtgcaggcgctggagccggtgccATGGTGAGCAAGGGCGAGGAGG |  |
|  | 18R-mCh-Hyg | agtcaatgctacatcacccacctcgctcTTACTTGTACAGCTCGTCCATG |  |
|  | 19R-mCh-NptII | ggtaggccgaataacttgcacaaattggTTACTTGTACAGCTCGTCCATG |  |
| **F**: forward primer; **R**: reverse primer. **Lowercase**: overhangs for fusion. | | | |
| **Primers used for transcriptome validation** | | | |
| **Gene** | **Primer** | **Sequence (5’ -> 3’)** | **Product length (bp)** |
| β-tubulin (FGSG_06611) | JB_06611_fw | CGCATACCACGGAACTTCAG | 105 |
|  | JB_06611_rev | TCAAGATCGACCAGAACAGCA |  |
| Cofilin (FGSG_06245) | Fg_6245_fw | TGGTATCCGAAACAAGATCACCT | 160 |
|  | Fg_6245_rv | GTCGTATTCGATGTCATCAGTGTC |  |
| Ubiquitin (FGSG_10805) | Fg_10805_fw | CTTCACTACACGCATCTACC | 112 |
|  | Fg_10805_fw | GGACAGAAGAACTTTAGAGATGG |  |
| FgTRI5 (FGSG_03537) | AGL_qRT-tri5_fw | TTTTTGAGGGATGCTGGATTGA | 121 |
|  | AGL_qRT-tri5_rv | GCCATAGAGAAGCCCCAACAC |  |
| FgPKS12 (FGSG_02324) | AGL_qRT-pks12_fw | AACTGTCATCAGACTACGCC | 198 |
|  | AGL_qRT-pks12_rv | TATGTCTCCATAAACACCAACC |  |
| FgGTA1 (FGSG_05554) | JB_05554_gta1_fw | CAAGACCAAGAGCCACATGG | 142 |
|  | JB_05554_gta1_rev | AATCTGAGCGAAGACGTCGA |  |
| FgGTA2 (FGSG_06751) | JB_06751_gta2_fw | CTTCTTTCACCGAGAACGCC | 146 |
|  | JB_06751_gta2_rev | ATCGACGATGTAGTTATCGTGAG |  |
| FgPE1 (FGSG_04213) | 10F- FgPE1 | CTACAACGTCCAGAAGAGCAC | 358 |
|  | 11R- FgPE1 | CTGCGCGCATCTCGAGAAC |  |
