# Supplementary material for: Infection cushions of Fusarium graminearum are fungal arsenals for wheat infection
Source: Mol Plant Pathol. 2020 Jun 23;21(8):1070–87. doi: 10.1111/mpp.12960 (PMC7368127; doi:10.1111/mpp.12960)
Supplement: Supplementary file 23 [file MPP-21-1070-s023.docx]

**Table S16.** **Plasmids used or generated in this study.**

| **Name** | **Use** | **Restriction enzymes^a^** | **References** |
| --- | --- | --- | --- |
| pRS426 | Yeast cloning method |  | Christianson *et al*., 1992 |
| pGEM-Hyg | Hygromycin (Hyg) selectable marker |  | Maier *et al*., 2005 |
| pII99 | Geneticin (NptII) selectable marker |  | Beck *et al*., 1982 |
| pNRI | Nourseothricin (NatI) selectable marker |  | Malonek *et al*., 2004 |
| pALM-FgPE1::Hyg | FgEP1 (FGSG_04213) deletion construct | *Pvu*II | This study |
| pALM- FgPE1_Prom_::mCherry::Hyg | PromFgPE1:mCherry expression construct | *Bss*HII | This study |
| pALM- FgPE1_Prom_::FgPE1::mCherry::NptII | FgPE1 localization construct | *Pvu*I and *Sac*I | This study |

^a^ Restriction enzymes to linearize the vector or excise the construct and use for *F. graminearum* transformation.
