# Supplementary material for: Infection cushions of Fusarium graminearum are fungal arsenals for wheat infection
Source: Mol Plant Pathol. 2020 Jun 23;21(8):1070–87. doi: 10.1111/mpp.12960 (PMC7368127; doi:10.1111/mpp.12960)
Supplement: Supplementary file 30 [file MPP-21-1070-s030.docx]

**Detailed experimental procedures.**

**Fungal growth and conidia production.** *Fusarium graminearum* wild type Fg-8/1 (WT) (Miedaner *et al*., 2000), WT strain expressing cytosolic GFP constitutively (WT-GFP) (Jansen *et al*., 2005), trichodiene synthase deficient mutant expressing GFP constitutively (∆tri5-GFP) (36- Jansen et al., 2005), polyketide synthase deletion mutant (∆pks12) (Malz *et al*., 2005), TRI5Prom:GFP with constitutive DsRED reporter strain (Ilgen *et al*., 2009). WT and mutants used and produced in this study were grown, cultured and transformed as described before (Jansen *et al*., 2005). Complete media (CM) was prepared according to (Leach *et al*., 1982). Liquid wheat media (WM) was prepared according to (Quarantin *et al*., 2019). For biological assays, cultures were prepared with a conidial suspension of 1×10^5^ conidia/mL in a 150 mL flask containing 20 mL liquid media (CM or WM) for the indicated time on a rotary shaker at 150 rpm and 26 °C. Macroconidia of WT and mutant strains were produced in liquid WM during 4 to 7 days.

**Laser capture microdissection (LCM).** Paleae containing RH and IC were prepared by cutting off their upper and lower ends and immediately transferred to ethanol absolute on ice according to previous studies (Clément-Ziza *et al*., 2008; Goldsworthy *et al*., 1999). After collection of 90‑150 specimens, ethanol was removed and samples were lyophilized for 12 h and stored at -80°C until use. RH and IC were transferred from the plant surface onto RNase-free glass slides. Glass slides were covered with 120 µL of liquid cover glass (Carl Zeiss, Göttingen, Germany), and allowed to dry for 5 min. Six palea pieces were pressed with its inoculated side on one glass slide. Remaining palea tissue was carefully removed with forceps. Identification and isolation of RH and IC was done with the inverse microscope PALM Micro Beam (Carl Zeiss, Göttingen, Germany). RH and IC were selected using the auto-LPC-function of the PALMRobo software (Version 4.3 SP2). The following settings were used: Cutting energy 32 at a focus of 63, catapulting at delta 25 of the cutting energy and a focus of -2. Selected RH and IC were catapulted upwards in an adhesive cap of a 500 µL tube (Carl Zeiss, Göttingen, Germany) and stored at -80°C. A total area of 1×10^6^ µm^²^ for runner hyphae (approx. 900 units) and a total area of 0.15×10^6^ µm^²^ for compound appressoria (approx. 450 units) were used for mRNA isolation and cDNA library construction.

**RNA extraction, amplification and cDNA library construction.** RNA extraction, amplification and cDNA library construction of IC, RH or MY, were performed according to (Lê *et al*., 2005). Briefly, the isolation of mRNA was performed with the Dynabeads® mRNA DIRECT™ Kit (Invitrogen, Dynal, Hamburg, Germany) with the following specifications: for lysis of the fungal cells, 50 µL Lysis/Binding Buffer (200 mM Tris-HCl pH 7.5, 1M LiCl, 20 mM EDTA pH 8.0, 10 mM DTT, 2% LiDS) were added to the collection tubes from LCM. The tubes were vortexed with the cap downwards for 1 min at room temperature. After washing 15 µL Dynabeads® Oligo (dT)_25_ with Lysis/Binding Buffer, the cell lysate was added to the beads. Hybridization was performed on a roller for 10 min at room temperature. The supernatant was removed and the beads/mRNA complex was washed twice with 50 µL washing buffer (10 mM Tris-HCl pH 7.5, 0.15 M LiCl, 1 mM EDTA) and twice with 50 µL first-strand buffer (50 mM Tris-HCl pH 8.3, 75 mM KCl, 3 mM MgCl_2_). The beads/mRNA complex was resuspended in 3 µL RNAse free water and used for first-strand cDNA synthesis using the SMARTer™ Pico PCR cDNA Synthesis Kit (Takara Bio Europe/Clontech, Saint-Germain-en-Laye, France) by switching mechanism at 5’ end of the RNA transcript polymerase chain reaction (SMART-PCR) (Zhu *et al*., 2001), 1 µL 3’ SMART CDS Primer II A (12 μM) was added to the 3 µL beads suspension and incubated at 72°C for 2 min. Subsequently, 6 µL Master Mix (2 µL 5x First-Strand Buffer; 0.25 µL 100 mM DTT, 1 µL 10 mM dNTP Mix, 1 µL 12 µM SMARTer II A Oligonucleotide, 0.25 µL RNase Inhibitor, 1 µL 100 U SMARTScribe Reverse Transcriptase and 0.5 µL H_2_O) were added to a total reaction volume of 10 µL. The PCR was performed at 42°C for 90 min and terminated by incubation at 72°C for 10 min. The products were chilled on ice for 10 min and processed in a long distance (LD)-PCR. The LD-PCR was performed with the Advantage®2 PCR Kit (Takara Bio Europe/Clontech, Saint-Germain-en-Laye, France).

To ensure that the synthesis still takes place in the exponential phase of cDNA amplification, the optimal cycle count of the LD-PCR was determined by two test reactions for each replicate of runner hyphae, compound appressoria or the mycelium culture. To determine the optimal cycle range between 15, 18, 21, 24, 27, 30 and 32 cycles, the first LD-PCR was prepared in 50 µL reaction volume using 1 µL template from the SMART-PCR and 49 µL Master Mix (5 µL of 10X Advantage 2 PCR Buffer, 1 µL 10 mM dNTP´s, 1µL 12µM 5’ PCR Primer II A, 1 µL 50X Advantage 2 Polymerase Mix and 41 µL H_2_O). The products were analysed by gel electrophoresis in 1.2% (w/v) agarose in 1X TAE buffer (40 mM Tris, 20 mM acetic acid, and 1 mM EDTA). The second LD-PCR was done with 0.5 µL template in a total reaction volume of 25 µL. The reaction was sampled in every cycle within the determined cycle range, which showed the strongest amplification of PCR product. The final cycle count was one cycle below the amplification stagnation or reaction saturation was reached. The remaining SMART-PCR cDNA of each sample was used for the subsequent final LD-PCR. The reaction was split into multiple 50 µL reactions, which were pooled afterwards and processed through purification and 5’ phosphorylation of cDNAs.

**Finalization of cDNA libraries (End-it-Reaction).** To provide 5´-phosphorylated, blunt-ended cDNAs, the End-It^™^ DNA End-Repair Kit (Biozym Biotech Trading GmbH, Vienna, Austria) was used according to a modified protocol. The finalization was done in two reactions with a total volume of 100 µL. For each reaction 68 µL of purified cDNA of each cell type, 5 µL 10X End-Repair Buffer, 5 µL dNTP Mix, 5 µL ATP, 1 µL End-Repair Enzyme Mix and 16 µL H_2_O were used. The reaction mixes were incubated for 45 min at room temperature and afterwards inactivated for 10 min at 70°C. The blunt-ended cDNA libraries from compound appressoria, runner hyphae and the mycelium culture were purified again with the NucleoSpin^®^ Gel and PCR Clean-up Kit as described before. The final elution of the cDNA libraries was performed twice with 25 µL per column. The cDNA of each sample was pooled and the amount of pure blunt-ended cDNA of each cell type was determined by electrophoresis in 1.2% (w/v) agarose gels.

1 µg of the final cDNA libraries of three independent replicates of runner hyphae and compound appressoria, respectively, were sent for RNA-Seq analysis by the workgroup of Prof. Dr. Adam Grundhoff (Heinrich-Pette-Institute, Virus Genomics, Hamburg, Germany). The RNA-Seq of the mycelium culture was performed with 1 µg each of three replicates by the Beijing Genomics Institute Hong Kong (Shenzhen, China).

**Annotations and databases used.** The transcriptome data discussed in this publication have been deposited in NCBI's Sequence Read Archive (SRA), <https://www.ncbi.nlm.nih.gov/sra/>; SUB3191581 (Edgar *et al*., 2002). The reference genome of *Fusarium graminearum* PH1 database FGDB: <ftp://ftpmips.gsf.de/fungi/Fusarium/F_graminearum_PH1_v32/> was used to map the cDNA libraries constructed (Wong *et al*., 2010). Genes were manually grouped into the following gene families (<https://ghr.nlm.nih.gov/primer/genefamily/genefamilies>): secondary metabolite biosynthesis gene clusters (SMC), dehydrogenases (DH), transmembrane receptors (TMR), putative effector proteins (PE), transcription factors (TF), histone modifying proteins (HM), protein kinases/phosphatases (PK), carbohydrate-active enzymes (CAZymes), and genes involved in reactive oxygen species metabolism (ROS), according to their annotation, conserved functional domains, previously published information, and phylogenetic relationship (for details see Data set S2). Functional predictions of CAZymes were made by blasting each sequence against a sequence library made only with experimentally characterized enzymes. In a few instances, when the distance with the characterized enzyme was too large for a reliable precise function, the function was downgraded to "related to" or just "b-glycosidase" to indicate that the precise substrate could not be predicted (Lombard *et al*., 2013); <http://www.cazy.org/>. To select *F. graminearum* putative effector proteins (PE) with known domains, we used the IPRO or PFAM predicted domains. For the identification and evaluation of taxonomic distribution of known and unknown *F. graminearum* effectors the reference genome database (FGDB) (http://pedant.helmholtz-muenchen.de/pedant3htmlview/pedant3view?Method=start_method&Db=p3_p13839_Fus_grami_v32) and NCBI-blastp were used (<https://blast.ncbi.nlm.nih.gov/Blast.cgi?PAGE=Proteins>). Proteins that returned no BLAST hit except for *F. graminearum* were called “*F. graminearum* specific”, those with orthologues in other Fusaria were called “Fusarium specific” and those with BLAST hits across several fungal genera were called “fungal specific” effectors. Secondary metabolite gene cluster (SMC) were annotated according to Sieber *et al*. (2014). To estimate transcriptional regulation of complete clusters, we considered the Log_2_ FC and FPKM values of four types of signature enzymes (polyketide synthases, non-ribosomal peptide synthases, terpene synthases, and prenyltransferases), as well as five tailoring enzyme classes (methyltransferases, acyltransferases, oxidoreductases, glycosyltransferases and cytochrome P450s). In addition, we took into account transcription factors and transporter enzymes that might contribute to regulation and secretion of the metabolite. Gene clusters were defined as not expressed when at least 30 % of genes in the cluster and/or the key gene were not expressed (FPKM= 0.0). For TMR, proteins were selected that possess seven membrane-spanning domains and sequence homology to G-protein-coupled receptors. ROS-related genes were selected according to the biochemical pathways they are involved in and involve components of NADPH oxidase (NOX) complexes (*Fgbem1*, *FgnoxR*, *FgnoxA*, *FgnoxB*, *FgnoxC*), cupredoxines, dehydrogenases, monooxygenases, superoxide dismutases, catalases, thioredoxin reductases, cytochrome-b5 reductases, glutaredoxins, glutathione peroxidases, glutathione-S-transferases, peroxisomal proteins, and transcriptional regulators (*e.g.* *Fgap1*, *Fgskn7*, *FgOS-2*, *Fgatf1*). If a biochemical reaction catalysed by an enzyme involves in formation of ROS, it was included to the ROS gene family. Furthermore, published data on ROS-related enzymes and the prediction of conserved functional domains were taken into account. A list of putative *F. graminearum* PK, HM und DH have been retrieved from fungidb (<http://fungidb.org/>). The list of TF was obtained from Son *et al*., (2011). At least 7942 proteins (57%) lack adequate functional annotation, conserved domains or sufficient sequence similarities to known members of gene families. Those proteins, commonly referred to as “Unknown” or “hypothetical” proteins, have usually not been included into any of the depicted gene families, except they have been functionally characterized and described as being involved in processes related to one or more gene families.

**Validation of RNAseq data by quantitative real time PCR.** Validated genes were FgTRI5 (FGSG_03537), FgPKS12 (polyketide synthase 12; FGSG_02324), FgPE1 (FGSG_04213; putative effector 1) and two GABA-aminotransferases (FgGTA1, FGSG_05554; FgGTA2, FGSG_06751). The same RNA samples used to produce the cDNA for the transcripts profiling were used for the RT-qPCR analyses. The RT-qPCR reactions were carried out using 1µL of cDNA (1.5 ng/µL) as a template, primers shown in Table S15 and the master mix of intercalating fluorescent dye LightCycler® 480 SYBR Green I Master (Roche, Germany) in a Rotor-Gene Q (Qiagen). The PCR program was as follows: denaturation for 2 min at 95 °C, 40 cycles of denaturation at 94 °C for 30 s, annealing at 58 °C for 30 s and extension at 72 °C for 15 s, followed by a melting curve analysis. Ct values were obtained from 3 technical replicates performed on samples from three independent biological replicates for each structure analysed. For relative expression analysis the tool REST (Relative Expression Software Tool) was used (Pfaffl *et al*., 2002). For normalisation of gene-expression the housekeeping genes cofilin (FGSG_06245) and ubiquitin (FGSG_10805) were used. For evaluation of housekeeping genes, expression of a collection of 162 CT-values of 3 housekeeping genes (β-tubulin, cofilin and ubiquitin), obtained under different conditions, was made and used to evaluated gene expression stability using the web-based comprehensive tool “Ref Finder” (Andersen *et al*., 2004; Pfaffl *et al*., 2004; Silver *et al*., 2006; Vandesompele *et al*., 2002). This tool uses 5 different programs and for every program either cofilin or ubiquitin were recommended as housekeeping genes (Table S13 and Table S14).

**Generation of** **knock-out, expression and localization constructs for FgPE1 mutants.** All plasmids were constructed using the yeast recombination method (Colot *et al*., 2006), and the pRS426 background plasmid (112- Christianson *et al*., 1992). and the pRS426 background plasmid (Christianson *et al*., 1992). Amplification of 5´ and 3´ flanks of the genes of interest was performed using primers shown in Table S15 and genomic DNA extracted from the WT strain. Hygromycin and geneticin resistance cassettes were amplified from pGEM-Hyg (Maier *et al*., 2005) and pII99 (Beck *et al*., 1982) via PCR using the respective primers (Table S15). The final constructs were excised with the respective restriction enzymes (Table S16) and used to transform *F. graminearum* WT strain. Deletion of FgPE1 was confirmed by Southern blot using two different probes amplified with primers shown in Table S15. At least two independent deletion mutants were generated and examined for each gene of interest. The expression construct pALM-FgPE1_Prom_::mCherry::Hyg was generated by fusing the native promoter of the FgPE1 gene (971 bp) with the fluorescence reporter gene mCherry (711 bp) and adding the hygromycin resistance cassette (1742 bp) into the pRS426 vector using the yeast recombination method (Colot *et al*., 2006). All fragments were amplified using Q5^®^ High-Fidelity DNA Polymerase (New Englad Biolabs), genomic DNA extracted from WT or respective plasmid as template and specific primers listed in the Table S15. The final construct was excised with *Bss* HII restriction enzyme and used to transform *F. graminearum* WT strain. Similarly, the localization construct pALM-FgPE1_Prom_::FgPE1::mCherry::NptII was generated by fusing the native promoter and ORF of the FgPE1 gene without stop codon (1469 bp) with the fluorescence reporter gene mCherry (711 bp) and adding the geneticin resistance (NptII) cassette (2281 bp). The final construct was excised with *Pvu* I and *Sac* I restriction enzymes and used to transform the WT-GFP like strain constitutively expressing the reporter gene GFP (Jasen *et al*., 2005).

**Virulence assay: wheat spikes point inoculation and wheat palea infection.** Virulence assays where prepared according to (Boenisch and Schäfer, 2011; Frandsen *et al*., 2006). Ten spikes of the susceptible spring wheat cultivar Nandu were used for each analysed strain. Detached wheat palea infection assays were prepared as mentioned above in section “Preparation of wheat infected tissue for laser capture microdissection”. Four Petri dishes containing 8 biological replicates of palea were prepared and each petri dish represented one independent experiment.

**Quantification of fungal material within inoculated wheat spikes using quantitative Real Time-PCR.** Genomic DNA of inoculated wheat spikes was isolated using the CTAB method (Voigt *et al*., 2007). The amount of fungal DNA was determined using SYBR-green based qPCR with primers for β-tubulin (FGSG_06611). The Ct values for quantification were obtained from 3 technical replicates performed on samples from 3 independent gDNA isolations, which refer to 10 biological replicates. For calculation of the amount of fungal gDNA, a standard curve was created using a dilution series ranging from 0 ng/µL up to 100 ng/µL of genomic DNA.

**Fluorescence microscopy.** Histological studies of WT-GFP, *∆tri5-*GFP and mutants generated in this study, were investigated by fluorescence microscopy using MZ FLIII (Leica) microscope. A UV (ultra violet) lamp HAL 100 served as UV light source. GFP was excited at 480/40 nm and detected with a long pass filter at 510 nm. Zeiss LSM 780 laser scanning microscope (LSM) was used to determine the FgPE1 gene expression and protein localization on complete media, wheat media or detached wheat paleae as previously described in Boenisch and Schäfer, (2011). Image processing and generation of maximum intensity projections (MIP) of z-stacks were performed with Zeiss ZEN software (version 2010). The z-series 3D reconstructions were done using the surface rendering function of ZEN software.

**Extraction of aurofusarin from *F. graminearum* wild type and aurofusarin-deficient mutant *Δpks12*.** Fungal material of the WT strain and the aurofusarin-deficient mutant was harvested after 4 days from 50 mL CM liquid cultures. The respective mycelium was harvested using Myracloth, washed with 100 mL ddH_2_O and semi-dried using a filter paper. Around 1g of mycelium was transferred into a 2mL tube and supplemented with 1 mL potassium phosphate-buffer (50 mM, pH 7). After addition of 2 metal pearls (Ø 3mm), the solution was ground for 15 minutes using a retch mill. After centrifugation at 13.000 rpm for 15 minutes, the extracted supernatant was filter sterilized using a 0.22 µm Millex® GP filter.

**Analysis of fungal extracts via LC-MS**. The extracts of WT strain and *Δpks12* mutant were evaporated using a stream of N_2_. The dried metabolites were added to 200 µL HPLC-grade methanol with 1% formic acid (HCOOH) and resuspended by ultrasonication for 30 minutes with vortexing every 5 minutes. The samples were centrifuged to remove undissolved particles for 5 minutes at 16,000 g in a table top centrifuge at 20°C. 100 µL from the very top of the vial were transferred to HPLC tubes and the analysis performed on a Dionex UltiMate 3000 UHPLC equipped with a diode array detector (DAD) system hyphenated to a Q-TOF mass spectrometer. The samples were analysed with three different inject volumes 1 µL, 5 µL and 10 µL. For separation in the UHPLC system, a reversed-phase Kinetex C18 (100 mm, 2.1 mm, 2.6 μm) column was used and its temperature was maintained at 40 °C and a flow rate of 400 µL/min. The analytes were eluted using a gradient starting at 10% solvent B and increased to 100% solvent B over a period of 15 minutes. The used mobile phases consisted of MilliQ water with 20 mM formic acid and acetonitrile with 20 mM formic acid. The column was washed with 100% for 3 minutes and re-equilibrated for 2.4 minutes with 10% B before the next sample was injected. The analytes were detected via an online DAD (Dionex Ultimate 3000) detect from 200 to 600 nm and an online maXis 3G Qq-Oa-TOF (Bruker Daltronics GmbH). In the MS the analytes were ionized by electrospray operating in positive mode; capillary voltage at 4.5 kV, nebulizer gas at 2.4 bar, drying gas flow at 12 mL/min and a drying temperature of 220 °C. The MS was used in full scan mode in the mass range of 100-1000 Da. The instrument was calibrated using sodium formate (HCOONa) (Fluka, analytical grade). The obtained data were processed and handled using Compass Data Analysis v. 4.0 SP4 Build 281 (Bruker Daltronics). Bruker Daltronics Compass Isotopic Pattern was used for calculating isotopic patterns of the pseudo-molecular ion and adducts. The aurofusarin peak was identified by comparison with an authentic standard of aurofusarin and verified by the UV/VIS spectrums and MS-adduct pattern (M^+^H^+^ and M^+^Na^+^). In addition, a searching via extracted ion chromatograms (EIC) (571.08 +/- 0.02) in the mutant was performed, but it did not result in the detection of any aurofusarin.

**Bioactivity assay.** Liquid cultures of the organism listed in Table S12 were used for bioactivity assays. Bacteria were grown in LB liquid at 37°C and 200 rpm shaking. *P. teres* and *N. haematococca* were incubated in liquid CM at 28 °C. Growth of yeast-like fungi *S. cerevisiae*, *C. parapsilosis*, *P. pastoris* and *G. candidum* was performed in YPG at 28°C and at 37°C for *C. albicans*. Inoculation of 4 mL testing cultures was done using either 15 µL of an over-night culture (OD_595_ ca. 3, bacteria and yeasts) or either 10^4^ conidia or 100 µL of a mycelial pre-culture (filamentous fungi).

Cultures were supplemented with either 200 µL of *F. graminearum* extracts isolated using 1 mL potassium-phosphate buffer, or 200 µL potassium-phosphate buffer and incubated either over-night (bacteria and yeasts) or for 2 days (filamentous fungi). To check for inhibition capacity of the extracts, culture growth was measured either by OD_595_ (bacteria and yeasts) or by dry weight after lyophilisation for seven days (filamentous fungi).
